# Ingestion of Surface Residues Dominates Quaternary Ammonium Compounds (QACs) Exposure in Chinese Urban Homes: Evidence from Silicone Wristband Passive Sampling and Urinary Biomonitoring

**DOI:** 10.1021/acs.est.5c16557

**Published:** 2026-01-28

**Authors:** Min Hu, Li Li, Xiaozhen Zhang, Xixian Fang, Mengyao Ran, Zhong Lv, Md Mehedi Hasan Nafis, Zihao Zhang, Xi He, Haoran Xia, Sheng Wan, Yuge Liang, Jia Zhao, Xinrui Leng, Yao Cheng, Jianbang Xiang, Zongwei Cai, Guomao Zheng

**Affiliations:** † State Key Laboratory of Soil Pollution Control and Safety, Shenzhen Key Laboratory of Precision Measurement and Early Warning Technology for Urban Environmental Health Risks, School of Environmental Science and Engineering, 255310 Southern University of Science and Technology, Shenzhen 518055, China; ‡ State Key Laboratory of Environmental and Biological Analysis, Department of Chemistry, 26679 Hong Kong Baptist University, Kowloon, Hong Kong 999077, China; § School of Public Health, 6851 University of Nevada, Reno, Nevada 89557, United States; ∥ School of Public Health (Shenzhen), 26469 Shenzhen Campus of Sun Yat-Sen University, Shenzhen, Guangdong 518107, China; ⊥ Eastern Institute of Technology, Ningbo 315100, China

**Keywords:** quaternary ammonium compounds (QACs), silicone wristbands, urinary exposure biomarkers, ingestion of surface residues, relative source contribution (RSC)

## Abstract

The quantitative characterization of multiple exposure routes to quaternary ammonium compounds (QACs) remains underexplored. In this study, paired samples of indoor dust, bulk air, hand wipes, silicone wristbands, and urine were collected from 109 adults residing in urban homes from South China in 2023. First, seven urinary biomarkers, including hydroxylated and carboxylated metabolites of C10–C14 benzylalkyldimethylammonium compounds (BACs), were identified using a combined *in silico* and *in vitro* workflow. Then, 23 QACs, including 6 C8–C18 BACs, 6 dialkyldimethylammonium compounds (C8–C18 DADMACs), 6 alkyltrimethylammonium compounds (C8–C18 ATMACs), and 5 emerging QACs, were ubiquitously detected in various environmental matrices, including dust (median ∑QAC concentrations of 39.6 μg/g), bulk air (130 pg/m^3^), hand wipes (1420 ng for two hands), and silicone wristbands (225 ng/g), respectively. A significantly positive correlation was observed between the logarithmically transformed masses of QACs detected in silicone wristbands and those from dust, bulk air, and hand wipes (*r*: 0.564, *p* < 0.01). Moreover, urinary hydroxylated and carboxylated C10- and C12-BACs were significantly correlated with corresponding parent compounds in wristbands (*r*: 0.481–0.607, *p* < 0.01). Finally, back calculation from urinary exposure biomarkers revealed that ingestion of surface residues was the dominant exposure route for C10-, C12-, and C14-BACs, accounting for 3.7%, 49.6%, and 18% of total exposure, respectively. The findings from this study propose suitable urinary exposure biomarkers and silicone wristbands as useful indicators for accurate internal and external exposure assessment, respectively, and highlight the importance of ingestion of surface residues as a major exposure route.

## Graphical abstract

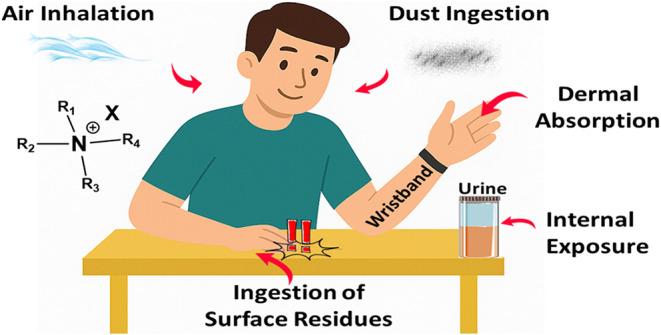

## Introduction

Quaternary ammonium compounds (QACs) are a class of chemicals widely used as disinfectants, antiseptics, preservatives, and detergents due to their antimicrobial properties.^1^ These chemicals have been ubiquitously detected in indoor dust,^2^ air,^3^ furniture surfaces,^4^ and dairy food,^5–7^ posing widespread human exposure. Studies have shown that exposure to QACs can lead to dermal and respiratory effects,^8,9^ developmental and reproductive toxicity,^10–12^ disruption of metabolic functions such as lipid homeostasis,^13,14^ and impairment of mitochondrial function.^15,16^ The widespread exposure and growing evidence of their adverse health impacts have raised significant public concern. In 2019, the Environmental Influences on Child Health Outcomes (ECHO) project, initiated by the US National Institutes of Health (NIH), classified QACs as a priority for biomonitoring in children.^17^ In 2021, Biomonitoring California also added QACs to its list of priority chemicals, calling for more research on human biomonitoring of these substances.^1^

Indoor dust has been identified as a major reservoir for QACs.^2,18^ However, our previous study observed a weak correlation between QAC levels in paired blood and dust samples, whereby we indicated the minimal (<1%) contribution of dust ingestion to the total human exposure to QACs.^19^ This suggests that other exposure routes, such as mouthing-mediated ingestion of surface residues of QACs (QAC molecules adsorbed on indoor surfaces or hand skin, or dissolving in the organic film or oil layer thereon) through hand-to-mouth contact, dietary intake, and inhalation, can be more significant.^20^ Additionally, accumulating modeling studies supports that QACs can partition between bulk air and disinfected surfaces, with potential exposure further through inhalation, skin contact, and hand-to-mouth interactions.^4,20^ Furthermore, existing evidence indicates that QACs can enter the food chain through their use in sanitizing food contact surfaces (e.g., dairy processing equipment, fruit and vegetable packaging lines, and food storage containers), serving as a significant source of dietary exposure.^21–24^ However, studies on the characterization of human exposure via multiple routes (e.g., dermal, inhalation, and ingestion) and quantitative analysis of their relative source contributions are limited.

While a comprehensive evaluation of all potential exposure pathways may theoretically provide complete quantitative analysis for multiroute exposure scenarios, this systematic approach can become prohibitively time-consuming in practice, potentially constituting a critical bottleneck in exposure assessment workflows.^25^ Thus, the “reverse dosimetry” technique that back-calculates intake doses from the internal chemical levels (e.g., concentrations of parent compounds in blood or those of metabolites in urine) is recommended to reflect aggregate exposure across multiple pathways more accurately.^26,27^ Reverse dosimetry relies on quantitative relationships between dose and concentration, which can be either computed by toxicokinetic models^28,29^ or determined experimentally.^30,31^ Recent studies have detected parent QACs in human blood and breast milk, with C12-BAC being the predominant compound.^3,32^ However, blood collection is invasive and may pose a challenge for large-scale epidemiological studies.^33^ The other methodological challenge in blood analysis is to minimize procedural blank contamination, so specialized pretreatment and analytical techniques (e.g., chromatographic delay columns) are required to enhance sensitivity and accuracy for quantifying parent QACs, which are present at relatively low levels in blood as observed in our previous studies.^3,19^ In contrast, QACs are rapidly metabolized in the liver,^34^ and their urinary metabolites typically present lower risks of contamination, which supports the use of urine as a practical matrix for biomonitoring.^35–37^ Alternatively, the measurement of QAC metabolites in blood may also provide complementary insights into internal exposure and biotransformation dynamics.

Additionally, understanding the relative importance of exposure pathways of QACs typically requires labor-intensive sampling methods, such as dust samplers, active/passive air samplers, and hand wipes. However, these methods are frequently limited by the variability of microenvironmental conditions and a lack of comprehensive data on long-term exposure patterns.^38^ In recent years, silicone wristbands have gained popularity as passive personal wearable samplers due to their cost-effectiveness, ease of use, and ability to assess human exposure to various semivolatile organic compounds (e.g., flame retardants, plasticizers, and pesticides).^39–43^ Moreover, wristbands have shown great value for semiquantitative assessments^44^ and for linking external exposure to urinary biomarkers.^39,45–47^ Despite their growing applications, no studies have yet focused on developing personal wearable samplers specifically for QACs.

In this study, we first applied *in silico and in vitro* techniques to explore potential metabolites of 18 traditional QACs, including 6 benzyl alkyldimethylammonium compounds (BACs), 6 dialkyldimethylammonium compounds (DADMACs), and 6 alkyltrimethylammonium compounds (ATMACs). We then proposed urinary biomarkers for QACs by incorporating biomonitoring techniques into pooled human urine. Furthermore, we analyzed paired samples of indoor dust and bulk air samples collected from 109 households in Shenzhen City, China, as well as paired hand wipes, silicone wristbands and urine samples collected from the residents of these homes (total n= 545 of matched dust, bulk air and hand wipes, wristbands and urine samples) for a suite of QACs and their metabolites. Our objective was to evaluate current QACs exposure patterns in humans, estimate the relative contributions of dust ingestion, inhalation, dermal absorption and ingestion of surface residues to the overall body burden, and propose a suitable personal wearable device and urinary biomarkers to accurately measure QACs exposure in future epidemiological studies.

## Materials and Methods

### Reagents and Materials

Detailed information about native standards, including full names, abbreviations, formulas, CAS numbers, vendors, and purities, is provided in Table S1. Four mass-labeled internal standards (*d*_7_-C12-BAC, *d*_7_-C14-BAC, *d*_9_-C10-ATMAC, and *d*_25_-C12-DADMAC) were purchased from Toronto Research Chemicals (Toronto, ON, Canada). The carboxylated and hydroxylated BAC isomers, along with two isotopic standards (*d*_3_-OH-C12-BAC and *d*_6_-COOH-C12-BAC, the deuterium substitution occurs at the methyl group bonded to the nitrogen atom, see the specific structures in Figure S1), were synthesized following the routes outlined in a previous study.^34^ All solvents and chemicals used in the present study were HPLC-grade or high-analytical grade. Oasis WCX cartridges (3 mL, 60 mg, 30 μm) were obtained from Waters Corporation (Milford, MA, USA). Human liver microsomes were purchased from Sekisui XenoTech Inc. (Lawrence, KS, USA). The NADPH regeneration system was obtained from Promega Corporation (Madison, WI, USA).

### Sample Collection

All samples, including hand wipes, silicone wristbands, bulk air, dust, and urine, were collected between September and November 2023 in Shenzhen, China. Participants (*n* = 109) were recruited from individuals living in the neighborhoods surrounding the Southern University of Science and Technology campus. Although some households included more than one resident, only one individual per household was selected and monitored. Each participant provided one sample of hand wipes, wristbands, bulk air, dust, and urine, resulting in a total of 545 samples (five samples per participant). The selection of polydimethylsiloxane (PDMS) as passive air samplers is based on the chamber saturation experiments using PDMS, silicone pad and polyurethane foam (PUF, Text S1 and Figure S2). The study was approved by the Southern University of Science and Technology Ethics Committee (institutional review board number 20220026), and all participants signed an informed consent form before participation. Each volunteer was visited twice during the sample collection period, with a 1 week interval between visits. During the first visit, volunteers were provided with a silicone wristband to wear and were instructed to complete a questionnaire, including information on demographics and behavioral data, as well as the frequency of using disinfectants or personal care products, such as shampoo, body wash and perfume. These products were subsequently categorized as QAC-containing or QAC-free by the researchers based on label declarations. No restrictions or interventions were imposed on the routine activities of participants, including handwashing frequency, either before or during the sampling period. A PDMS film as a passive air sampler was also hung in the volunteer’s indoor environment. The second visit was conducted to complete the collection of all samples (hand wipes, wristbands, bulk air, dust, and urine) from the volunteers. The effectiveness of PDMS as passive air samplers was compared to silicone pads and PUF, and the sampling duration of PDMS and silicone wristbands was predetermined in our laboratory chamber experiments. As PDMS samplers do not differentiate between QACs in the particle and vapor phases, the concentrations measured in PDMS are reported as bulk air which integrates particle and vapor phases. The details on the preparation and deployment of hand wipes, dust samplers, silicone wristbands, and PDMS are provided in Text S1 and Figures S2–S4.

All samples were kept in a cooler with ice packs before being delivered to the laboratory at the end of each sampling day. The environmental samples (dust, bulk air, hand wipes, and silicone wristbands) were stored at −20 °C, while the urine samples were kept at −80 °C until analysis.

### *In Vitro* Microsomal Incubations and Chemical Analysis

For the *in vitro* incubation of human liver microsomes (HLM), reactions were performed in triplicate experiments in separate 2 mL polypropylene (PP) tubes at 37 °C using methods described in our previous study.^48^ Briefly, a 200 μL reaction mixture containing 50 mM phosphate-buffered saline (PBS, pH 7.4) with 100 μL of human liver microsome protein at a final concentration of 1 mg/mL, 39 μL of PBS, 50 μL of NADPH solution A, 10 μL of NADPH solution B, and 50 μM substrate (final concentration) was delivered in 1 μL of DMSO. The elevated substrate concentration was employed to enhance metabolite coverage, although potential substrate competition cannot be entirely ruled out.^35^ 18 traditional QACs were divided into 6 groups based on the same chain length, including one BAC, DADMAC, and ATMAC, as long-chain parent QACs may shorten the alkyl chains and then generate short-chain QACs metabolites during the metabolic hydrolysis process.^35^ The mixtures were incubated at 37 °C in a temperature-controlled shaker at 120 rpm. After 1 h, 200 μL of ice-cold acetonitrile was added to terminate the reaction. The negative control samples were prepared with heat-inactivated microsomes to assess potential background interferences and nonenzymatic changes.

Consequently, the incubation mixtures were spiked with a surrogate standard mixture (*d*_7_-C12-BAC and *d*_9_-C10-ATMAC, 1 ng each) and extracted with 0.2 mL acetonitrile. Each extraction was followed by a 30 min ultrasonication treatment, after which the mixtures were centrifuged at 10,000 rpm for 20 min. The supernatant was then carefully aspirated and transferred to a new 2 mL PP tube. The extraction process was repeated, and the supernatant from both extractions was combined and stored at −20 °C. Prior to filtration, the pooled supernatant was transferred to a 2 mL vial, and the internal standards (*d*_7_-C14-BAC and *d*_25_-C12-DADMAC, 1 ng each) were added before the instrumental analysis.

### Sample Analysis

Approximately 2.5 g of the wristband was carefully cut from the whole wristband and placed in a 15 mL PP tube. Simultaneously, 50 mg of dust sieved through a 500 μm mesh, was weighed and placed into a 15 mL PP tube. The wristband crumb, sieved dust, the whole piece of PMDS, and hand wipes were put in a 15 mL PP tube separately. The samples were spiked with 20 ng of surrogate standards (*d*_7_-C12-BAC and *d*_9_-C10-ATMAC) and then extracted with 4 mL of acetonitrile for 30 min using sonication at room temperature. The extraction procedure was repeated twice, and the extracts from each sample were combined. The combined extracts were concentrated to ∼1 mL, filtered through 0.2 μm nylon syringe filters, and spiked with 20 ng of *d*_7_-C14-BAC and *d*_25_-C12-DADMAC prior to instrumental analysis.

For the urine samples, the pooled urine sample used for the discovery of urinary biomarkers was created by combining 1 mL of urine from 10 randomly selected volunteers out of the 109 participants. Both pooled (10 mL) and individual (2 mL) urine samples were spiked with surrogate standards (*d*_3_-OH-C12-BAC and *d*_6_-COOH-C12-BAC, 1 ng each) and subsequently loaded onto Oasis WCX cartridges preconditioned with 3 mL of methanol and 3 mL of water. The columns were washed with 3 mL of water and 3 mL of methanol. The target analytes were then eluted with 3 mL of 2% formic acid in methanol. The resulting extracts were evaporated to dryness using a steam of nitrogen blow, redissolved in 0.1 mL of acetonitrile, filtered through 0.2 μm nylon syringe filters, and spiked with 1 ng of *d*_7_-C14-BAC and *d*_25_-C12-DADMAC as internal standards before instrumental analysis. The urine specific gravity was determined by the refractometer (MASTER-SUR/Nα, ATAGO CO., Ltd., Japan), and urinary concentrations were adjusted accordingly.

### LC-QTOF-MS Analysis

Samples from *in vitro* microsomal incubations were analyzed using an Agilent 1290–6546 UPLC-QTOF-MS for the purpose of discovering urinary biomarkers. Chromatographic separation was conducted by a C18 column (ACQUITY UPLC BEH C18, 1.7 μm, 2.1 × 50 mm, Waters, Ireland), and the column temperature was kept at 30 °C. The mobile phase A was 0.1% formic acid with 5 mM ammonium acetate in water, and B was 0.1% formic acid in acetonitrile/isopropanol (40/60, v/v). The flow rate was set as 0.4 mL/min, and the injection volume was 5 μL. The gradient was linearly programmed as follows: 0–0.5 min, 10% B; 0.5–6 min, 100% B; 6–10 min, 100% B; 10–10.5 min, 10% B; 10.5–14.5 min, 10% B. The mass spectrometer was equipped with an electrospray ionization (ESI) source operating in the positive ion mode. The MS setup included a 25 psi nebulizer, 10 L/min gas flow, 300 °C sheath gas temperature, 2800 V capillary voltage, and 11 L/min sheath gas flow. Calibration of the QTOF MS was conducted with *m*/*z* 121.0508 and 922.0098 standards before each analysis to ensure mass accuracy. Both full scan and data-dependent acquisition (DDA) modes were employed under the following settings. In full scan mode, MS^1^ spectra were collected at a scan rate of 2 spectra/s over an *m*/*z* range of 50–1000, and mass accuracy below 5 ppm. For DDA mode, the acquisition rates for MS^1^ and MS^2^ were set to 6 and 5 spectra/s, respectively. Two precursor ions were selected for MS^2^ fragmentation in each acquisition cycle and fragmented at collision energies of 10, 20, and 40 eV, respectively.

### LC-MS/MS Analysis

Quantitative analysis of QACs and metabolites in urine and environmental samples, including dust, air, hand wipes, and silicone wristbands, was performed using an Agilent 1290–6470 UPLC-QqQ-MS. Separation was performed on a C18 column (ACQUITY UPLC BEH C18, 1.7 μm, 2.1 × 100 mm, Waters, Ireland). The mobile phases remained unchanged as those described above for LC-QTOF-MS analysis, while the mobile phase gradient was meticulously optimized to enhance the separation of QACs metabolites in urine sample analyses. The modified gradient was linearly adjusted as follows: 0 min, 10% B; 0–1 min, 25% B; 1–5 min, 40% B; 5–7 min, 100% B; 7–12 min, 100% B; 12.01–13.0 min, 10% B. The flow rate was set at 0.2 mL/min, and the injection volume was 5 μL. For the mass spectrometer settings, the gas and sheath gas temperatures were 325 and 350 °C, and the flow rates were 10 and 12 L/min, respectively. The nebulizer was set at 25 psi and the capillary was 3000 V. The details on MRM transitions, fragmentors, and collision energies for target analytes and surrogate and internal standards are provided in Table S2.

### Quality Assurance and Quality Control (QA/QC)

Field blanks (*n* = 3) were collected for each type of sample to examine potential background contamination during the sample collection and were treated as real samples in subsequent data processing. Specifically, the precleaned nylon socks, clean silicone wristbands, fresh hand wipes, PDMS samplers, and urine cups were carefully unsealed at the sampling site. Procedure blanks and matrix spike recovery samples were analyzed for six collected samples across the pretreatment process. Absolute recoveries of parent QACs in wristbands, hand wipes, dust, and bulk air ranged from 84–124%, 80–124%, 72–117%, and 86–127%, respectively. For urine samples, the recoveries of QAC metabolites ranged from 76 to 119%. The average surrogate recoveries were 94 ± 2% and 108 ± 4%, 102 ± 3% and 106 ± 6%, 96 ± 3% and 98 ± 5%, 104 ± 5% and 115 ± 7%, for *d*_7_-C12-BAC and *d*_9_-C10-ATMAC in dust, bulk air, handwipes, and wristbands, respectively. In urine, the recoveries were 49 ± 5% and 116 ± 3% for *d*_6_-COOH-C12-BAC and *d*_3_-OH-C12-BAC, respectively. All reported concentrations were subtracted from the average procedural blank concentrations, but were not corrected by surrogate recovery except for carboxylated BACs in urine, due to the relatively low recovery of *d*_6_-COOH-C12-BAC. The method detection limit (MDL) was calculated as the three times standard deviation of the target analyte detected in procedural blanks. If compounds were not observed in the procedural blanks, MDL was set as 3 times the signal-to-noise (S/N) based on the lowest calibration point. The details on matrix spike recoveries, surrogate recoveries, procedural blanks, MDLs, and field blanks are presented in Tables S3–S5.

### Data Analysis

Relative source contributions (RSCs) for specific exposure routes were calculated by dividing the estimated daily intake dose (EDI_*i*_, ng/kg/day) for each route by the total daily intake (TDI, ng/kg/day), as follows:

1 $${\mathrm{RSCs}=\mathrm{EDI}}_{i}/\mathrm{TDI}\times \mathrm{100\%}$$

where *i* represents ingestion of dust (*i*_dust_), inhalation (*i*_inhala_), dermal absorption (*i*_dermal_), and ingestion of surface residues (*i*_surface_), respectively. If the TDI value is lower than the corresponding total EDI (sum of estimated daily intake), the RSCs will be calculated based on the total EDI instead. The details of the calculation of the daily intakes through these exposure routes are provided in Text S2 and Tables S6.

The back calculation of total daily intake (TDI) was estimated from the urinary excretion of QACs metabolites using the following equation:^30^

2 $$\mathrm{TDI}=(C_{\mathrm{urine}}{\times \mathrm{UV}}_{\mathrm{excr}})\times \left({\mathrm{MW}}_{p}{/\mathrm{MW}}_{m}\right){/F}_{\mathrm{UE}}$$

where *C*_urine_ is the concentration of QAC metabolites in urine (ng/mL), and UV_excr_ is the daily urine excretion volume at 20 mL/kg bw/day for adults.^31,49^ *F*_UE_ is the molar fraction of the urine-excreted QAC metabolites with respect to their parent compounds, and MW_p_ and MW_m_ are the molecular weights of parent and corresponding metabolites, respectively. The *F*_UE_ values of C10-BAC, C12-BAC, and C14-BAC were calculated to be 0.0123, 0.006, and 0.007, respectively. Details of the *F*_UE_ calculation are provided in Text S3.

### Statistical Analysis

Airborne concentrations of QACs were estimated based on the masses accumulated in the PDMS passive samplers, as described in detail in Text S4. For the statistical analyses, concentrations below MDLs were imputed with MDL/2. Urine concentrations were normalized to specific gravity to account for differences in urine dilution. Chemical concentrations were log-transformed to reduce the skewness, as the untransformed data significantly deviated from the normal distribution required for regression analysis (Shapiro–Wilk *p* < 0.05, Table S7). Spearman correlations were first used to assess pairwise correlations among the four external matrices (dust, bulk air, hand wipes, and wristbands) for QAC congeners detected in more than half of the samples. Additionally, Spearman’s correlations were calculated to evaluate the magnitude of associations between chemical mass in wristbands and combined chemical mass in dust + air + handwipes, and between the silicone wristbands and urinary metabolites (for BACs). Simple linear regression models were used to explore the relationship of total QACs between wristbands (dependent variable) and the combined levels in dust, air, and handwipes (independent variable), and the relationships between specific wristband parent compounds and their corresponding urinary metabolites (C10-BAC and OH-C10-BAC/COOH-C10-BAC; C12-BAC and OH-C12-BAC/COOH-C12-BAC; C14-BAC and OH-C14-BAC/COOH-C14-BAC). Moreover, stepwise linear regression analysis was performed to adjust the influence of demographic and behavioral covariates on the dependent variables in the regression models. The Akaike Information Criterion (AIC) was used as the selection criterion to include or exclude covariates, aiming to minimize the AIC of the final model. Although stratified analyses were considered for some variables, such as gender, disinfectant use frequency, and QAC-containing product usage, the insufficient subgroup sample sizes limited the statistical power and interpretability of these analyses. Therefore, the results are not presented here. Multiple testing was corrected using both the Bonferroni correction and Benjamini–Hochberg false discovery rate (FDR) procedure. All statistical analyses were performed using R Studio.

## Results

### Population Characteristics

A description of the participants’ demographic and behavioral characteristics (*n* = 109) is presented in Table 1. Participants’ ages ranged from 18 to 48 years old (mean 27 ± 7 years), with 46% males and 54% females. Ninety-four percent of participants had attained a college education or higher, while 6% had a high school education or less. Most of the participants were nonsmokers (97%), while only 3% were smokers. Seventy-two percent of participants had a BMI within the normal range (18.5–24.9 kg/m^2^), while 17% were overweight (25–29.9 kg/m^2^) and 6% were obese (≥30 kg/m^2^). A total of 28% of participants spent their time at home less than 8 h per day, 66% spent 8–16 h, and 6% spent 16–24 h at home, respectively. Disinfectants were used by 44% of participants, with 19% using them more than once a week and 81% using them less than once a week. More than half of the participants washed their hands more than five times a day, while 42% washed their hands fewer than five times. About 92% of participants reported regular use of shampoo, 29% mentioned using perfume, and 58% had used products containing QACs.

**Table 1:** Summary of participants’ (*n* = 109) Demographic and Behavioral Characteristics

Demographic Characteristics	Average (±SD^a^)	*N*	Percentage, %	Behavioral characteristics	*N*	Percentage, %
*Age (years)*	27 ± 7			*Disinfectant use*		
*Gender*				Yes	48	44
Male		50	46	No	61	56
Female		59	54	*Disinfecting frequency*		
*Education*				More than once a week	21	19
High school or less		6	6	Less than once a week	88	81
College or higher		103	94	*Handwash frequency*		
*Smoking*				>5 times	63	58
Smoker		3	3	<5 times	46	42
Nonsmoker		106	97	*Shampoo use*		
*BMI (kg/m* ^*2*^)				Yes	100	92
Underweight, <18.5		5	5	No	9	8
Normal, 18.5–24.9		79	72	*Perfume use*		
Overweight, 25–29.9		19	17	Yes	32	29
Obese, >30		6	6	No	77	71
*Time at home (hours)*				QACs containing		
<8		30	28	Yes	63	58
8–16		72	66	No	46	42
16–24		7	6			

a SD: Standard deviation.

### Identification and Validation of Urinary Biomarkers for QACs Exposure

The workflow for the identification of urinary QACs biomarkers is provided in Figure S5. Briefly, this workflow is based on the phase I metabolic products predicted from an online prediction tool named BioTransformer (available at www.biotransformer.ca) and combined with the metabolic products of QACs that have been reported in previous studies.^35^ The metabolic processes and six types of metabolites for BACs, DADMACs, and ATMACs are summarized in Figures S6–S8. Based on the detection of metabolites in the pooled urine samples, seven hydroxyl and carboxylic acid metabolites of BACs, including ω–OH-C10-BAC, ω–OH-C12-BAC, (ω-1)–OH-C12-BAC, ω–OH-C14-BAC, ω-COOH-C10-BAC, ω-COOH-C12-BAC, and ω-COOH-C14-BAC, were identified and further synthesized for the quantitative analysis. Although certain metabolites of DADMACs and ATMACs were detected in the HLM reaction mixture, none were identified in the pooled urine samples. The details on the identification of biomarker candidates, *in vitro* QACs metabolite biosynthesis, and validation in pooled urine samples are provided in Texts S5–S6 and Table S8.

### Concentrations of QACs Metabolites in Urine Samples

All QAC metabolites were frequently detected in urine samples, with detection frequencies of each compound being more than 50% (Table 2). The total concentrations of these metabolites (∑mQACs concentrations) in urine ranged from 0.0145 to 21.8 ng/mL, with a median concentration of 0.182 ng/mL. COOH-C10-BAC was measured at a detection frequency of 96% and with concentrations ranging from <MDL to 10.3 ng/mL (median 0.0948 ng/mL). It was also the most abundant QAC metabolite, contributing 57% to the total median concentration of all QAC metabolites, followed by COOH-C12-BAC (27%) and COOH-C14-BAC (12%). Hydroxylated QAC metabolites were also detected but at lower detection frequencies (58–72%) compared to carboxylated metabolites (75–99%). Specifically, OH-C10-BAC, OH-C12-BAC [sum of ω–OH-C12-BAC and (ω-1)–OH-C12-BAC], and OH-C14-BAC were found in 72%, 58%, and 61% with median concentrations of 0.00134, 0.00444, and 0.003 ng/mL, respectively. These hydroxylated QAC metabolites consisted of less than 6% of the ∑mQACs concentrations in urine.

**Table 2:** Detection Frequencies (DF, %), Median (Med), Minimum (Min), and Maximum (Max) Concentrations of Quaternary Ammonium Compounds Metabolites (mQACs) Detected in Urine (ng/mL, Adjusted for Specific Gravity)

	DF	Med	Min	Max
***C10-BAC metabolites***				
COOH-C10-BAC	96	0.0948	<MDL	10.3
OH-C10-BAC	72	0.00134	<MDL	0.374
***C12-BAC metabolites***				
COOH-C12-BAC	99	0.0447	<MDL	13.8
OH-C12-BAC^a^	58	0.0044	<MDL	6.33
***C14-BAC metabolites***				
COOH-C14-BAC	75	0.0194	<MDL	0.363
OH-C14-BAC	61	0.003	<MDL	0.0431
**∑mQACs**	100	0.182	0.0145	21.8

a Sum of ω-OH-C12-BAC and (ω-1)-OH-C12-BAC.

### Concentrations of Parent QACs in Environmental Samples

Overall, all target analytes, including C8–18 BACs, C8–18 DADMACs, and C8–18 ATMACs, and 5 emerging QACs were frequently found in four types of environmental matrices (Table 3). Generally, most of the QACs were less frequently detected in bulk air compared to other environmental samples. The median ∑QAC concentrations of dust, bulk air, hand wipes, and silicone wristbands were 39.6 μg/g, 130 pg/m^3^, 1420 ng for two hands, and 225 ng/g, respectively. BACs were the most abundant QAC group across all four matrices (Figure 1), particularly in dust, wipes, and silicone wristbands, highlighting their widespread presence in indoor environments. ATMACs were the second most abundant QAC group in these samples, contributing to 6.4–65% of the ∑QAC concentration. DADMACs and other emerging QACs contributed to a minor portion of the total concentrations of QACs in these samples (<10%). Specifically, C12- and C14-BAC, the most frequently used disinfectants, were the predominant congeners across these samples with contributions of 48% and 13%, 18% and 7.2%, 46% and 11%, 76% and 11% in dust, bulk air, hand wipes, and silicone wristbands, respectively.

**Table 3:** Detection Frequencies (DF, %), Median (Med), Minimum (Min), and Maximum (Max) Concentrations of Quaternary Ammonium Compounds (QACs) Detected in Paired Dust (μg/g), Bulk Air (pg/m^3^), Hand Wipes (ng for Two Hands), and Silicone Wristbands (ng/g)

	Dust				Bulk air				Hand wipes				Silicone wristbands			
	DF	Med	Min	Max	DF	Med	Min	Max	DF	Med	Min	Max	DF	Med	Min	Max
***BACs***																
C8-BAC	95	0.037	<MDL	19.9	95	1.04	<MDL	15.1	37	0.012	<MDL	17.6	33	0.0456	<MDL	9.45
C10-BAC	100	0.0496	0.00255	4.02	100	1.39	0.675	14.8	100	2.06	0.21	44.9	60	0.359	<MDL	45.1
C12-BAC	100	12.3	0.854	672	100	19.3	1.81	2580	100	320	13.3	22400	100	125	1.78	12800
C14-BAC	100	3.21	0.218	400	100	7.93	1.19	1280	100	79	2.81	17800	100	18.2	0.712	4220
C16-BAC	100	0.191	0.024	25.9	100	1.52	0.554	25.4	95	4.92	<MDL	786	69	0.512	<MDL	87.5
C18-BAC	100	0.272	0.0216	16.6	100	2.76	0.789	32.3	100	6.83	0.984	1810	44	0.0511	<MDL	305
**∑BACs**	**100**	**16.5**	**1.17**	**1100**	**100**	**36.9**	**8.81**	**3870**	**100**	**520**	**19.1**	**35600**	**100**	**161**	**2.65**	**17100**
***DADMACs***																
C8-DADMAC	99	0.0678	<MDL	1.61	4	0.0549	<MDL	8.03	78	1.03	<MDL	2970	84	1.01	<MDL	78.5
C10-DADMAC	100	0.333	0.0151	72	18	0.054	<MDL	153	97	2.65	<MDL	5730	97	1.72	<MDL	1680
C12-DADMAC	99	0.0397	<MDL	6.54	100	0.939	0.171	14.4	99	1.37	<MDL	171	47	0.0403	<MDL	10.1
C14-DADMAC	100	0.0475	0.00287	3.37	100	0.826	0.393	7.52	100	1.93	0.137	538	41	0.019	<MDL	24.7
C16-DADMAC	100	0.406	0.03	21.7	100	1.11	0.391	13.8	100	11.3	0.174	1710	15	0.0281	<MDL	92
C18-DADMAC	100	0.585	0.0425	33.4	8	0.319	<MDL	28.9	65	1.19	<MDL	922	28	0.102	<MDL	17
**∑DADMACs**	**100**	**1.95**	**0.165**	**74.2**	**100**	**3.41**	**1.61**	**156**	**100**	**52.9**	**1.23**	**5870**	**100**	**6.1**	**0.00966**	**1700**
***ATMACs***																
C8-ATMAC	88	0.0116	<MDL	0.349	20	0.118	<MDL	8.13	99	1.59	<MDL	48	18	0.003	<MDL	2.15
C10-ATMAC	100	0.123	0.00596	1.81	100	11.3	6.37	121	100	12.6	0.212	101	99	1.84	<MDL	23.1
C12-ATMAC	100	0.876	0.0149	32.8	100	27.4	16.6	198	100	34.5	2.23	1440	95	4.26	<MDL	707
C14-ATMAC	99	0.148	<MDL	23.4	100	5.34	4.09	14.6	90	4.05	<MDL	503	99	1.34	<MDL	312
C16-ATMAC	100	2.5	0.0965	52.7	99	12.5	<MDL	714	100	52	2.5	13200	84	1.55	<MDL	172
C18-ATMAC	100	3.92	0.178	67.5	100	14.8	0.95	201	100	125	11.1	8230	77	1.58	<MDL	233
**∑ATMACs**	**100**	**8.19**	**0.394**	**112**	**100**	**76.4**	**40.5**	**804**	**100**	**308**	**23.7**	**18000**	**100**	**18.6**	**1.17**	**1030**
***Others***																
DDA	94	0.0813	<MDL	58.4	13	0.494	<MDL	53.9	85	3.1	<MDL	908	73	0.667	<MDL	36.4
BEC	98	0.289	<MDL	25.8	24	0.0174	<MDL	44.1	100	22	0.556	2480	93	6.09	<MDL	645
CPC	64	0.056	<MDL	28.4	10	0.321	<MDL	35.2	72	3.69	<MDL	2690	8	0.14	<MDL	33
C2:12-DADMAC	96	0.0286	<MDL	3.15	0	<MDL	<MDL	<MDL	83	0.926	<MDL	80	60	0.157	<MDL	45.2
C8:10-DADMAC	97	0.0755	<MDL	3.55	8	0.499	<MDL	9.13	34	3.89	<MDL	2150	77	0.879	<MDL	79.2
**∑QACs**	**100**	**39.6**	**1.8**	**1220**	**100**	**130**	**54.6**	**4190**	**100**	**1420**	**62.4**	**38300**	**100**	**225**	**5.69**	**17400**

**Figure 1:**
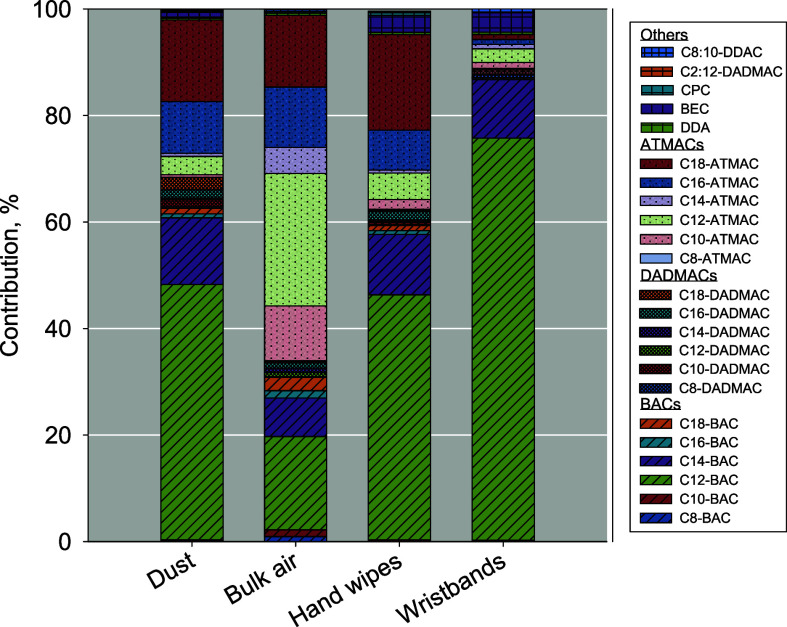
Percent contributions (%; calculated based on median concentrations) of individual QAC to the total QAC concentrations in dust, bulk air, hand wipes, and silicone wristbands.

### Concentration Correlations across External Matrices

The correlations between the logarithmically transformed concentrations of QACs detected in more than 50% of the samples across matrices were examined using Spearman correlation analysis, and the significance results are illustrated in a chord diagram (Figure 2). Specifically, as shown in Table S9, except for C12-DADMAC and C10-ATMAC, the correlation between hand wipes and silicone wristbands was generally the strongest for all QACs, with the highest correlations observed for C10–C16 BACs and C10-DADMAC (*r* = 0.52–0.72, *p* < 0.001), which are the main ingredients in QACs disinfectant products.^18^ Additionally, concentrations of most QACs in dust and silicone wristbands showed significant positive correlations (*r* = 0.34–0.53, *p* < 0.001). Though relatively weaker associations of QACs were found between bulk air and silicone wristbands, it is notable that the levels of C12–C16 BACs and longer chain ATMACs in the paired matrices were significantly and positively associated (*r* = 0.25–0.48, *p* < 0.001). Most of the significant correlations were observed between the concentrations of QACs in dust, bulk air, and hand wipes and the levels in silicone wristbands. In general, stronger correlations were observed between silicone wristbands and hand wipes, followed by silicone wristbands and dust, with moderate to weaker correlations between silicone wristbands and bulk air.

**Figure 2:**
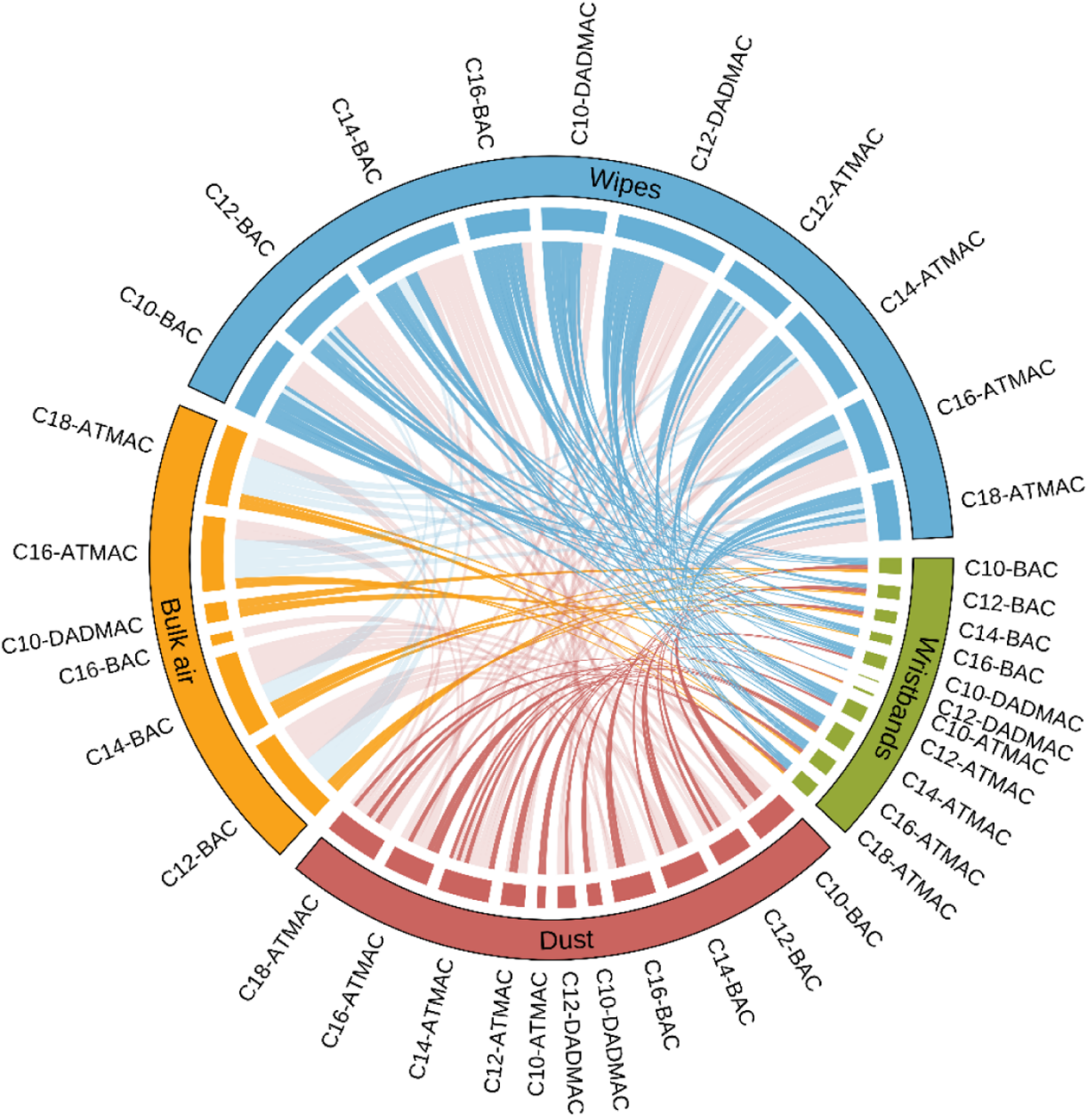
Chord diagram exhibiting the correlations across the four external environmental matrices, including silicone wristbands, hand wipes, dust, and bulk air.

Spearman’s correlation was calculated to estimate the association between QAC levels measured on wristbands and those in dust, air, and handwipes (Figure 3). Overall, a significantly positive correlation was observed between the logarithmically transformed masses (expressed in nanograms) of total QACs detected in silicone wristbands and those determined in an aggregate amount in dust, bulk air, and hand wipes, with an *r*-value of 0.564 and a *p*-value of <0.01. Of the 23 target chemicals, 21 (91%) showed significant correlations when tested by linear regression (*p* < 0.05) (Table S10). After adjustment for multiple testing using the Benjamini–Hochberg false discovery rate (FDR), all 21 associations remained significant (FDR-adjusted *p* < 0.05). Using the more conservative Bonferroni correction, 20 of the 21 chemicals remained significant, with only C18-DADMAC exceeding the adjusted significance threshold.

**Figure 3:**
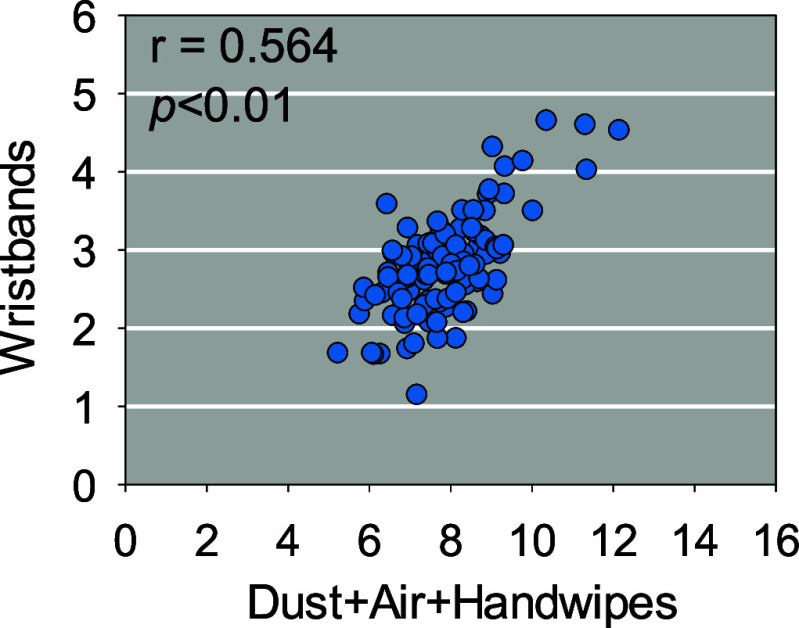
Scatterplots for log-transformed total QAC masses (expressed in nanograms) on silicone wristbands and the combined external exposure sources (dust, bulk air, and hand wipes). Each data point represents one participant. Spearman’s correlation coefficient (*r*) and *p-*value are reported.

### Associations of Silicone Wristband and Urine Concentrations

Due to the capacity of silicone wristbands as an integrated exposure measurement tool, Spearman’s correlation and linear regression analysis were used to determine the relationship between human external and internal exposure to QACs based on the concentrations of QACs detected in silicone wristbands and their metabolites in urine (Figure 4 and Table S11). As shown in Figure 4, the levels of parent C10- and C12-BACs captured by the wristband samples were significantly correlated with their corresponding hydroxy and carboxyl urinary biomarkers. Moreover, the hydroxy urinary metabolites for C10- and C12-BAC exhibited better associations with their parent BACs (*r* = 0.561 and 0.607 for C10- and C12-BAC, respectively) on silicone wristbands than the carboxyl urinary metabolites. However, no significant association was observed for COOH-C14-BAC/OH-C14-BAC in urine samples and C14-BAC on the silicone wristbands.

**Figure 4:**
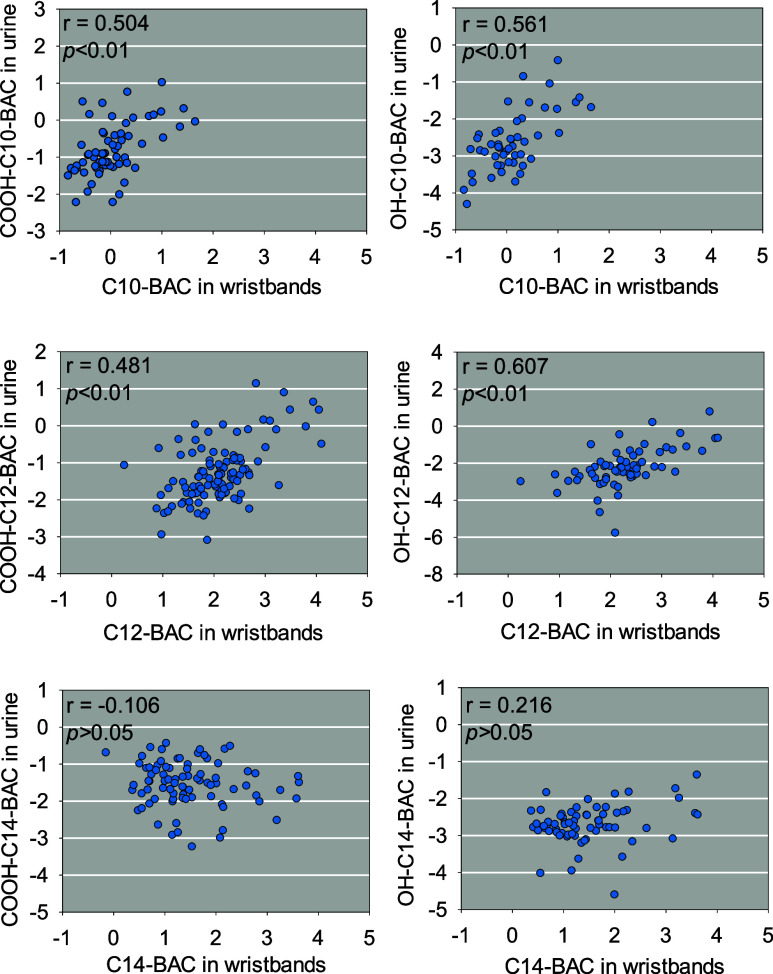
Scatterplots for C10, C12, and C14-BAC on wristbands with their respective urinary metabolites. Data are log-transformed, and Spearman’s correlations (*r*) and *p*-values for each association are provided.

### Relative Source Contributions (RSCs) of Multiple Exposure Routes to QAC Body Burden

As only the metabolites of C10-, C12-, and C14-BACs were consistently detected and quantified in the urine samples, the calculations of estimated daily intakes (EDIs), total daily intakes (TDIs), and RSCs were limited to these compounds (Table 4). The median TDIs, calculated based on the median concentrations of QACs metabolites in urine, were estimated as 2.44, 20.4, and 15.7 ng/kg bw/day for C10-BAC, C12-BAC, and C14-BAC, respectively. Overall, ingestion of surface residues plays a more important role (on average 2 to 2000 times higher) for all the BACs investigated than other exposure routes. For C12-BAC, ingestion of surface residues is the dominant exposure route, accounting for 50% of the total exposure. Dermal absorption exposure also played a non-negligible role for C12-BAC (26.6%) and C14-BAC (8.7%), while it contributed much less for C10-BAC (<2%). In contrast, inhalation contributes minimally to the internal exposure for all three compounds, with contributions ranging from 0.01 to 0.02%. Nevertheless, a substantial portion of exposure remains unaccounted for C10-BAC and C14-BAC, with undetermined RSCs of 94% and 64%, respectively.

**Table 4:** Estimated Daily Intakes (EDIs, ng/kg/day), Total Daily Intakes (TDIs, ng/kg/day) and Relative Source Contributions (RSCs, %) of C10-14 BACs via Dust Ingestion, Inhalation, Dermal Absorption and Ingestion of Surface Residues, Calculated from the Median, Mean, 5th and 95th Percentile Concentrations Measured in the Study Population (*n* = 109)

	EDIs				RSCs			
	median	mean	5th	95th	median	mean	5th	95th
***C10-BAC***								
Dust ingestion	0.0229	0.0957	0.00403	0.488	0.94	0.617	1.24	0.951
Inhalation	0.000336	0.000397	0.000211	0.000594	0.01	0.00256	0.0651	0.00116
Dermal absorption	0.042	0.127	0.0117	0.696	1.7	0.818	3.61	1.36
Ingestion of surface residues	0.09	0.247	0.0275	1.39	3.7	1.59	8.5	2.71
**TDI for C10-BAC**	**2.44**	**15.5**	**0.324**	**51.3**				
***C12-BAC***								
Dust ingestion	5.68	19.8	0.926	96.9	23.8	4.39	33.6	8.83
Inhalation	0.00466	0.023	0.000795	0.0657	0.02	0.00511	0.0289	0.00599
Dermal absorption	6.33	24.9	0.731	122	26.6	5.52	26.5	11.2
Ingestion of surface residues	11.8	49	1.1	242	49.6	10.9	39.8	22
**TDI for C12-BAC**	**20.4**	**450**	**1.86**	**1100**				
Dust ingestion	1.48	8.93	0.256	54.5	9.4	19.2	11.6	22.8
Inhalation	0.00192	0.00955	0.000474	0.0237	0.01	0.0205	0.0215	0.0099
Dermal absorption	1.37	11.2	0.178	57.4	8.7	24	8.06	24
Ingestion of surface residues	2.84	26.4	0.305	127	18	56.8	13.9	53.2
**TDI for C14-BAC**	**15.7**	**17.5**	**2.2**	**58.2**				

## Discussion

Previous studies on internal exposure assessment of QACs have primarily relied on blood samples.^3,19^ However, since QACs are rapidly metabolized, they are typically detected in urine but in the form of metabolites.^35–37^ In addition, these metabolites may also be excreted directly into the bile and subsequently eliminated through the feces.^50^ In this study, we combined *in silico* and *in vitro* approaches to propose seven urinary biomarkers for internal exposure to QACs, including ω–OH-C10-BAC, ω–OH-C12-BAC, (ω-1)–OH-C12-BAC, ω–OH-C14-BAC, ω-COOH-C10-BAC, ω-COOH-C12-BAC, and ω-COOH-C14-BAC. The number of identified biomarkers differs from those identified in previous studies, primarily due to the different conditions of *in vitro* HLM incubation and different instrumental analytical techniques.^37^ For example, Belova et al. identified additional one carbonyl metabolite of C12-BAC and four C10-DADMAC metabolites in urine, through a combination of *in vitro* experimentation with human liver microsomes and cytosols and determination of ion-mobility high-resolution mass spectrometry.^37^ Nonetheless, the identified metabolites in this study are the major congeners that have been frequently found in human urine and feces.^35–37,50^ The concentrations of these seven QAC metabolites detected in urine (∑_7_mQAC average concentrations range: <MDL–0.422 ng/mL) are comparable to those in the U.S. population (∑_8_mQAC average concentrations range: <MDL–0.35 ng/mL).^36^ Among these, COOH-C10-BAC is identified as the predominant urinary metabolite (>56%), followed by COOH-C12-BAC (>26%), whereas hydroxylated metabolites accounted for a smaller proportion (<6%). This profile closely resembles that observed in US cohort studies, which quantified eight QAC metabolites in urine samples collected from New York residents.^36^ The frequent detections of QAC metabolites in urine, as observed in both our investigation and previous research,^36,37,50^ further emphasize the widespread occurrence of QACs in the human body.

QACs are ubiquitously detected in indoor dust collected from the current study, similar to those reported in previous studies.^2,18,19^ The distribution pattern of QACs in dust was consistent with our previous study conducted in the same locations, where BACs, DADMACs, and ATMACs contributed on average 63%, 30%, and 6.0%, respectively.^2^ However, these findings differ from those reported in indoor dust collected from the United States (on average, 42%, 27%, and 31% for BACs, DADMACs, and ATMACs, respectively),^19^ and in Europe (on average, 46%, 27%, and 27% for BACs, DADMACs, and ATMACs, respectively).^51^ This variation suggests the different patterns of QAC uses across different regions, likely influenced by differences in environmental conditions, usage patterns of cleaning and disinfecting compounds, and regulatory frameworks.^1,52^ QACs are also detected in the bulk air with relatively low concentrations compared to those in dust. This is because QACs tend to partition into the particle phase in the air, which can be captured by PDMS, but with a relatively low portion. Interestingly, ATMACs were the most abundant QAC group in bulk air (65% of the ∑QAC concentrations), followed by BACs (31%) and DADMACs (3%). ATMACs are primarily utilized as essential components in hair care products to reduce static and increase softness, as well as in air fresheners for their stabilizing and preservative properties, not limited to disinfectants.^1,2^ Although the QACs levels detected in bulk air are relatively low, it should be noted that high concentrations of QACs can be formed as particles or aerosolized product droplets, especially when the QACs disinfectants are sprayed.^53^ Previous studies have also found that greater health risks can exist during inhalation compared to other exposure pathways. A study in mice demonstrated that inhalation of QAC aerosols, including benzalkonium chloride, hexadecyl trimethylammonium bromide, cetylpyridinium chloride (CPC), and dimethyldioctadecyl ammonium bromide (DDA), caused deep lung effects and inflammation, with benzalkonium chloride being the most potent among them.^54^ Moreover, occupational studies have linked QAC exposure to an increased risk of asthma and chronic obstructive pulmonary disease (COPD), further emphasizing the severe health implications of respiratory exposure to these chemicals.^55^

In addition to dust ingestion and inhalation, ingestion of surface residues and dermal absorption are considered to be important exposure pathways in the previous modeling study.^4^ Here, we applied hand wipes, a useful tool for assessing human exposure to semivolatile contaminants through ingestion of surface residues and dermal absorption, to capture the QACs residues on skin that might have originated from surface desks or directly from consumer products. The levels of QACs in hand wipes (median 1420 ng for two hands) are significantly higher than those of other contaminants, including perfluoroalkyl substances (1.51 ng),^56^ organophosphate esters (76.9 ng),^57^ polycyclic aromatic hydrocarbons (42.0 ng),^58^ and polybrominated diphenyl ethers (60.0 ng),^58^ but lower than phthalates (3960 ng).^59^ Given the relatively low QAC loadings observed in collected dust, we conclude that the majority of QACs detected in hand wipes likely originate from surface residues. Our observation is consistent with findings in an earlier modeling study, which showed that for involatile but highly water-soluble chemicals like QACs, surface residues are expected to account for approximately half of the chemical load on hands and contribute to over 90% of the total chemical intake via hand-to-mouth contact.^4^ For this reason, our result suggests ingestion of surface residues and dermal absorption could be major exposure pathways for QACs intake. Nonetheless, the temporal variability of QACs concentrations on hands over time is unknown, as only one hand wipe sample was analyzed per individual at a single time point.

To overcome this issue, the silicone wristbands have been deployed to capture QACs individually over a week, a suitable sampling duration before QACs can be saturated in wristbands (Figure S3). All analytes detected in silicone wristbands have comparable detection frequencies to those in other environmental samples, including hand wipes (Table 2). The strongest QACs concentration correlations between silicone wristbands and hand wipes (Figure 2) suggest that silicone wristbands and hand wipes capture similar QACs exposure patterns in indoor environments. Furthermore, the total masses of QACs in silicone wristbands are significantly correlated with that accumulated in dust + bulk air+ hand wipes (*r*: 0.564, *p* < 0.01; Figure 3), suggesting silicone wristbands can integrate multiple QAC exposure routes, including dust ingestion, inhalation, dermal absorption, and ingestion of surface residues. The weaker correlations between silicone wristbands and individual sample types (dust, bulk air, hand wipes) reveal the limitation of relying on a single matrix to represent total exposure and emphasize the need to consider multiple pathways in exposure studies. When comparing QACs in silicone wristbands and other matrices, the correlation was significant only for BACs and for ATMACs. The lack of correlation between DADMACs in wristbands and other matrices is likely due to the infrequent detection of long-chain DADMACs (detection frequency [DF] < 50%) in silicone wristbands (Table S9). It should be noted that QAC levels detected in silicone wristbands do not directly reflect absolute exposure in humans, due to compound-specific partitioning coefficients toward silicone materials. For example, chemicals with relatively lower affinity may require a longer time to reach equilibrium with their concentrations in the surrounding environment.^60^ Nonetheless, our findings highlight the application of silicone wristbands as an integrated exposure measurement tool for assessing QACs from diverse exposure routes over extended periods, which can effectively overcome the temporal variability limitations inherent in single-point hand wipe sampling. This methodology shows particular promise for large-scale epidemiological investigations, enabling cost-effective individual exposure assessment across diverse populations while maintaining high participant compliance for longitudinal cohort studies evaluating QAC-related health outcomes. The stronger correlations between the concentration of hydroxyl QAC metabolites in urine and their respective parent compounds in wristbands indicate that hydroxyl QAC metabolites are more suitable as internal biomarkers of human exposure to QACs from different potential environmental pathways. Given that carboxylated QAC metabolites are more abundant and detected at higher levels in actual human urine samples compared to hydroxylated metabolites, both hydroxy and carboxyl QAC metabolites should be considered as urinary biomarkers to provide comprehensive insights into how humans are exposed to BACs in their indoor environments. However, no significant association is observed for both COOH-C14-BAC and OH-C14-BAC in urine samples and C14-BAC on the wristbands. This may be due to the fact that, compared to C10- and C12-BAC, C14-BAC, with its longer alkyl chain, is more likely to be excreted through feces,^35,50,61^ which were not collected in this study.

Our findings also align with prior modeling research demonstrating mouth-mediated ingestion of dust-bound chemicals and surface residues as the primary pathway for postuse exposure to QACs, followed by dermal absorption and inhalation. While fecal data were not collected in this study, our TDI estimations were based on urinary excretion fractions (*F*_UE_), which represent the fraction of TDI excreted through urine, accounting for elimination pathways other than urination.^62^ This approach minimizes the risk of overestimation and supports the validity of our exposure assessment despite the absence of fecal measurements. The ingestion of surface residues holds particular significance for C12- and C14-BACs, the main ingredients in QACs disinfectant products, given their rising environmental prevalence in indoor settings^2,18,19,51,63^ and growing toxicological evidence linking them to neurodevelopmental and reproductive impairments.^12,64^ However, aggregated contributions from dust ingestion, inhalation, dermal absorption, and ingestion of surface residues accounted for <50% of total daily intakes for C10- and C14-BACs. The diminished contribution for C10-BAC may reflect hepatic metabolism producing OH-C10-BAC and COOH-C10-BAC from longer chain BAC, potentially leading to overestimation of TDI when calculated from urinary exposure biomarkers. The substantial undetermined exposure proportion for C14-BAC is likely due to the reduced bioavailability associated with longer alkyl chain lengths, as evidenced by fecal elimination rates (23–30%, 24–40%, and 37–47% for C10-, C12-, and C14-BACs, respectively).^63^ In addition, the RSC values for C10-BAC via dust ingestion remain consistent between the current study and our previous work, while those for C12- and C14-BAC exhibit notable increases of an RSC value from 0.03% to 23.8%, and from 0.03% to 9.4%, respectively.^19^ This discrepancy may result from methodological differences between our previous PROTEX-based forward dosimetry modeling and the current *F*_UE_-based reverse dosimetry modeling. In our prior PROTEX-based modeling, we estimated the contribution of dust ingestion to the total QAC exposure by comparing serum concentrations predicted under the assumption of dust ingestion alone with serum concentrations measured in field samples.^19^ By contrast, the current study back-calculated the contribution of dust ingestion from measured urinary concentration using reverse dosimetry based on *F*_UE_. Differences in matrices (serum parent compounds and urine metabolites) and associated levels of uncertainties in chemical determination and quantification, as well as uncertainties associated with parameters used in modeling (e.g., partition coefficients and biological half-lives used in the forward modeling, vs *F*_UE_ used in the backward modeling) may be responsible for the discrepancies in dust RSC estimates even for the same exposure route. Given the rapid metabolism of QACs, our urine-based assessment likely provides more reliable estimates of dust ingestion-derived RSCs. The elevated contribution of dust ingestion for C12-BAC, the dominant compound in commercial QACs disinfectant products, underscores the particular significance of this pathway for toddlers, who experience substantially higher dust ingestion rates than adults (0.06 vs 0.03 g/d, respectively).^65^

## Limitations

This study has several limitations. The data presented in this study are limited in terms of sample size, which restricts their statistical power to inform the correlations among different matrices. The current study did not analyze fecal samples, which may constitute the primary reservoir for QAC residues. Future work that combines urinary and fecal biomonitoring will therefore be essential for a complete assessment of aggregate QAC exposure. The difference in the back calculation of TDI using different biological samples suggests that urine and feces could complement each other well in reflecting the total body burden of QAC exposure. Finally, the information needed to quantify the magnitudes of other sources of QAC exposure, such as dietary consumption preferences for QAC-containing items, is not available in the current study. Such data are needed to evaluate the relative importance of other exposure pathways for QACs.

## Environmental Implications

This study provides detailed information on production use, and employs rigorous laboratory quality control procedures, offering a comprehensive assessment of the multiple exposure routes of QACs in indoor environments and highlighting their potential health impacts. We propose a set of urinary biomarkers for QAC exposure that enable accurate, noninvasive assessment of internal QAC exposure and address the analytical challenge of blank contamination associated with parent compound measurements in biological matrices. Moreover, these biomarkers correlate well with external exposure levels measured in silicone wristbands, offering a valuable tool for future population-based exposure and health effect studies. The use of personal wearable devices like silicone wristbands provides an integrated approach to assessing external exposure from multiple sources, supporting a more holistic understanding of QACs in indoor environments. This approach shows particular promise for large-scale epidemiological investigations, enabling cost-effective, longitudinal exposure assessment across diverse populations. The alignment between internal biomonitoring and external exposure modeling reinforces the value of integrating multiple assessment approaches to capture both external sources and internal body burden, thereby improving the accuracy of human health risk evaluations for QAC compounds. The observed variation in relative source contributions among BAC homologues highlights the necessity for homologue-specific exposure and toxicity data to further refine risk assessments of human exposure to QACs. Finally, given increasing evidence of the neurodevelopmental toxicity of QACs, particular attention should be directed toward vulnerable populations such as toddlers, who are more likely to experience frequent hand-to-mouth contact.

## Supplementary Material



## Notes

Details of sample preparation and deployment for dust, hand wipes, wristbands, and passive samplers; exposure assessment strategy; animal experiments and calculation of the fraction of urinary excretion; airborne QAC concentration estimation via PDMS samplers; identification, biosynthesis, and validation of exposure biomarkers; analytical methods and parameters for QACs and metabolites; quality control measures including blanks, recoveries, and detection limits; dose estimation parameters; Shapiro–Wilk test; intermatrix correlation analyses; and stepwise linear regression (PDF)
