# Supplementary material for: Ingestion of Surface Residues Dominates Quaternary Ammonium Compounds (QACs) Exposure in Chinese Urban Homes: Evidence from Silicone Wristband Passive Sampling and Urinary Biomonitoring
Source: Environ Sci Technol. 2026 Jan 28;60(5):3947–60. doi: 10.1021/acs.est.5c16557 (PMC12895516; doi:10.1021/acs.est.5c16557)
Supplement: Supplementary file 1 [file es5c16557_si_001.pdf]

Supporting Information

for

Ingestion of Surface Residues Dominates Quaternary Ammonium Compounds (QACs) Exposure in Chinese Urban Homes: Evidence from Silicone Wristband Passive Sampling and Urinary Biomonitoring

Min Hu[1,2], Li Li[3], Xiaozhen Zhang[1], Xixian Fang[4], Mengyao Ran[1], Zhong Lv[1], Md Mehedi Hasan Nafis[1], Zihao Zhang[1], Xi He[1], Haoran Xia[1], Sheng Wan[1], Yuge Liang[1], Jia Zhao[1], Xinrui Leng[1], Yao Cheng[1], Jianbang Xiang[4], Zongwei Cai[2,5]* and Guomao Zheng[1]*

[1]State Key Laboratory of Soil Pollution Control and Safety, Shenzhen Key Laboratory of Precision Measurement and Early Warning Technology for Urban Environmental Health Risks, School of Environmental Science and Engineering, Southern University of Science and Technology, Shenzhen 518055, China

[2]State Key Laboratory of Environmental and Biological Analysis, Department of Chemistry, Hong Kong Baptist University, Hong Kong 999077, China

[3]School of Public Health, University of Nevada, Reno, NV 89557, United States

[4]School of Public Health (Shenzhen), Shenzhen Campus of Sun Yat-sen University, Shenzhen, Guangdong 518107, China

[5]Eastern Institute of Technology, Ningbo, 315100, China

*To whom correspondence should be addressed.

Guomao Zheng, Email: zhenggm@sustech.edu.cn

Zongwei Cai, Email: zwcai@hkbu.edu.hk

Number of pages: 62

Number of texts: 6

Number of tables: 11

Number of figures: 70

Text S1. The preparation and deployment of hand wipes, dust samplers, silicone wristbands, and PDMS.

Hand wipes. The preparation and deployment of hand wipes followed the protocol from the previous studies.[1-3] Briefly, cotton twill wipes (7.5 × 7.5 cm, 2 mm thickness) were precleaned through ultrasonic extraction using methanol twice. Hand wipe samples were obtained by research personnel wearing gloves. For each participant, a sterile gauze pad was immersed in isopropyl alcohol, and the entire surface of both hands, spanning from the fingers to the wrists, was systematically wiped three times to ensure comprehensive sample collection. Wipes were transferred into a clean 15 mL PP tube and stored at –20°C until analysis.

Dust. The sample collection of dust followed the protocol from our previous studies.[4, 5] Before sampling, the nylon socks (25 μm pore size) were thoroughly cleaned with ultrapure water and methanol through ultrasonication, then air-dried overnight and covered with aluminum foil in a chemical hood. Dust samples were obtained using these pre-cleaned nylon socks connected to a Dyson V8 Fluffy Extra vacuum cleaner. After collection, the nylon socks containing the dust were wrapped in aluminum foil and sealed in polypropylene zip-lock bags.

Silicone Wristband. Silicone wristbands were purchased from a commercial online vendor in China and prepared following previously described protocols.[6] Prior to deployment, silicone wristbands were cleaned three times with HPLC-grade methanol, wrapped in pre-combusted aluminum foil, and stored in zip-lock bags until use. To optimize the sampling duration, a pilot study was conducted in which five

volunteers each wore three silicone wristbands sequentially over a three-week period, with one silicone wristband collected at the end of each week. Participants were instructed to wear the wristbands continuously for 7 days during all routine activities, including sleeping and bathing.[7] At the end of each sampling period, silicone wristbands were removed by trained staff wearing clean gloves and immediately placed into 50 mL centrifuge tubes for analysis.

The total QAC concentrations (∑QACs) in silicone wristbands increased progressively over the three-week period, reaching a plateau by the third week (range: 391–1960 ng/g; Figure S3). These results support the feasibility of using a one-week wearing period as an effective and practical duration for capturing personal exposure to QACs using silicone wristbands.

PDMS. To evaluate the optimal sampling material and deployment duration for passive QAC sampling, an exposure chamber experiment was conducted. Chambers (88.5 cm × 34.5 cm × 87.3 cm) were pre-cleaned with ultrapure water and isopropanol to eliminate background contaminants prior to deployment. The experiments were performed over a four-week period in sealed exposure chambers. A passive doser, consisting of an aqueous solution containing 18 traditional QACs (1 g/L), was placed centrally on the chamber floor. Control chambers were loaded with an equal volume of deionized water under identical conditions.

Three types of passive sampling materials, including polydimethylsiloxane (PDMS, 10 × 10 cm, 0.1 mm thickness), silicone pads (10 × 10 cm, 1 mm thickness), and polyurethane foam (PUF, 13.5 cm diameter, 1.4 cm thickness), were suspended

approximately 80 cm above the doser to avoid direct contact. All materials were purchased from an online retailer in China. After 1, 2, 3, and 4 weeks of exposure, triplicate samples of each material were retrieved from both QAC and control chambers for analysis, following the procedures described in the Sample Analysis section.

Total QAC uptake by PDMS increased with exposure duration, reaching a maximum at 3 weeks (20.9–79.7 ng; Figure S2). In contrast, no QACs were detected in silicone pads, likely due to their limited sorption capacity for airborne QACs. PUF samplers were incompatible with the analytical workflow, as ultrasonication caused material dissolution during extraction. These findings support the use of PDMS as a suitable passive air sampling medium for QACs, with a one-week deployment offering a practical balance between sensitivity and operational feasibility.

During the field sampling campaign, PDMS films were suspended vertically at 1.5 m above floor level to minimize the gravitational deposition of coarse particles (Figure S4).[8] After a 7-day exposure period, the PDMS samplers were carefully retrieved by gloved research staff and transferred into pre-cleaned 15 mL polypropylene tubes for subsequent chemical analysis.

Text S2. Exposure assessment.

Four main QACs exposure pathways of humans in indoor environments were considered: dust ingestion, air inhalation, dermal absorption, and hand-to-mouth contact.

The dose through the ingestion of dust[9] was calculated using Eq (1)

$$EDI_{dust} = (C_{dust} * R_{Ing}) * T/BW \qquad (1)$$

The dose through the inhalation of air[10] was calculated using Eq (2)

$$EDI_{inhala} = (C_{air} * R_{Inh} * F_{\text{uptake-Inh}}) * T/BW \qquad (2)$$

The dose through the dermal absorption[11, 12] was calculated using Eq (3)

$$EDI_{dermal} = (Q_{hw}/HSA * SA * F_{uptake\text{-}dermal}) * T/BW \qquad (3)$$

The dose through the hand-to-mouth contact[11] was calculated using Eq (4)

$$EDI_{htm} = (Q_{hw} * TF_{htm} * H_{contact\text{-}area} * f_{htm}) * T/BW \qquad (4)$$

Therefore, the dose through ingestion of surface residues was calculated using Eq (5)

$$EDI_{surface} = ED_{Ihtm} - EDI_{dust} \qquad (5)$$

Where,

C_{dust} = QACs concentration in dust (ng/g)

C_{air} = QACs concentration in air (pg/m^3/d)

Q_{hw} = the total QAC mass present on the hands based on the concentrations in the hand wipes (pg)

HSA = the hand surface area of the participants (cm^2)

SA = the surface area of the skin exposed to pollutants (cm^2)

R_{Ing} = dust ingestion rate (g/d)

R_{Inh} = air inhalation rate (m^3/d)

$F_{\text{uptake-Inh}}$ = the uptake fraction of QACs absorbed through the air (%)

$F_{uptake\text{-}dermal}$ = the uptake fraction of QACs absorbed through the skin (%)

TF_{htm} = the efficiency of the QAC mass transfer at each contact from hand to mouth (%)

$H_{contact\text{-}area}$ = the proportion of the hand contact area in each event (%)

f_{htm} = the frequency of hand-to-mouth events (events/hour)

T = the time spent at home (day)

BW = body weight (kg)

Text S3. The animal experiment, animal sample analysis and calculation of F_{UE}.

Male Sprague-Dawley (SD) rats aged 7-8 weeks were purchased from Shenzhen Huateng Biomedical Technology Co., Ltd. C10-BAC was dissolved in pure water and administered by gavage (Ig) and intravenous injection (Iv) (Ig) at a dose of 0.5 mg/kg, respectively. The rats were housed at Shenzhen Huateng Biomedical Technology Company at room temperature (22−25 °C) and 40−60% relative humidity. They were maintained on a 12 h light/dark cycle, with food and water provided ad libitum. In total, 6 SD rats were acclimatized in a laboratory environment for 3 days prior to treatment and were randomly assigned to two exposure groups (n = 3 rats/group, housed in two metabolic cages). The two exposure groups were administered gavage and an intravenous injection of C10-BAC at a concentration of 0.5 mg/kg, respectively. Blood samples were collected for exposure periods of 0 h, 0.2 h, 0.5 h, 1 h, 1.5 h, 2 h, 4 h, 8 h, 10 h, 12 h and 24 h. All the samples were freeze-dried and kept at -20°C before analysis.

0.2 mL blood of each rat was spiked with surrogate standards (d_7-C14-BAC, d_{25}-C12-DADMAC, d_3-OH-C12-BAC, and d_6-COOH-C12-BAC) and then extracted with 4 mL of acetonitrile for 30 minutes using sonication at room temperature. The extraction procedure was repeated twice, and the extracts from each sample were

combined. The combined extracts were concentrated to ~1 mL and filtered through 0.2 μm nylon syringe filters prior to instrumental analysis. Hydroxylated and carboxylated metabolites of three BACs were analyzed by an Agilent 1290–6470 UPLC-QqQ-MS as described in the Section of LC-MS/MS Analysis in Method and Materials. The urinary excretion fraction % of three BACs was calculated using the following equation:

$$AE_X = AUC_{Ig} / AUC_{Iv} \quad (6)$$

$$F_{UE} = AE_X * F_{metabolism, X} * F_{X, Y} * F_{urination, Y} \quad (7)$$

Where, AUC_{Ig} and AUC_{Iv} are the predicted integrated area under the time-dependent concentration curve (sum of hydroxyl- and carboxylated BAC metabolites) via IV and Ig, respectively. AE_X is a fraction of parent compound X absorbed into the systemic circulation. $F_{metabolism, X}$ is the fraction of parent X that is metabolized in systemic circulation. $F_{X, Y}$ is the fraction of metabolized X that is converted to metabolite Y. $F_{urination, Y}$ is the fraction of metabolite Y in systemic circulation excreted via urination. The sources of these parameters are provided in Table S6.

Text S4. Calculation of airborne QACs concentration using PDMS passive sampler.

The sampling rate (Rs, $m^3/d/dm^2$) was assumed to be constant over time during the linear uptake phase.[13] During the linear uptake phase, the mass (M, ng) of the analyte sequestered by the PDMS passive sampler can be calculated using the following equation:[14]

$$M = C_{air} * Rs * A_{PDMS} * T \quad (8)$$

C_{air}: concentrations of compounds in the air, pg/m; Rs, sampling rate, $m^3/d/dm^2$; A_{PDMS}: The effective adsorption area of the sampler, 2 dm^{-2}; T: sampling time, 7 days.

A normalized generic Rs of 7.7 $m^3/d/dm^2$ was used to calculate the airborne concentrations of QACs in this study.[15]

Text S5. *In vitro* QAC metabolite biosynthesis.

Parent compounds of QACs were incubated with human liver enzymes to facilitate the identification of potential metabolites. To isolate the metabolite signals, the primary signals obtained from the HLM reaction mixture were processed by subtracting the blank signals. The probable metabolite signals were confirmed as QACs-related metabolites through the analysis of MS^2 fragmentation patterns using LC-QTOF mass spectrometry. Subsequently, these structure-related metabolite signals were verified as potential biomarkers of QACs exposure by analyzing pooled urine samples from volunteers. Overall, 56 potential urinary biomarkers were identified, including mono- and dihydroxylated forms (+1O and +2O), as well as more extensively oxidized and desaturated species (+1O, −2H; +2O, −2H; +3O, −2H; and −2H), as summarized in Table S8. Corresponding chromatograms and MS/MS spectra are provided in Figures S9–S64. Overall, the metabolites biosynthesized from BAC were the most abundant, followed by those from DADMAC, with ATMAC showing the relatively lowest frequency. The distribution pattern of C12-BAC metabolites observed in this study was consistent with a recent study.[16] Meanwhile, the results of the cited study indicated that the metabolism of C16-ATMAC is slower compared to C12-BAC and C10-DADMAC, with the +O metabolite showing the highest relative abundance after 3 hours of *in vitro* human liver microsomes (HLM) incubation.[16] Mono-hydroxylated metabolites (+O) were widely detected for traditional QACs, except for C8-ATMAC. However,

dihydroxylated metabolites (+2O) were found for C12-18 BACs and C12-14 DADACs, but not for 6 ATMACs. Moreover, carbonyl (+1O-2H) and carboxylated (+2O-2H) metabolites were frequently detected for C10-18 BACs, C10-16 DADMACs, and limited observation for C12-14 ATMAC. Additionally, our findings suggested that desaturated metabolite (-2H) was frequently observed across different types of QACs, whereas this metabolite was only observed for C8-BAC, C8-ATMAC and C10-ATMAC using ion mobility-mass spectrometry in a previous study.[16] In contrast, we did not detect any further oxidized and desaturated metabolite (+3O, -2H) for all QACs investigated, while this type of metabolite was frequently found for C14-C18 BACs, C10-C14 ATMACs, and C8-DADMAC in this previous study. In addition, Belova et al. only observed +3O, -2H metabolite for C10-DADMAC among three target QACs (C12-BAC, C16-ATMAC, and C10-DADMAC), and this higher oxidation states metabolite increased in relative abundance over incubation time.[16] In conclusion, these discrepancies could be attributed to differences in analytical instruments and incubation time.

Text S6. Validation of exposure biomarkers.

In this study, we applied the HLM reaction mixture of QACs as hypothetical standards to analyze ten pooled specimens randomly selected from volunteers. Only C10-14 BACs with their mono-hydroxylated (+O) and carboxylated (+2O, -2H) metabolites were detected in real urine samples (Figures S65-S70). Interestingly, mono-hydroxylated C12-BAC exhibited two isomers, including ω-hydroxy (terminal, Figure S68, RT=3.183 min) and (ω-1)-hydroxy metabolites (Figure S68, RT=2.86 min). A

previous study reported that BACs were oxidized exclusively along the alkyl chain region by cytochrome P450 (CYP) enzymes to ω-hydroxy (terminal) and (ω-1)-hydroxy metabolites.[17] The detections of ω-COOH-C10-BAC, ω-COOH-C12-BAC, and ω-OH-C12-BAC have also been reported in previous studies,[18] further validating the accuracy of our method. Therefore, seven BAC metabolites, including ω-OH-C10-BAC, ω-OH-C12-BAC, (ω-1)-OH-C12-BAC, ω-OH-C14-BAC, ω-COOH-C10-BAC, ω-COOH-C12-BAC, and ω-COOH-C14-BAC were further synthesized for the qualitative and quantitative analysis of human urine in our study.

Table S1. Detailed information of full name, abbreviation, formula, register number (CAS), supplier, and purity (%) of the target QAC analytes. IS: Internal standard; SS: surrogate standard.

Full name	Abbreviation	Formula	CAS	Supplier	Purity
**List of Compounds for Environmental Sample Analysis.**					
Benzyldimethyloctylammonium chloride	C8-BAC	C17H30ClN	959-55-7	DRE	98
Benzyldimethyldecylammonium chloride	C10-BAC	C19H34ClN	965-32-2	DRE	98
Benzyldimethyldodecylammonium chloride	C12-BAC	C21H38ClN	139-07-1	Macklin	99
Benzyldimethyltetradecylammonium chloride	C14-BAC	C23H42ClN	147228-81-7	Aladdin	98
Benzyldimethylhexadecylammonium chloride	C16-BAC	C25H46ClN	122-18-9	Picasso	98
Searyldimethylbenzylammonium chloride	C18-BAC	C27H50ClN	122-19-0	Macklin	98
Dioctyldimethylammonium bromide	C8-DADMAC	C18H40BrN	3026-69-5	MERDA	97
Didecyldimethylammonium bromide	C10-DADMAC	C22H48BrN	2390-68-3	Adamas	98
Didodecyldimethylammonium bromide	C12-DADMAC	C26H56BrN	3282-73-3	Aladdin	98
Dimethylditetradecylammonium bromide	C14-DADMAC	C30H64BrN	68105-02-2	Macklin	97
Dihexadecyldimethylammonium bromide	C16-DADMAC	C34H72BrN	70755-47-4	J&K	97
Dimethyldioctadecylammonium bromide	C18-DADMAC	C38H80BrN	3700-67-2	MERDA	99
Octyltrimethylammonium chloride	C8-ATMAC	C11H26ClN	10108-86-8	Macklin	99
Decyltrimethylammonium bromide	C10-ATMAC	C13H30ClN	2082-84-0	Macklin	99
Dodecyltrimethylammonium chloride	C12-ATMAC	C15H34ClN	112-00-5	MERDA	99
Tetradecyltrimethylammonium chloride	C14-ATMAC	C17H38ClN	4574-04-3	Adamas	99
Hexadecyltrimethylammonium chloride	C16-ATMAC	C19H42ClN	112-02-7	MERDA	99
Octadecyltrimethylammonium chloride	C18-ATMAC	C21H46ClN	112-03-8	Adamas	98
Cetylpyridinium chloride	CPC	C21H38ClN	6004-24-6	MERDA	98
Benzethonium chloride	BEC	C27H42ClNO2	121-54-0	Energy	99
Diallyldimethylammonium chloride	DDA	C8H16ClN	7398-69-8	Adamas	60
Dodecylethyldimethylammonium bromide	C2:12-DADMAC	C16H36BrN	68207-00-1	MERDA	99
Decyldimethyloctylammonium chloride	C8:10-DADMAC	C20H44ClN	32426-11-2	MERDA	80
benzyldimethyldodecylammonium-d_7 (SS)	d_7-C12-BAC	C21H38ClN		TRC	99
decyltrimethylammonium-d_9 bromide (SS)	d_9-C10-ATMAC	C13H30ClN		TRC	99
denzyldimethyltetradecylammonium-d_7 chloride (IS)	d_7-C14-BAC	C23H42ClN		TRC	99
Di-n-dodecyldimethylammonium bromide-d_{25} (IS)	d_{25}-C12-DADMAC	C26H56BrN		TRC	99
**List of Compounds for Urine Sample Analysis.**					
ω-Hydroxyl-benzyldimethyldecylammonium chloride	OH-C10-BAC	C19H34NO			95
ω-Hydroxyl-benzyldimethyldodecylammonium chloride	ω-OH-C12-BAC	C21H38NO			95
(ω-1)-Hydroxyl-benzyldimethyldodecylammonium chloride	(ω-1)-OH-C12-BAC	C21H38NO			95
ω-Hydroxyl-benzyldimethyltetradecylammonium chloride	OH-C14-BAC	C23H42NO			95
ω-Carboxylic acid-benzyldimethyldecylammonium chloride	COOH-C10-BAC	C19H32NO2			95
ω-Carboxylic acid-benzyldimethyldodecylammonium chloride	COOH-C12-BAC	C21H36NO2			95
ω-Carboxylic acid-benzyldimethyltetradecylammonium chloride	COOH-C14-BAC	C23H40NO2			95
ω-Hydroxyl-benzyldimethyldodecylammonium-d_3 chloride (SS)	d_3-OH-C12-BAC	C21H35D3NO			95
ω-Carboxylic acid-benzyldimethyldodecylammonium-d_6 chloride (SS)	d_6-COOH-C12-BAC	C21H30D6NO2			95

Table S2. The optimized MRM transitions, fragmentors (Frag), and collision energies (CE) for target analytes and surrogate and internal standards analyzed under ESI (+) mode.

Cpd Name	Precursor Ion	Product Ion	Frag (V)	CE (V)
C8-BAC	248.2	91	88	29
	248.2	65.1	88	77
C10-BAC	276.3	184	103	21
	276.3	91.1	103	33
C12-BAC	304.3	212	113	25
	304.3	91	113	41
C14-BAC	332.3	240	122	25
	332.3	91.1	122	41
C16-BAC	360.4	268	146	25
	360.4	91.1	146	41
C18-BAC	388.4	296.3	127	29
	388.4	91	127	45
C8-DADMAC	270.3	158.2	156	29
	270.3	71.1	156	33
C10-DADMAC	326.4	186.0	151	33
	326.4	71.1	151	37
C12-DADMAC	382.4	214	181	37
	382.4	71.1	181	41
C14-DADMAC	438.5	242	151	41
	438.5	71.1	151	49
C16-DADMAC	494.6	270	151	49
	494.6	71.1	151	53
C18-DADMAC	550.6	298	175	53
	550.6	71.1	175	57
C8-ATMAC	172.2	85.1	132	21
	172.2	71.1	132	25
C10-ATMAC	200.2	85.1	127	21
	200.2	71.1	127	25
C12-ATMAC	228.3	85.1	137	25
	228.3	71.1	137	25
C14-ATMAC	256.3	85.1	142	29
	256.3	71.1	142	29
C16-ATMAC	284.3	85.1	132	29
	284.3	71.1	132	33
C18-ATMAC	312.4	85.1	142	33
	312.4	71.1	142	33
CPC	305.3	81.1	145	30
	305.3	80.1	145	34
BEC	413.3	91.1	145	58
	413.3	72.1	145	34
DDA	127.1	85.1	105	14
	127.1	84.1	105	14
C2:12-DADMAC	243.3	75.1	145	30
	243.3	74.2	145	30

**Cpd Name**	**Precursor Ion**	**Product Ion**	**Frag (V)**	**CE (V)**
C8-10-DADMAC	299.4	187.2	175	30
	299.4	159.2	175	30
OH-C10-BAC	292.3	200.2	150	10
	292.3	91.1	150	20
ω-OH-C12-BAC	320.3	228.2	150	10
	320.3	91.1	150	20
(ω-1)-OH-C12-BAC	320.3	228.2	150	10
	320.3	91.1	150	20
OH-C14-BAC	348.3	256.3	150	10
	348.3	91.1	150	20
COOH-C10-BAC	306.2	214.2	150	10
	306.2	91.1	150	20
COOH-C12-BAC	334.3	242.2	150	10
	334.3	91.1	150	20
COOH-C14-BAC	362.3	270.2	150	10
	362.3	91.1	150	20
d_{25}-C12-DADMAC	407.6	239.4	225	42
	407.6	215.2	225	42
d_7-C14-BAC	339.4	98.1	127	41
	339.4	70.1	127	97
d_7-C12-BAC	311.3	212	122	25
	311.3	98.1	122	37
d_9- C10-ATMAC	209.3	85.1	127	21
	209.3	71.1	127	25
d_3-OH-C12-BAC	323.3	231.3	150	10
	323.3	61.1	150	20
d_6-COOH-C12-BAC	340.3	247.2	150	10
	340.3	63.1	150	20

Table S3a. Target analytes and their mean analyte levels measured in field blanks (FBs, n = 3), procedural blanks (PBs, n = 6), and method detection limits (MDLs, µg/g) for dust (ng/g), bulk air (pg/m^3), hand wipes (ng) and silicone wristbands (ng/g). n.d: not detected.

	FBs	PBs	MDLs	FBs	PBs	MDLs	FBs	PBs	MDLs	FBs	PBs	MDLs
	Dust, ug/g			Bulk air, pg/m^3			Hand wipes, ng			Silicone wristbands, ng/g		
C8-BAC	0.00017	0.00473	0.0016	0.493	0.458	0.269	0.206	0.0972	0.0239	0.12	0.115	0.0912
C10-BAC	0.00525	0.00338	0.0004	0.526	0.485	0.163	0.442	0.144	0.107	0.045	0.0479	0.0449
C12-BAC	0.00281	0.0317	0.0442	3.8	1.49	2.08	16.4	0.695	0.443	0.355	0.167	0.286
C14-BAC	0.00073	0.0126	0.0152	1.23	0.497	0.488	3.6	0.182	0.0835	0.159	0.107	0.0973
C16-BAC	0.00133	0.00871	0.0041	0.532	0.467	0.257	0.618	0.171	0.0185	0.175	0.146	0.0401
C18-BAC	0.00099	0.0181	0.0076	1.36	1.23	0.852	1.33	0.538	0.126	0.353	0.318	0.102
C8-DADMAC	n.d.	n.d.	0.0002	n.d.	n.d.	0.11	0.279	n.d.	0.0121	n.d.	n.d.	0.00454
C10-DADMAC	0.00133	0.00253	0.0059	0.167	0.0778	0.108	0.511	0.0566	0.167	0.0282	0.0151	0.0148
C12-DADMAC	0.00031	0.00231	0.0047	0.454	0.186	0.0677	0.174	0.0645	0.0466	0.141	0.0682	0.0806
C14-DADMAC	0.00033	0.00454	0.0025	0.227	0.25	0.148	0.224	0.0984	0.0652	0.0697	0.0752	0.038
C16-DADMAC	0.00410	0.00808	0.0014	0.349	0.491	0.752	1.77	0.257	0.0734	0.0958	0.128	0.0561
C18-DADMAC	0.00895	0.0104	0.0021	0.0699	0.511	0.638	1.64	0.171	0.0356	0.0597	0.14	0.203
C8-ATMAC	0.00007	n.d.	0.0001	0.22	0.137	0.237	0.372	n.d.	0.00905	n.d.	n.d.	0.00599
C10-ATMAC	n.d.	0.0201	0.0084	4.98	3.53	1.59	11.4	5.07	4.21	1.15	0.943	0.672
C12-ATMAC	0.00144	0.0105	0.0035	9.28	1.64	0.276	1.88	0.529	0.108	0.297	0.255	0.0762
C14-ATMAC	0.00377	0.0114	0.0004	1.83	1.93	0.205	0.938	0.624	0.105	0.281	0.253	0.0305
C16-ATMAC	0.00038	0.00719	0.0101	2.5	1.6	1.01	1.68	1.07	0.746	0.23	0.215	0.374
C18-ATMAC	0.03711	0.0097	0.0147	2.42	0.931	0.987	1.72	0.693	0.899	0.257	0.351	0.567
BEC	n.d.	n.d.	0.0001	n.d.	n.d.	0.0347	1.32	n.d.	0.0105	n.d.	n.d.	0.00428
CPC	n.d.	n.d.	0.0039	0.0961	n.d.	0.641	1.46	n.d.	0.00398	n.d.	n.d.	0.279
DDA	n.d.	n.d.	0.0012	0.43	0.32	0.989	1.34	0.0516	0.108	0.0981	0.168	0.0627
C2:12-DADMAC	n.d.	n.d.	0.0001	n.d.	n.d.	0.0388	n.d.	n.d.	0.0513	n.d.	n.d.	0.00779
C8:10-DADMAC	0.00015	n.d.	0.0060	n.d.	n.d.	0.997	0.254	n.d.	1.76	n.d.	n.d.	0.121

Table S3b. Target metabolites and their mean analyte levels measured in procedural (n = 6, ng/mL) and field blanks (n = 3, ng/mL) and method detection limits (MDLs, ng/mL) for human urine (ng/mL).

	Procedural blanks	Field blanks	MDLs
COOH-C10-BAC	0.0172	0.0115	0.0156
OH-C10-BAC	0.00346	0.00251	0.00268
COOH-C12-BAC	0.00732	0.00426	0.00414
ω-OH-C12-BAC	0.00932	0.0039	0.00789
(ω-1)-OH-C12-BAC	0.00132	0.00046	0.000994
COOH-C14-BAC	0.0312	0.0201	0.0226
OH-C14-BAC	0.0132	0.00798	0.00681

Table S4a. Matrix spike recoveries (%) of target analytes in environmental samples, including wristbands, hand wipes, dust, and bulk air (mean, standard error [SE]).

QACs	Dust		Bulk air		Hand wipes		Wristbands	
	Mean	SE	Mean	SE	Mean	SE	Mean	SE
C8-BAC	99%	7%	99%	4%	95%	1%	95%	2%
C10-BAC	98%	7%	98%	3%	93%	1%	90%	2%
C12-BAC	106%	8%	106%	4%	103%	2%	97%	2%
C14-BAC	101%	7%	97%	4%	99%	4%	88%	2%
C16-BAC	112%	9%	91%	4%	87%	2%	90%	3%
C18-BAC	93%	9%	93%	7%	96%	4%	90%	2%
C8-DADMAC	84%	6%	107%	3%	102%	2%	89%	2%
C10-DADMAC	104%	5%	115%	3%	113%	3%	98%	3%
C12-DADMAC	85%	5%	107%	9%	100%	5%	92%	4%
C14-DADMAC	72%	4%	105%	4%	92%	5%	111%	7%
C16-DADMAC	75%	5%	112%	8%	108%	4%	88%	3%
C18-DADMAC	97%	8%	86%	4%	80%	5%	89%	1%
C8-ATMAC	103%	7%	106%	4%	105%	2%	96%	2%
C10-ATMAC	107%	5%	114%	4%	117%	4%	104%	2%
C12-ATMAC	108%	8%	102%	4%	97%	1%	91%	4%
C14-ATMAC	114%	8%	103%	5%	102%	1%	95%	2%
C16-ATMAC	117%	6%	119%	5%	124%	2%	111%	2%
C18-ATMAC	114%	6%	107%	4%	99%	2%	97%	1%
BEC	100%	7%	101%	4%	97%	1%	97%	3%
CPC	116%	6%	111%	3%	107%	2%	95%	3%
DDA	106%	5%	127%	4%	116%	4%	124%	2%
C2:C12-DADMAC	107%	6%	112%	8%	95%	1%	89%	3%
C8:C10-DADMAC	83%	6%	103%	3%	98%	2%	84%	2%

Table S4b. Matrix spike recoveries of target metabolites in urine samples (mean, standard error [SE]).

**BAC metabolites**	Mean	SE
COOH-C10-BAC	95%	4%
OH-C10-BAC	76%	5%
COOH-C12-BAC	106%	5%
ω-OH-C12-BAC	105%	4%
(ω-1)-OH-C12-BAC	119%	13%
COOH-C14-BAC	99%	6%
OH-C14-BAC	114%	11%

Table S5. Mean surrogate recoveries (with their standard errors [SE]).

Surrogate	Dust		Bulk air		Handwipes		Wristbands		Urine	
	Mean	SE	Mean	SE	Mean	SE	Mean	SE	Mean	SE
d_7-C12-BAC	94%	2%	102%	3%	96%	3%	104%	5%		
d_9-C10-ATMAC	108%	4%	106%	6%	98%	5%	115%	7%		
d_6-COOH-C12-BAC									49%	5%
d_3-OH-C12-BAC									116%	3%

Table S6. Parameters used for calculating estimated daily intakes and relative source contributions.

Parameter	Value	References
R_{Ing}	0.03 g/d	US EPA 2011[19]
R_{Inh}	15.7 m^3/d	US EPA 2011[19]
$F_{uptake\text{-}Inh}$	100%	100%[20]
HSA	865 cm^2	This study
SA	2564 cm^2	US EPA 2011[19]
$F_{uptake\text{-}dermal}$	C10-BAC: 45%<br>C12-BAC: 43%<br>C14-BAC: 38%	Based on 3D-HSE percutaneous permeation experiments[21]
TF_{htm}	50%	Somrutai Poothong et al., 2019[11]
$H_{contact\text{-}area}$	5%	Somrutai Poothong et al., 2019[11]
f_{htm}	2 events/h	Somrutai Poothong et al., 2019[11]; Stapleton et al., 2008[22]; Zhao et al., 2022[23]
T	1 day	Zheng et al., 2020[9]
BW	65 kg	This study
AE_X	1.74%	This study
$F_{X,Y}$	C10-BAC: 99.9%<br>C12-BAC: 99.5%<br>C14-BAC: 97.9%	This study
$F_{metabolism,X}$	C10-BAC: 93.8%<br>C12-BAC: 89.8%<br>C14-BAC: 96.0%	PROTEX model[5]
$F_{urination,Y}$	C10-BAC: 75.3%<br>C12-BAC: 38.5%<br>C14-BAC: 43.0%	PROTEX model[5]

Table S7a. Shapiro–Wilk test results for QACs across matrices (only compounds detected in ≥ 50 % of the samples are included).

QACs	Dust		Bulk air		Hand wipes		Wristbands	
	Statistic	*p*-value	Statistic	*p*-value	Statistic	*p*-value	Statistic	*p*-value
C8-BAC	0.099	7.84E-23	0.313	2.58E-20	0.731	3.33E-07		
C10-BAC	0.319	3.03E-20	0.351	8.20E-20	0.579	3.49E-16	0.325	3.68E-20
C12-BAC	0.398	3.79E-19	0.232	2.50E-21	0.440	1.58E-18	0.308	2.20E-20
C14-BAC	0.305	2.05E-20	0.196	9.51E-22	0.297	1.59E-20	0.315	2.69E-20
C16-BAC	0.282	1.04E-20	0.373	1.67E-19	0.317	2.89E-20	0.397	3.65E-19
C18-BAC	0.368	1.41E-19	0.429	1.07E-18	0.235	2.69E-21		
C8-DADMAC	0.538	6.37E-17			0.087	5.90E-23	0.351	8.19E-20
C10-DADMAC	0.267	6.72E-21			0.186	7.22E-22	0.166	4.24E-22
C12-DADMAC	0.162	3.87E-22	0.500	1.40E-17	0.171	4.81E-22		
C14-DADMAC	0.244	3.51E-21	0.345	6.89E-20	0.124	1.46E-22		
C16-DADMAC	0.302	1.86E-20	0.465	3.86E-18	0.384	2.41E-19		
C18-DADMAC	0.355	9.27E-20			0.289	1.25E-20		
C8-ATMAC	0.506	1.78E-17			0.446	1.97E-18		
C10-ATMAC	0.585	4.52E-16	0.294	1.46E-20	0.842	2.02E-09	0.573	2.69E-16
C12-ATMAC	0.362	1.16E-19	0.340	5.90E-20	0.491	1.01E-17	0.248	3.88E-21
C14-ATMAC	0.166	4.22E-22	0.553	1.17E-16	0.336	5.19E-20	0.116	1.21E-22
C16-ATMAC	0.613	1.59E-15	0.285	1.12E-20	0.339	5.66E-20	0.548	9.57E-17
C18-ATMAC	0.654	1.13E-14	0.649	9.21E-15	0.474	5.42E-18	0.445	1.88E-18
DDA	0.088	2.31E-13			0.214	1.84E-18	0.397	4.11E-18
BEC	0.336	6.05E-23			0.412	1.52E-21	0.329	3.65E-19
CPC	0.322	5.19E-20			0.283	6.11E-19		4.12E-20
C2:C12-DADMAC	0.213	3.32E-20			0.371	1.05E-20	0.192	
C8:C10-DADMAC	0.429	1.46E-21					0.371	8.44E-22

Table S7b. Shapiro–Wilk test results for QAC metabolites in urine (only compounds detected in ≥ 50 % of the samples are included).

Metabolites	Urine	
	Statistics	*p*-value
COOH-C10-BAC	0.334	4.88E-20
OH-C10-BAC	0.194	8.96E-22
COOH-C12-BAC	0.264	6.04E-21
ω-OH-C12-BAC	0.126	1.55E-22
(ω-1)-OH-C12-BAC		
COOH-C14-BAC	0.647	8.25E-15
OH-C14-BAC	0.427	1.01E-18

Table S8. Summary of observed QAC microsomal metabolites (√, Observed; X, Not Observed).

	+1 O	+2 O	+1 O, -2 H	+2 O, -2 H	+3 O, -2 H	-2 H
				BACs		
C8	√	×	×	×	×	√
C10	√	×	√	√	×	√
C12	√	√	√	√	×	√
C14	√	√	√	√	×	√
C16	√	√	√	√	×	√
C18	√	√	√	×	×	√
				DADMACs		
C8	√	×	×	×	×	×
C10	√	√	√	×	×	√
C12	√	√	√	√	×	√
C14	√	√	√	√	×	×
C16	√	×	√	×	×	√
C18	√	×	×	×	×	√
				ATMACs		
C8	×	×	×	×	×	×
C10	√	×	×	×	×	√
C12	√	×	√	√	×	√
C14	√	×	√	×	×	√
C16	√	×	×	×	×	√
C18	√	×	×	×	×	×

Table S9. Spearman correlation coefficients (r) for the associations among the log-transformed QACs concentrations in dust, bulk air, silicone wristbands, and handwipes.[a]

	Dust/Air	Dust/Wipes	Air/Wipes	Dust/Wristbands	Air/Wristbands	Wipes/Wristbands
C10-BAC	0.11	0.39***	0.14	0.41***	0.15	0.52***
C12-BAC	0.51***	0.45***	0.44***	0.38***	0.41***	0.69***
C14-BAC	0.42***	0.39***	0.43***	0.43***	0.43***	0.72***
C16-BAC	0.34***	0.34***	0.22*	0.28**	0.25**	0.63***
C10-DADMAC		0.59***		0.48***		0.72***
C12-DADMAC	0.017	0.37***	-0.055			
C10-ATMAC	-0.077	0.17	0.043	0.34***	0.095	-0.0018
C12-ATMAC	0.22*	0.43***	0.22*	0.34***	0.11	0.55***
C14-ATMAC	0.22*	0.47***	0.11	0.47***	0.19	0.60***
C16-ATMAC	0.45***	0.61***	0.47***	0.53***	0.48***	0.73***
C18-ATMAC	0.43***	0.56***	0.47***	0.42***	0.44***	0.65***

[a]: Only QACs detected in more than 50% of the samples were included in the analysis. * indicates statistically significant correlations at *p*-value <0.05; ** indicates statistically significant correlations at *p*-value <0.01; *** indicates statistically significant correlations at *p*-value <0.001.

Table S10. Stepwise linear regression results for wristbands and dust + air + hand wipes. The correlation of chemical mass between the wristband and dust + air + hand wipes.

Source		Estimate, β	Predictor Variable's	Adjusted p-value	Adjusted p-value	R-Squared	Adjusted R-
ΣQACs (Dust + Air + Hand Wipes), ng	Wristbands, ng		p-value	(FDR)	(Bonferroni)		Squared
C8-BAC	C8-BAC	0.2383	1.2646E-09	2.2921E-09	3.6674E-08	0.3243	0.3046
C10-BAC	C10-BAC	0.6104	2.3020E-12	6.0688E-12	6.6757E-11	0.3891	0.3713
C12-BAC	C12-BAC	0.3256	3.4143E-17	1.9803E-16	9.9015E-16	0.5025	0.4930
C14-BAC	C14-BAC	0.3732	3.9874E-21	1.1564E-19	1.1564E-19	0.5731	0.5690
C16-BAC	C16-BAC	0.4950	1.8498E-11	4.4703E-11	5.3643E-10	0.3598	0.3411
C18-BAC	C18-BAC	0.6068	5.4063E-11	1.1199E-10	1.5678E-09	0.3442	0.3316
C8-DADMAC	C8-DADMAC	0.2657	3.2076E-05	4.0443E-05	9.3019E-04	0.2684	0.2322
C10-DADMAC	C10-DADMAC	0.3266	9.7562E-18	7.0732E-17	2.8293E-16	0.5189	0.5097
C12-DADMAC	C12-DADMAC	0.3628	9.1718E-08	1.3299E-07	2.6598E-06	0.2390	0.2317
C14-DADMAC	C14-DADMAC	0.5004	5.3769E-09	8.6628E-09	1.5593E-07	0.2780	0.2712
C16-DADMAC	C16-DADMAC	0.2108	0.0002	0.0002	0.0048	0.1495	0.1247
C18-DADMAC	C18-DADMAC	0.0793	0.0100	0.0107	0.2892	0.0873	0.0698
C12-ATMAC	C12-ATMAC	0.3693	8.6990E-08	1.3277E-07	2.5227E-06	0.2831	0.2623
C14-ATMAC	C14-ATMAC	0.2374	8.6406E-13	2.5058E-12	2.5058E-11	0.4017	0.3902
C16-ATMAC	C16-ATMAC	0.4643	1.4136E-19	2.0497E-18	4.0994E-18	0.5946	0.5787
C18-ATMAC	C18-ATMAC	0.4149	2.4188E-14	7.7938E-14	7.0144E-13	0.5200	0.5061
DDA	DDA	0.3358	2.0295E-09	3.4620E-09	5.8854E-08	0.4104	0.3622
BEC	BEC	0.2152	1.1367E-05	1.4984E-05	0.0003	0.2419	0.2121
CPC	CPC	0.0734	3.7291E-05	4.5060E-05	0.0011	0.2001	0.1768
C2:12-DADMAC	C2:12-DADMAC	0.2978	0.0005	0.0005	0.0137	0.1708	0.1383
C8:10-DADMAC	C8:10-DDAC	0.3407	1.3648E-06	1.8848E-06	3.9580E-05	0.2968	0.2546
∑QACs	∑QACs	0.3967	1.3133E-15	6.3476E-15	3.8086E-14	0.4645	0.4489

Table S11. Stepwise linear regression results for wristbands and urine.
The correlation of chemical concentrations between wristband and human urine (BACs only).

Source		Estimate, β	Predictor Variable's *p*-value	Adjusted *p*-value (FDR)	Adjusted *p*-value (Bonferroni)	R-Squared	Adjusted R-Squared
Wristbands, ng/g	Urine, ng/mL						
C10-BAC	OH-C10-BAC	1.0420	3.4700E-09	1.0400E-08	2.0804E-08	0.5798	0.5225
C10-BAC	COOH-C10-BAC	0.6905	4.0500E-06	6.0700E-06	2.4285E-05	0.3572	0.325
C12-BAC	OH-C12-BAC*	0.8432	1.5900E-07	3.1700E-07	9.5114E-07	0.4719	0.4256
C12-BAC	COOH-C12-BAC	0.6556	1.5300E-10	9.1800E-10	9.1763E-10	0.3354	0.3227
C14-BAC	OH-C14-BAC	0.2234	0.0045	0.0054	0.0272	0.2442	0.1687
C14-BAC	COOH-C14-BAC	-0.1300	0.1131	0.1131	0.6784	0.1758	0.1216

*: sum of ω-OH-C12-BAC and (ω-1)-OH-C12-BAC.

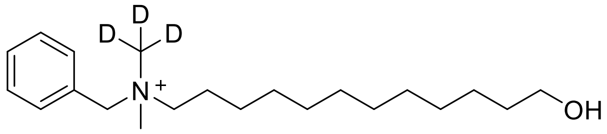

d3-OH-C12-BAC

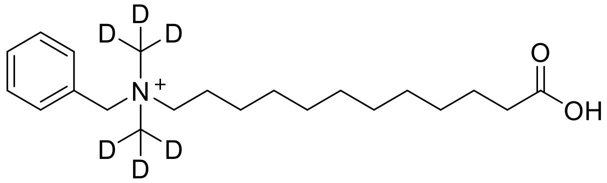

d6-COOH-C12-BAC

Figure S1. Structures of the synthesized d3-OH-C12-BAC and d6-COOH-C12-BAC in this study.

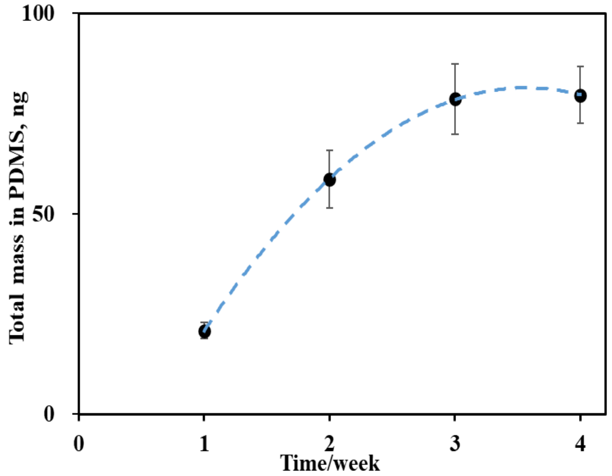

Figure S2. The total mass of 18 traditional QACs in the PDMS sheet (ng) over multiple sampling weeks in the chamber.

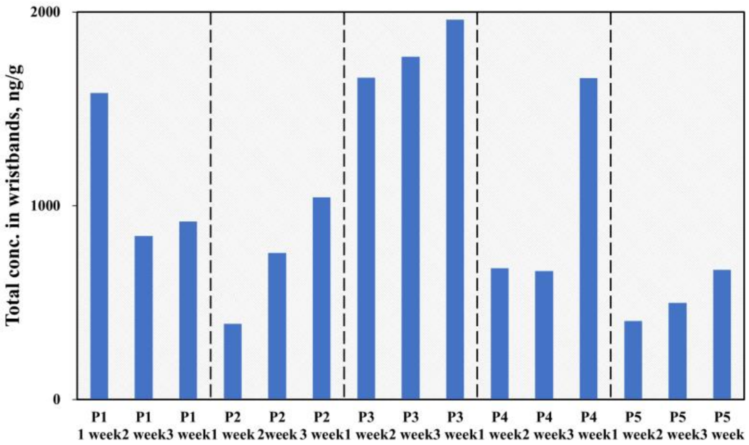

Figure S3. The total concentration of 18 traditional QACs in silicone wristbands (ng/g) across five participants over multiple sampling weeks. (P1, P2, P3, P4 and P5 were volunteers in our research group).

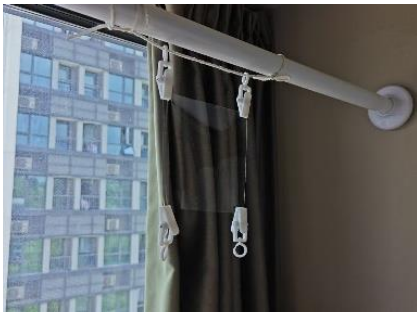

PDMS

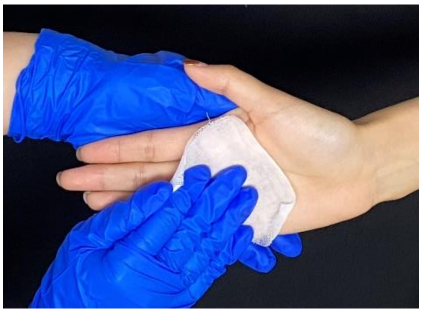

Hand wipes

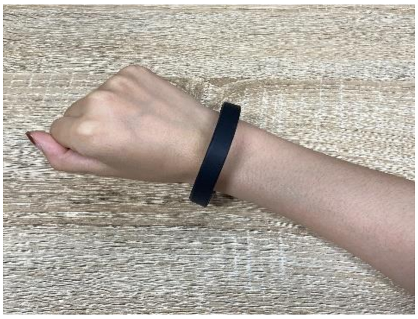

Silicone Wristband

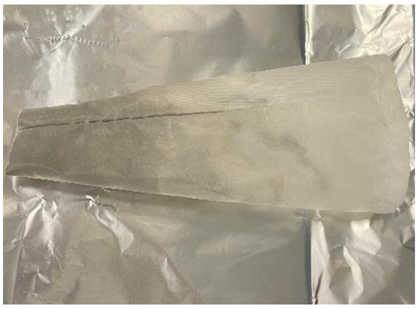

Dust

Figure S4. Field sampling setup and the deployment for PDMS, hand wipes, silicone wristbands and dust.

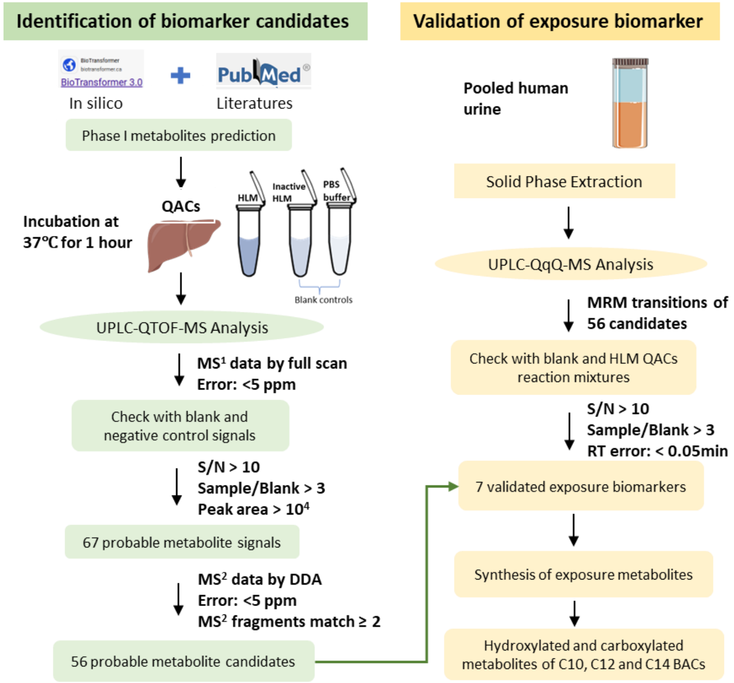

Figure S5. Workflow of QACs exposure marker discovery and validation using UPLC-QTOF and UPLC-QqQ MS.

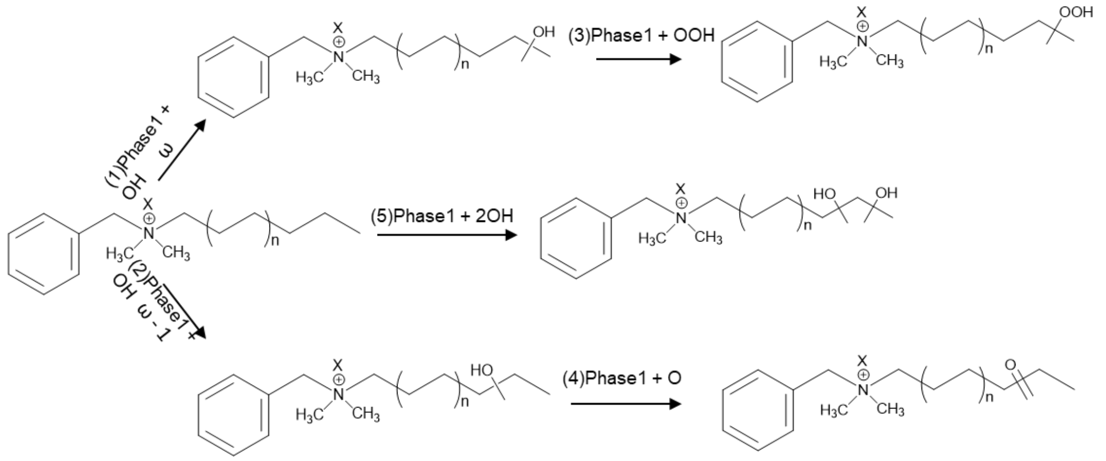

Figure S6. Summary of proposed BACs metabolism by HLM. n=4, 6, 8, 10, 12, 14.

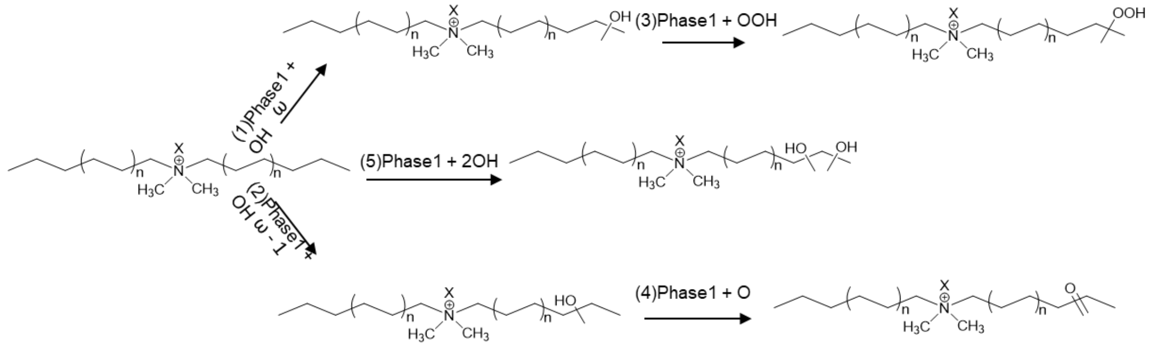

Figure S7. Summary of proposed DADMACs metabolism by HLM. n=4, 6, 8, 10, 12, 14.

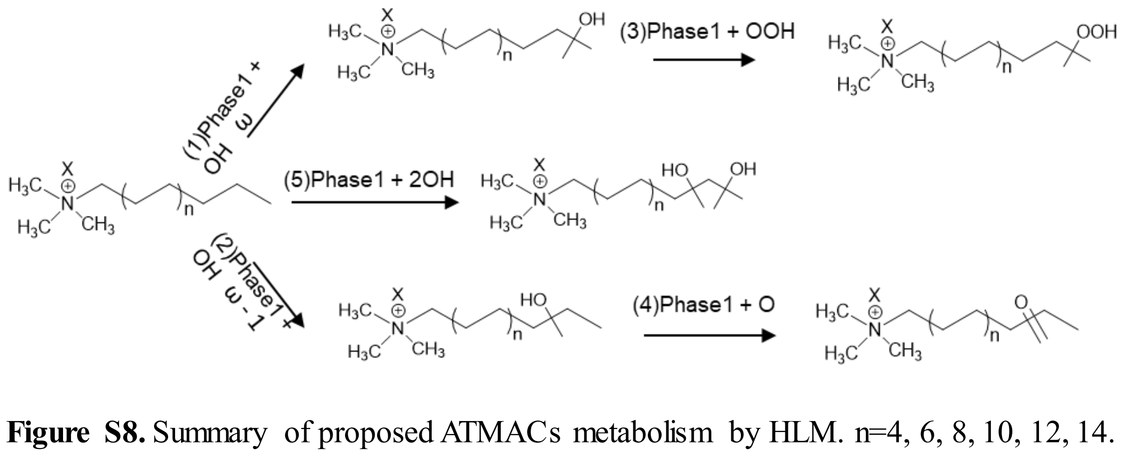

Figure S8. Summary of proposed ATMACs metabolism by HLM. n=4, 6, 8, 10, 12, 14.

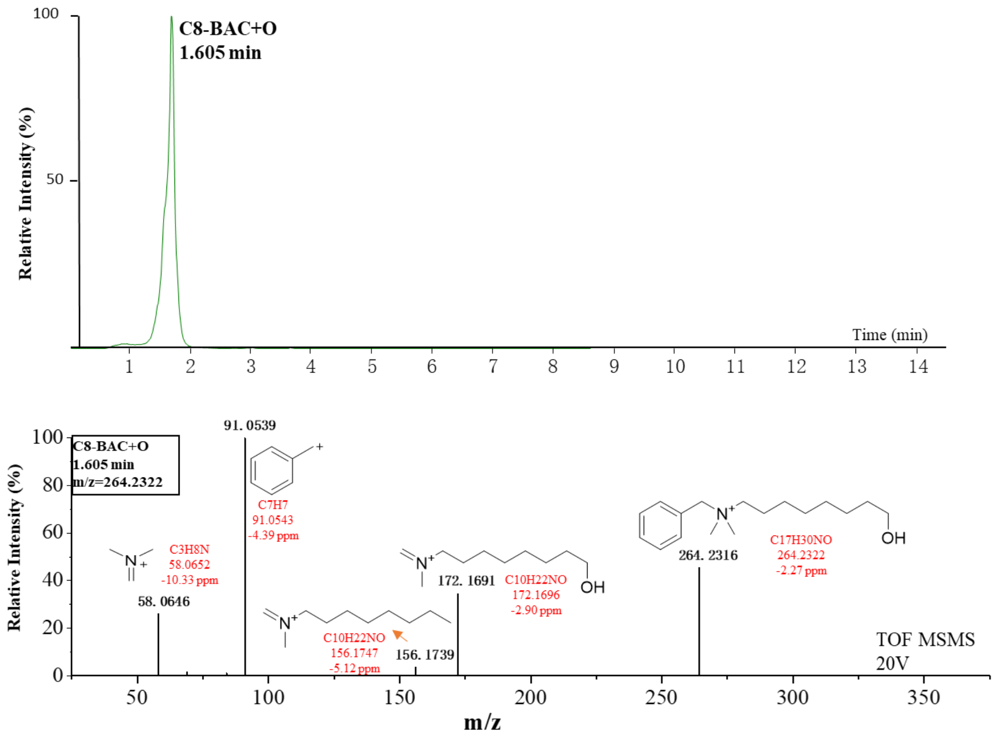

Figure S9. Chromatographic peak generated from m/z 264.2322 and MS/MS spectrum for +1O metabolite of C8-BAC.

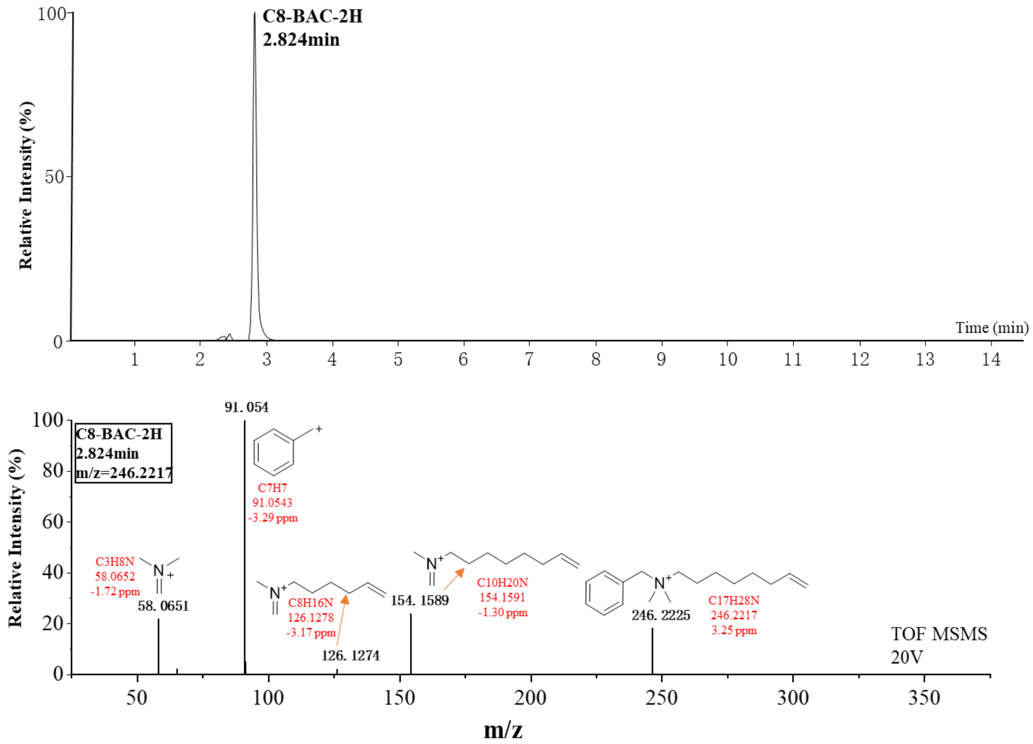

Figure S10. Chromatographic peak generated from m/z 246.2217 and MS/MS spectrum for -2H metabolite of C8-BAC.

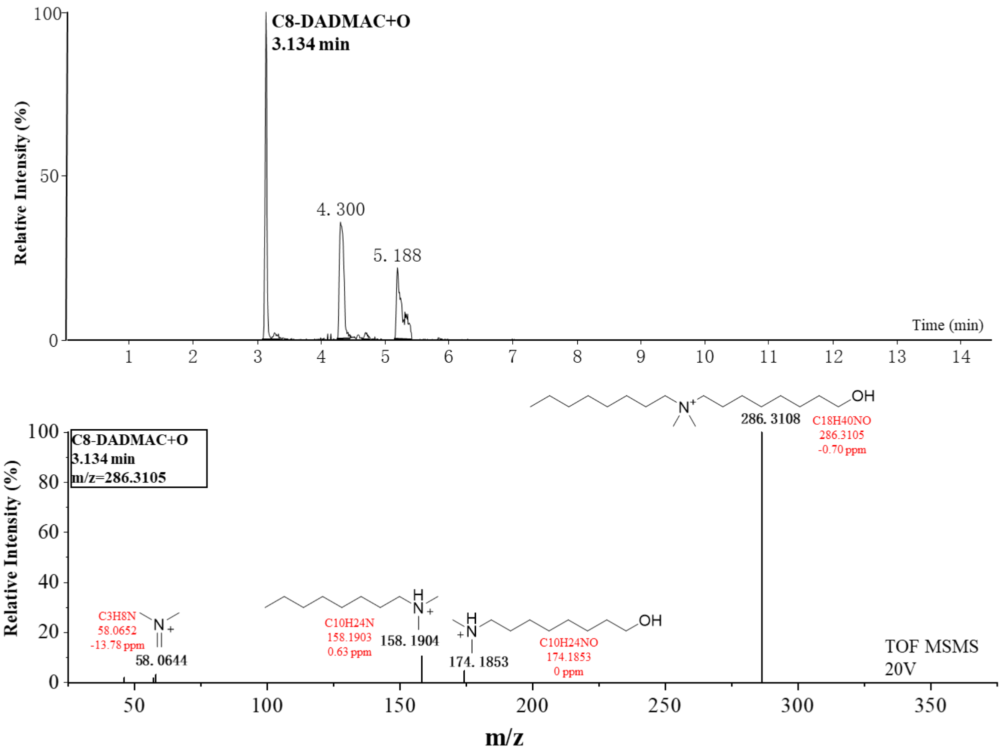

Figure S11. Chromatographic peak generated from m/z 286.3105 and MS/MS spectrum for +O metabolite of C8-DADMAC.

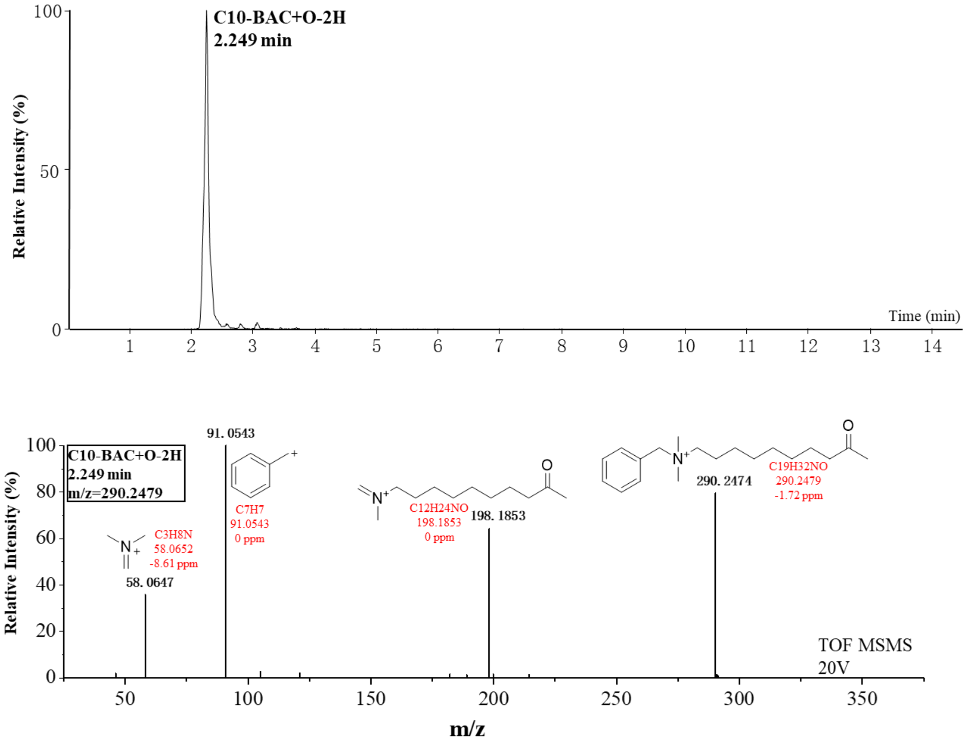

Figure S12. Chromatographic peak generated from m/z 290.2479 and MS/MS spectrum for +1O-2H metabolite of C10-BAC.

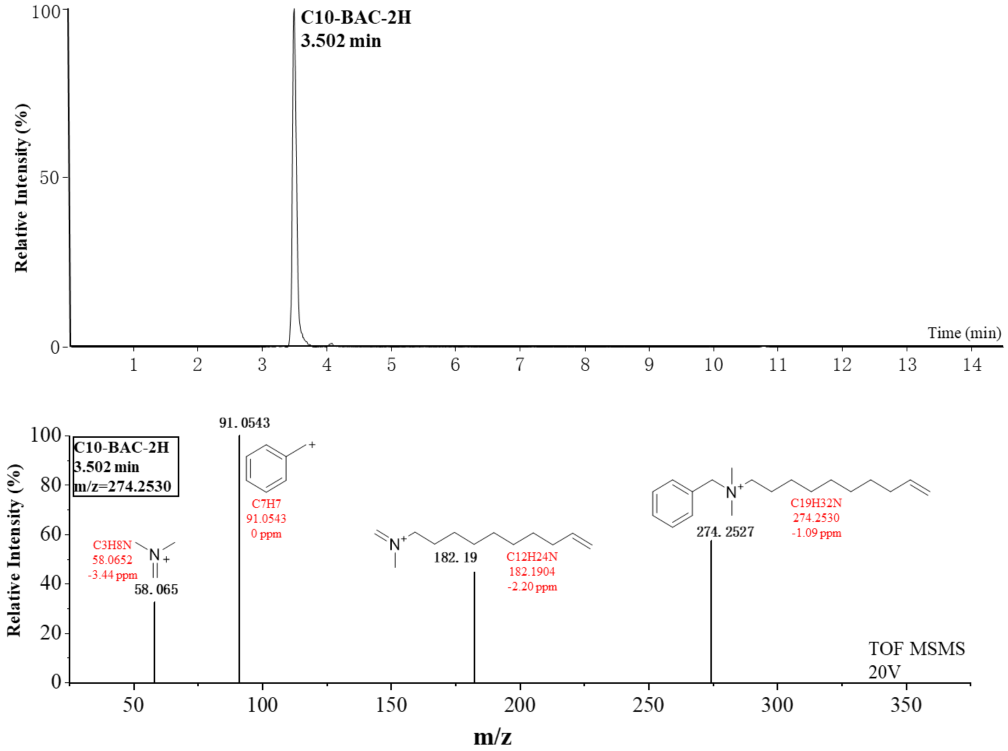

Figure S13. Chromatographic peak generated from m/z 274.2530 and MS/MS spectrum for -2H metabolite of C10-BAC.

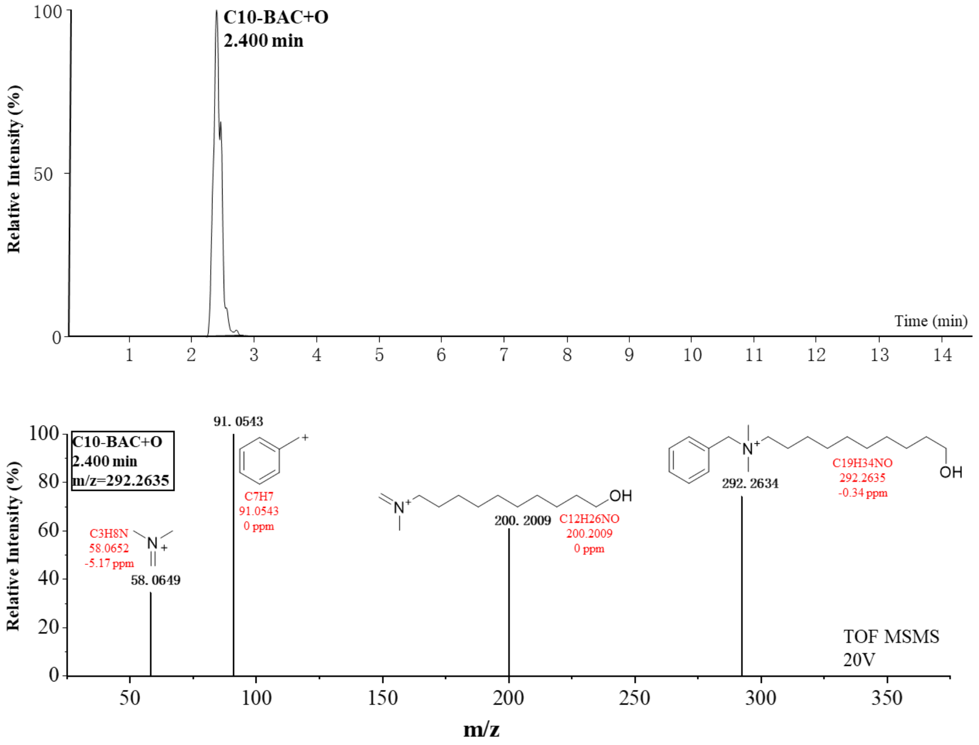

Figure S14. Chromatographic peak generated from m/z 292.2635 and MS/MS spectrum for +O metabolite of C10-BAC.

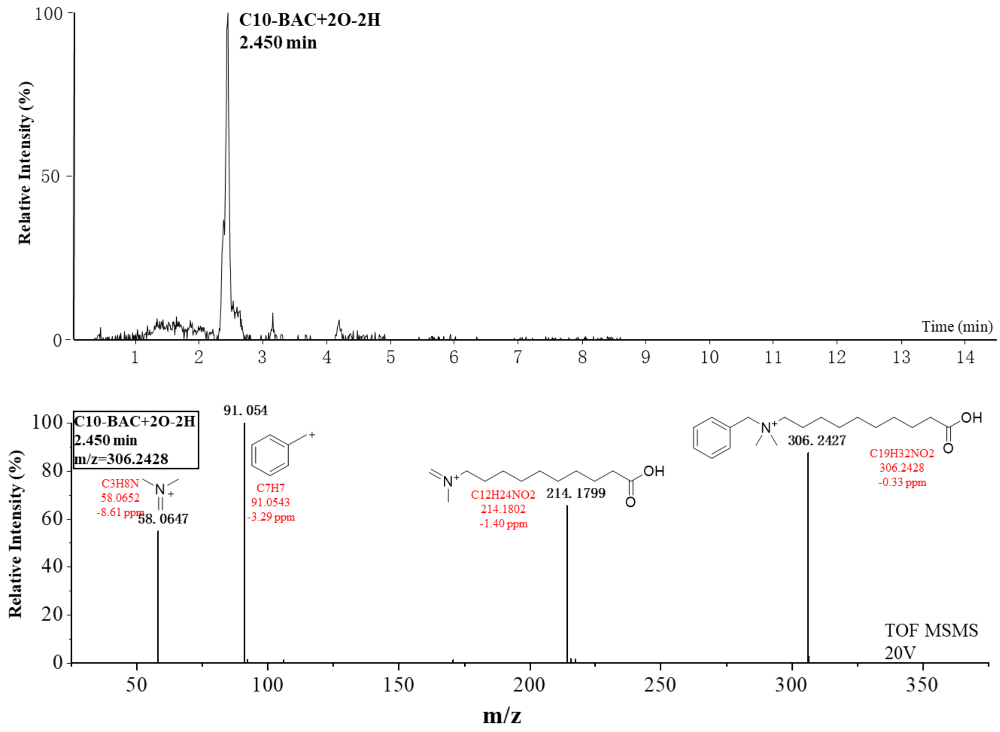

Figure S15. Chromatographic peak generated from m/z 306.2428 and MS/MS spectrum for +2O-2H metabolite of C10-BAC.

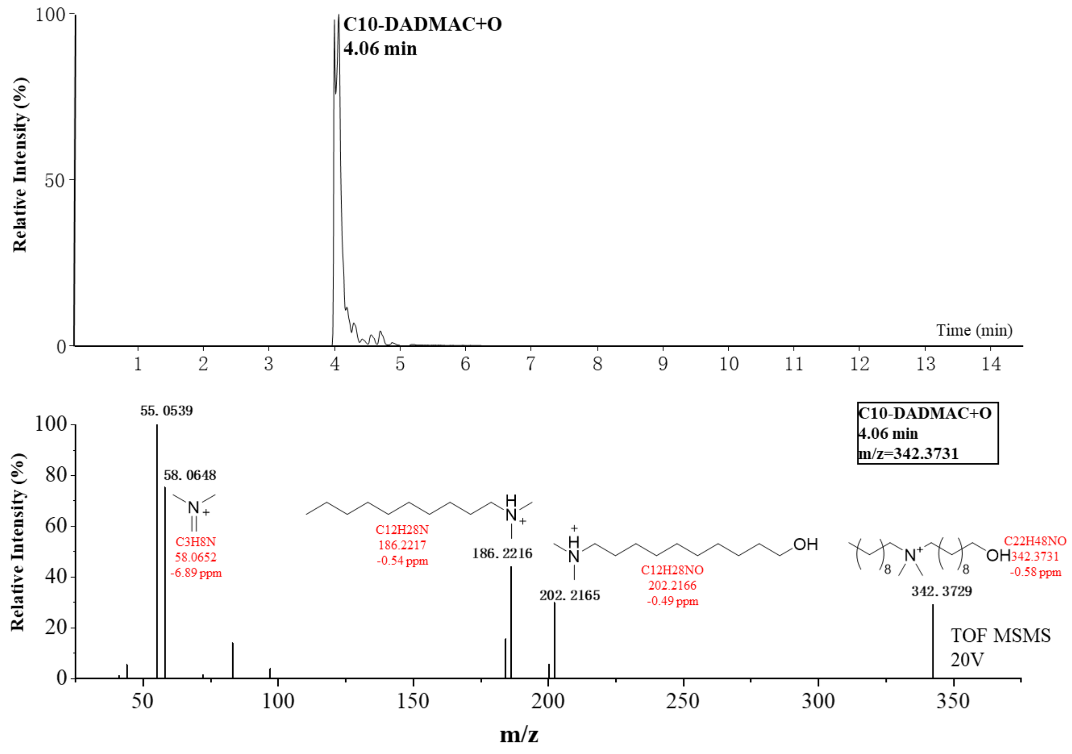

Figure S16. Chromatographic peak generated from m/z 342.3731 and MS/MS spectrum for +1O metabolite of C10-DADMAC.

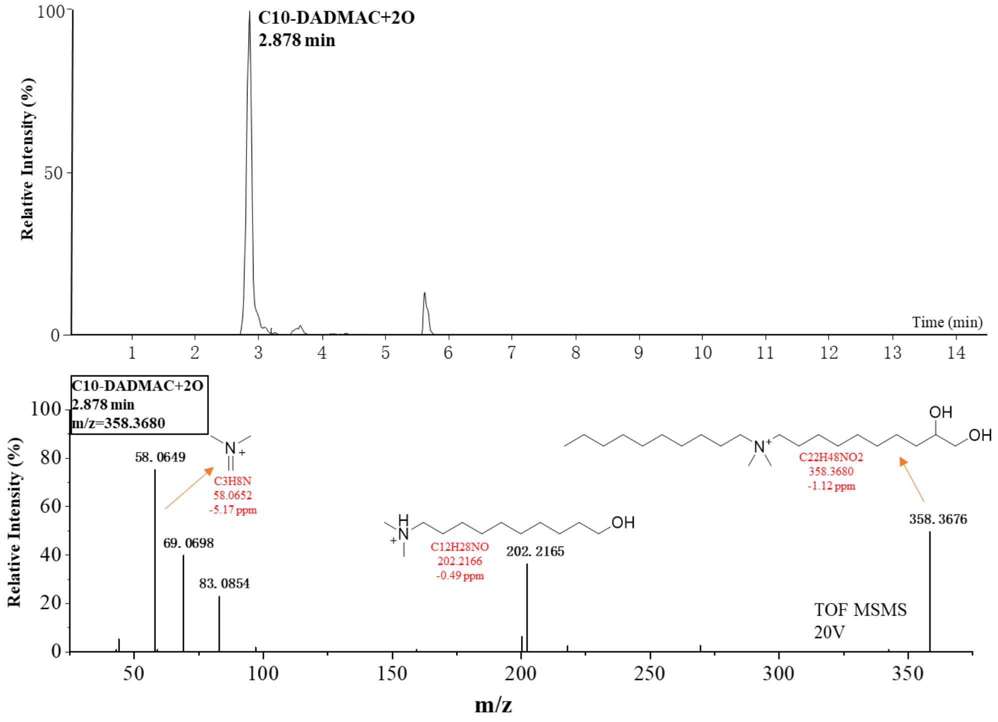

Figure S17. Chromatographic peak generated from m/z 358.3680 and MS/MS spectrum for +2O metabolite of C10-DADMAC.

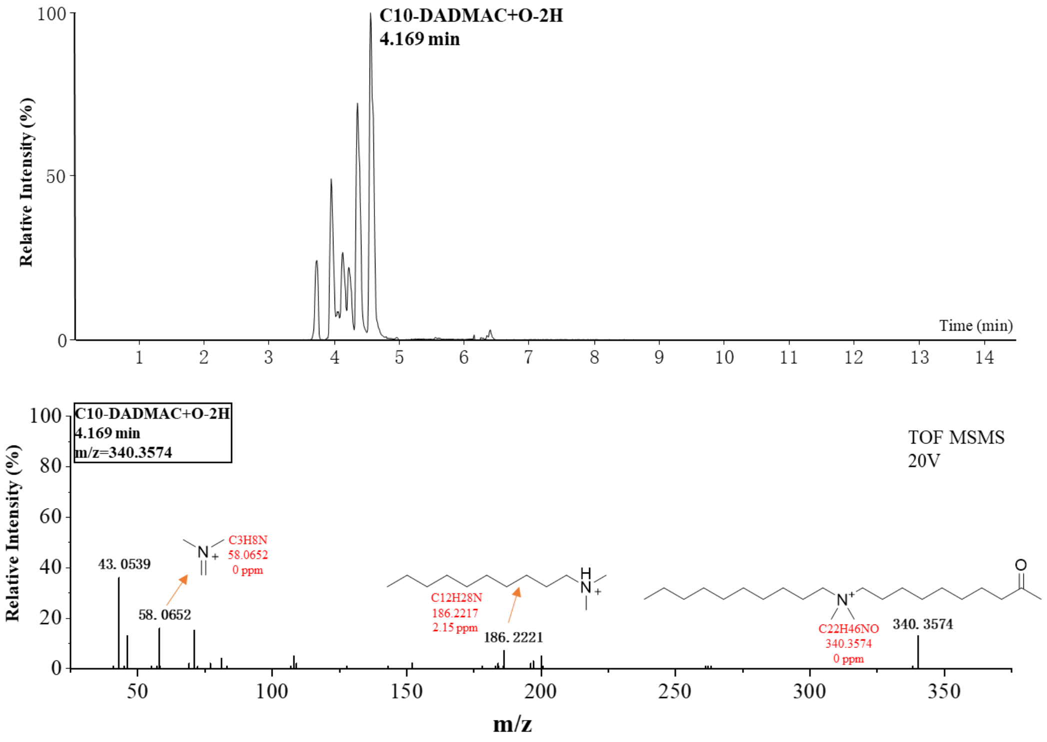

Figure S18. Chromatographic peak generated from m/z 340.3574 and MS/MS spectrum for +O-2H metabolite of C10-DADMAC.

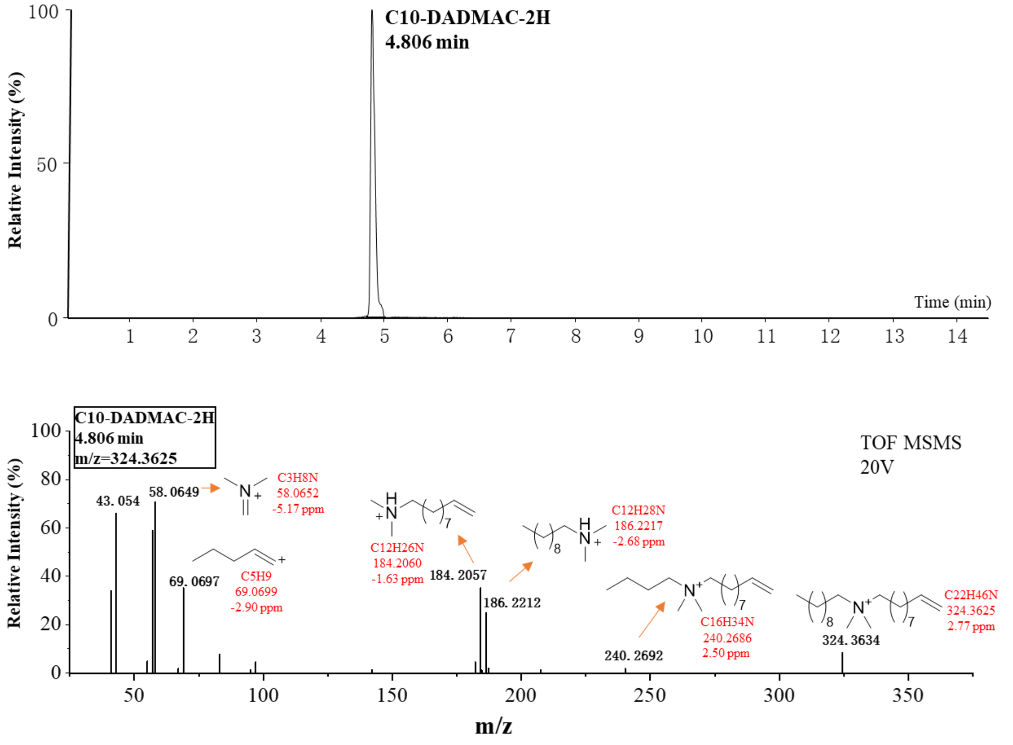

Figure S19. Chromatographic peak generated from m/z 324.3625 and MS/MS spectrum for -2H metabolite of C10-DADMAC.

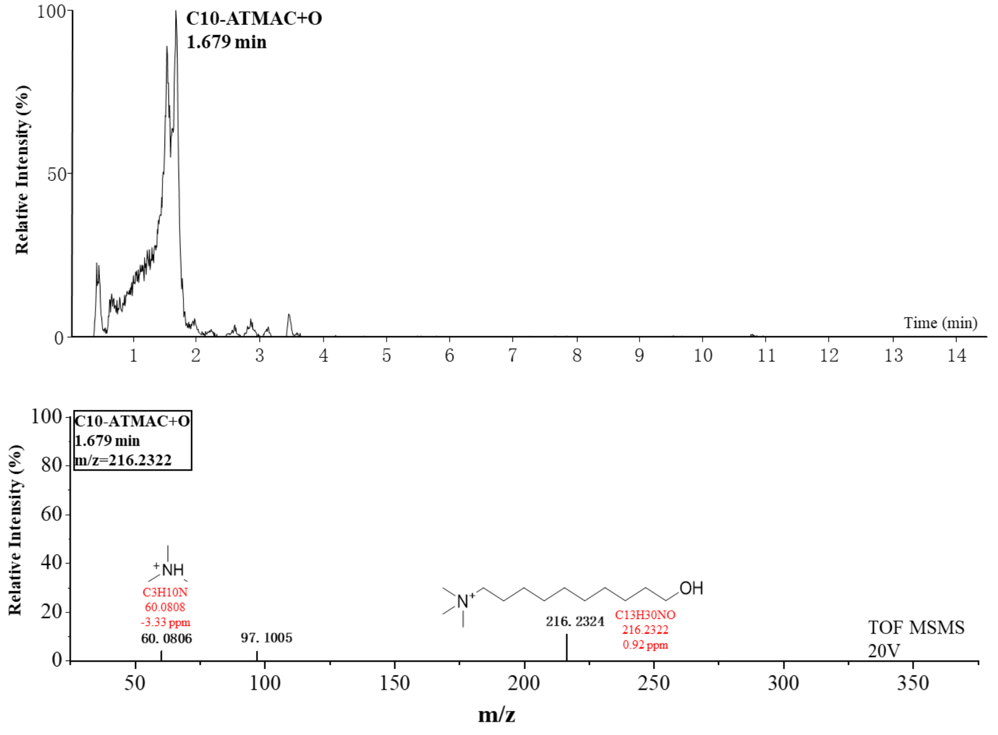

Figure S20. Chromatographic peak generated from m/z 216.2322 and MS/MS spectrum for +O metabolite of C10-ATMAC.

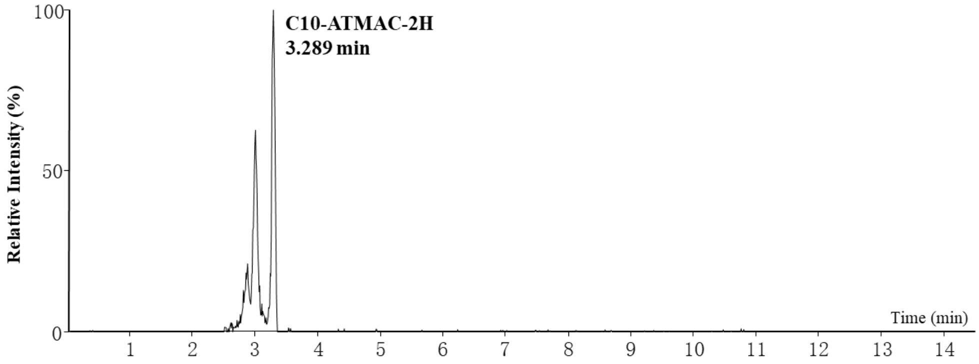

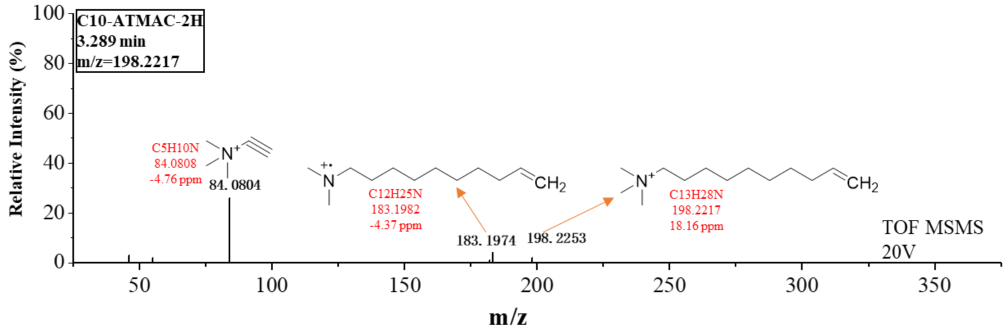

Figure S21. Chromatographic peak generated from m/z 198.2217 and MS/MS spectrum for -2H metabolite of C10-ATMAC.

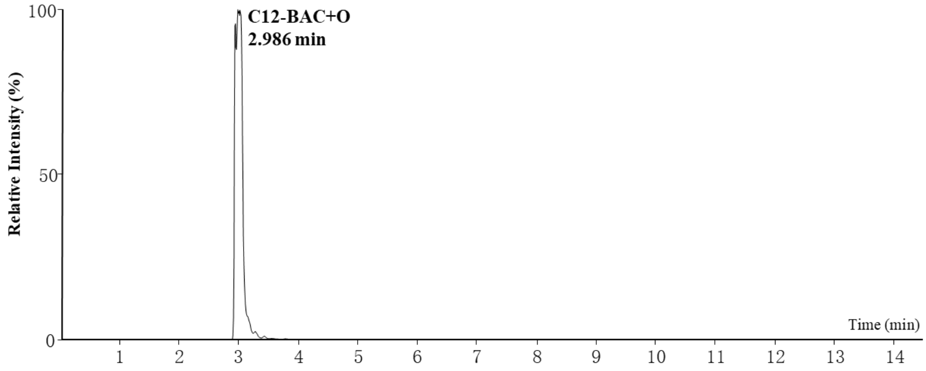

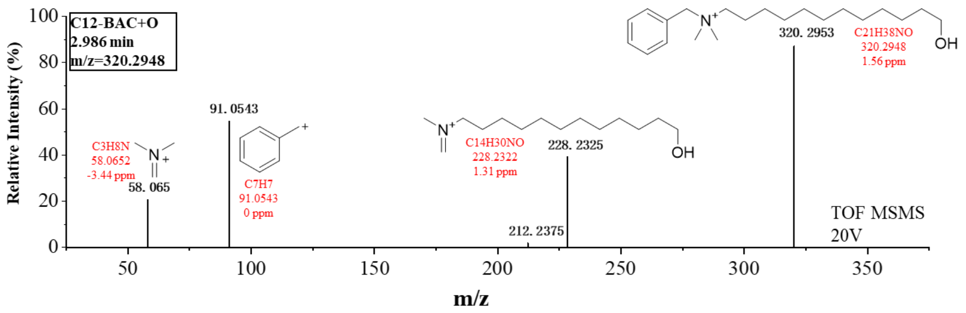

Figure S22. Chromatographic peak generated from m/z 320.2948 and MS/MS spectrum for +O metabolite of C12-BAC.

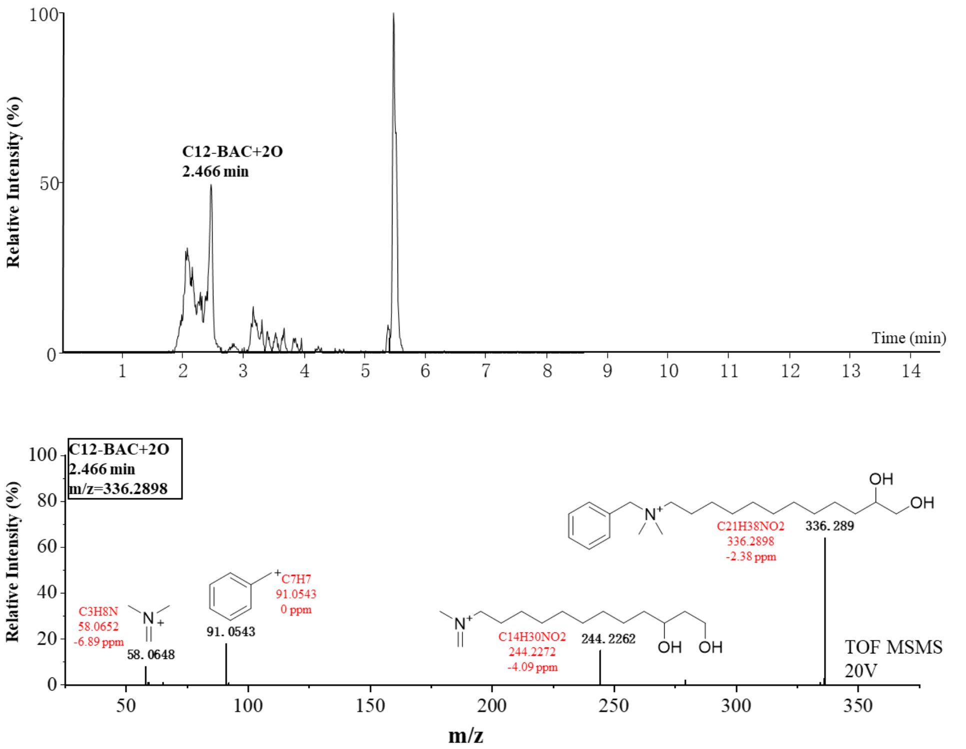

Figure S23. Chromatographic peak generated from m/z 336.2898 and MS/MS spectrum for +2O metabolite of C12-BAC.

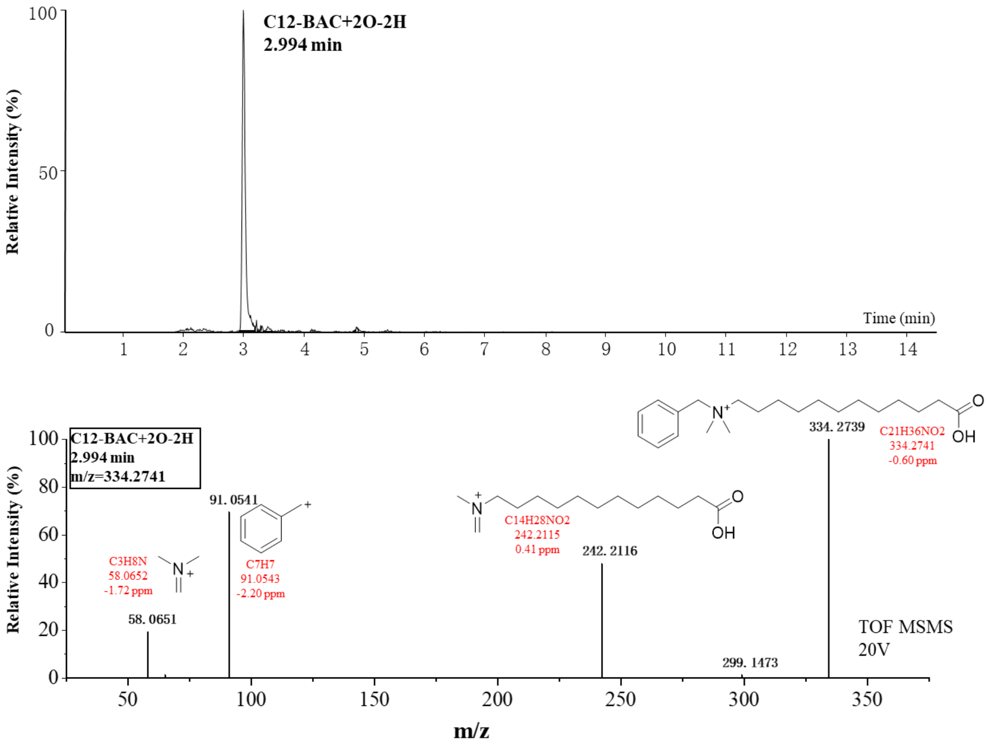

Figure S24. Chromatographic peak generated from m/z 334.2741 and MS/MS spectrum for +2O-2H metabolite of C12-BAC.

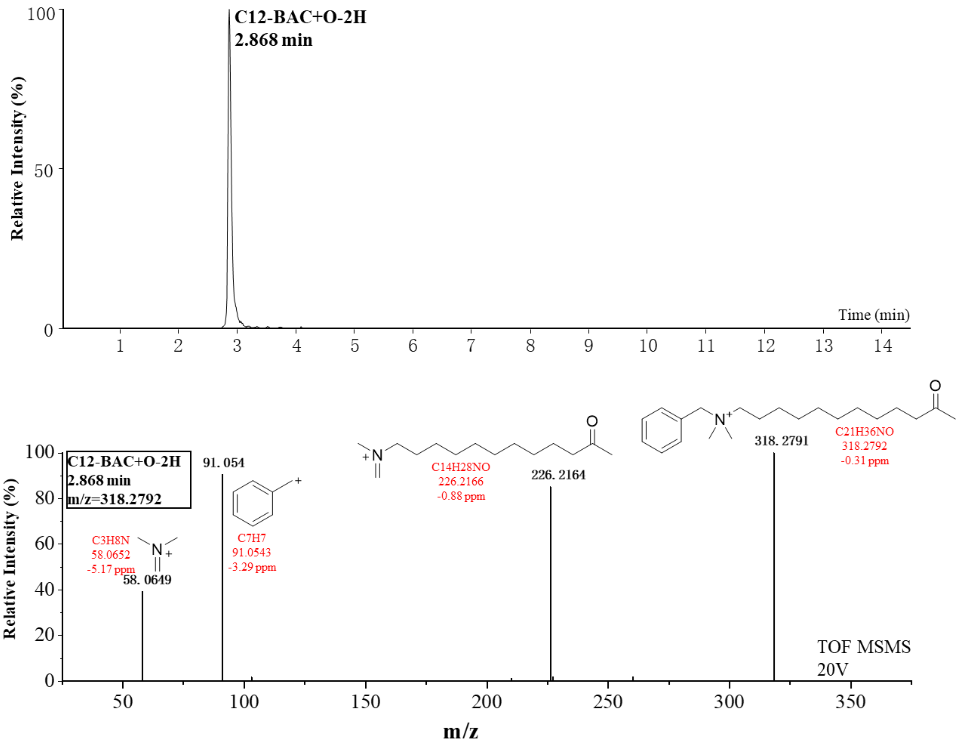

Figure S25. Chromatographic peak generated from m/z 318.2792 and MS/MS spectrum for +O-2H metabolite of C12-BAC.

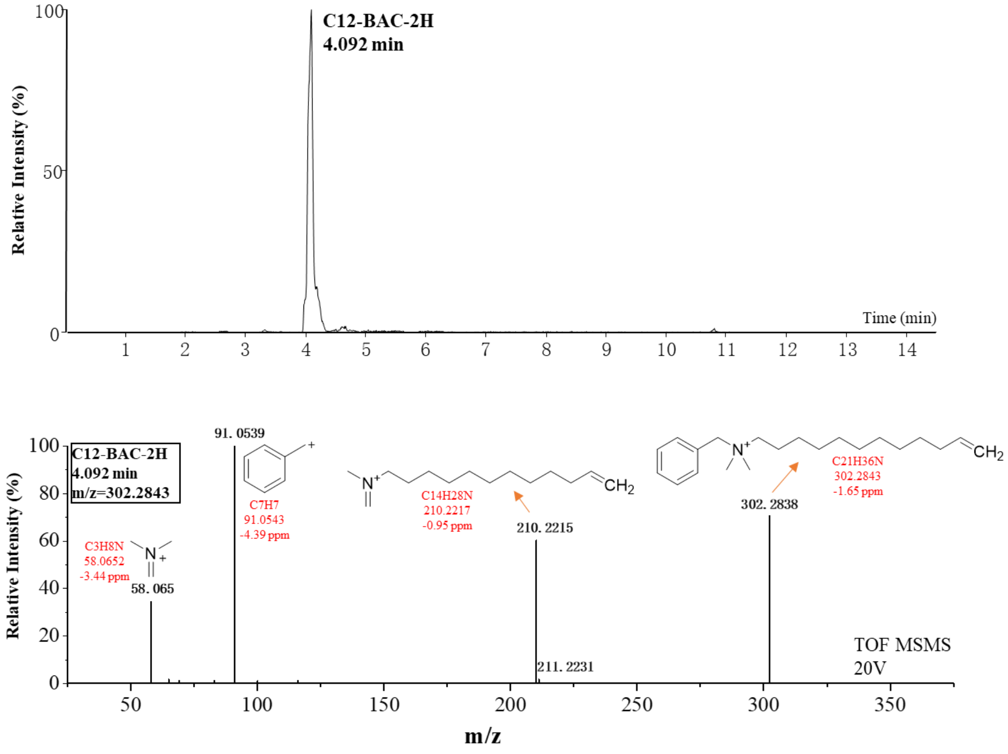

Figure S26. Chromatographic peak generated from m/z 302.2843 and MS/MS spectrum for -2H metabolite of C12-BAC.

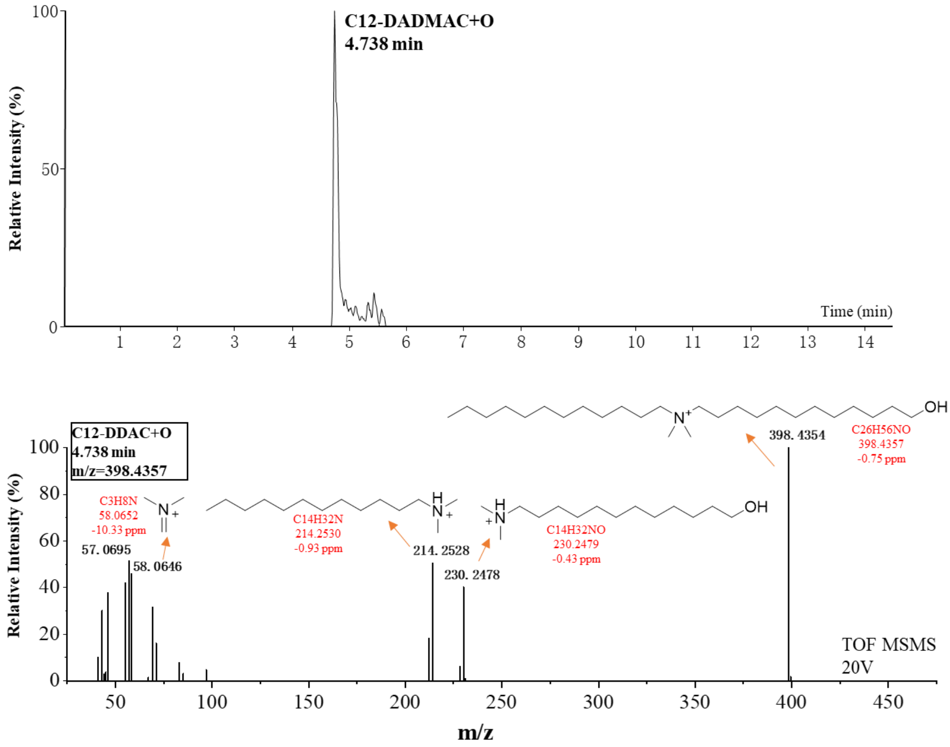

Figure S27. Chromatographic peak generated from m/z 398.4357 and MS/MS spectrum for +O metabolite of C12-DADMAC.

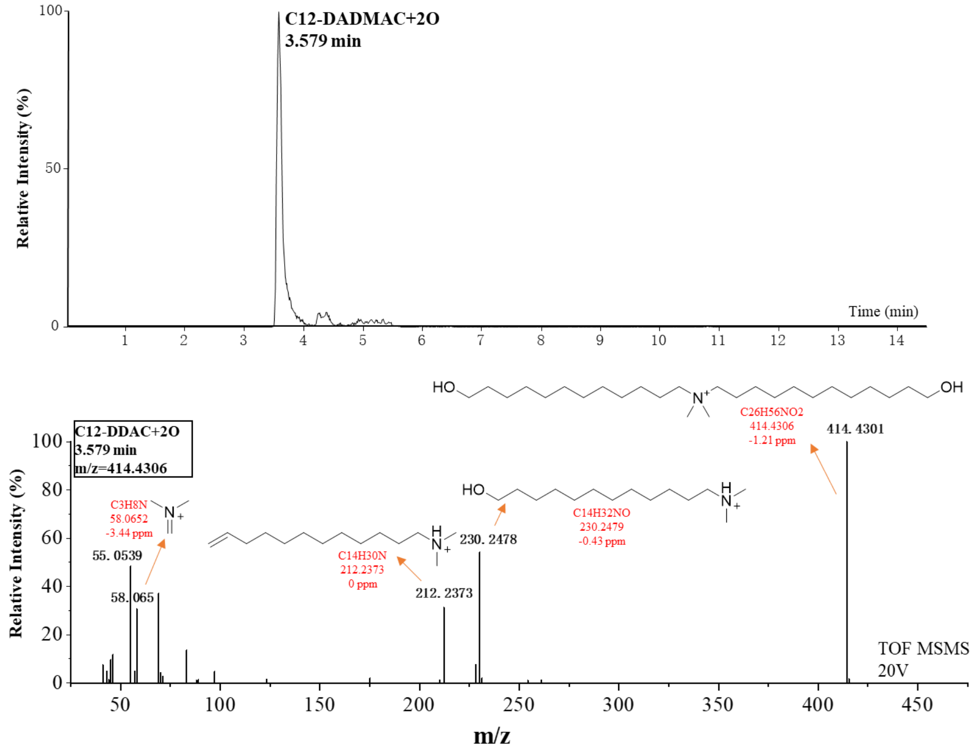

Figure S28. Chromatographic peak generated from m/z 414.4306 and MS/MS spectrum for +2O metabolite of C12-DADMAC.

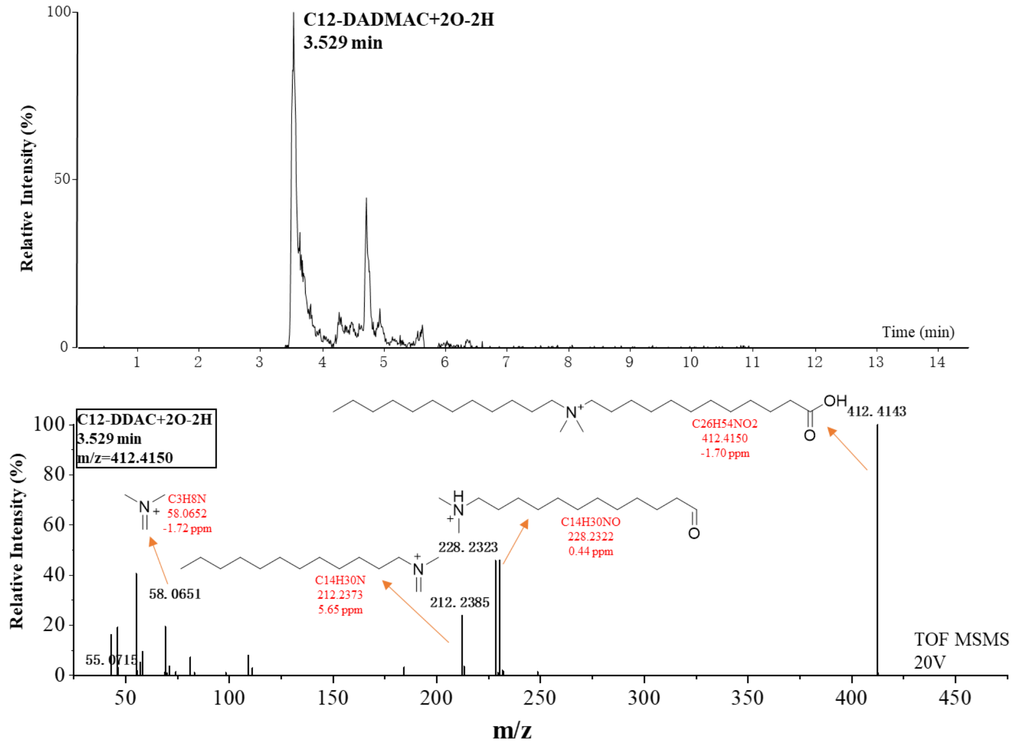

Figure S29 Chromatographic peak generated from m/z 412.4150 and MS/MS spectrum for +2O-2H metabolite of C12-DADMAC.

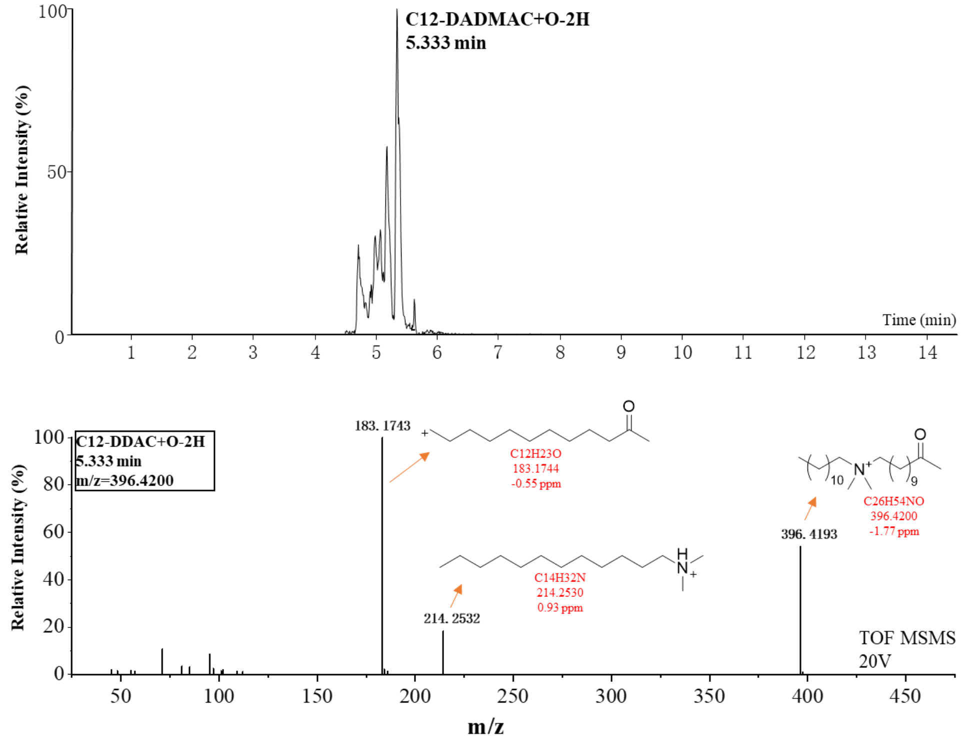

Figure S30. Chromatographic peak generated from m/z 396.4200 and MS/MS spectrum for +O-2H metabolite of C12-DADMAC.

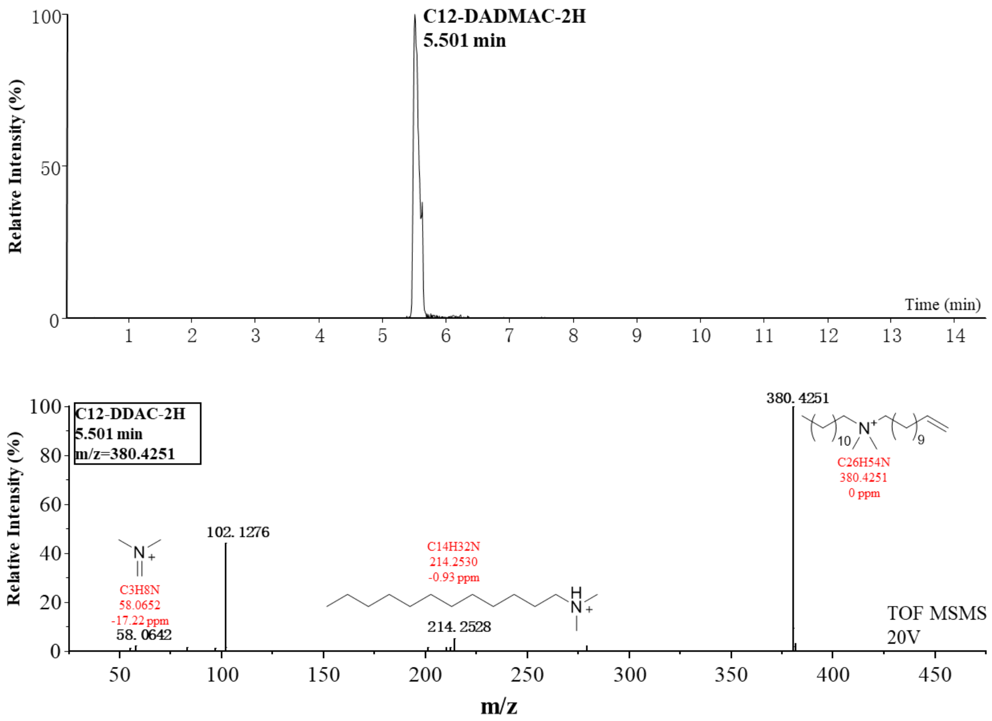

Figure S31. Chromatographic peak generated from m/z 380.4251 and MS/MS spectrum for -2H metabolite of C12-DADMAC.

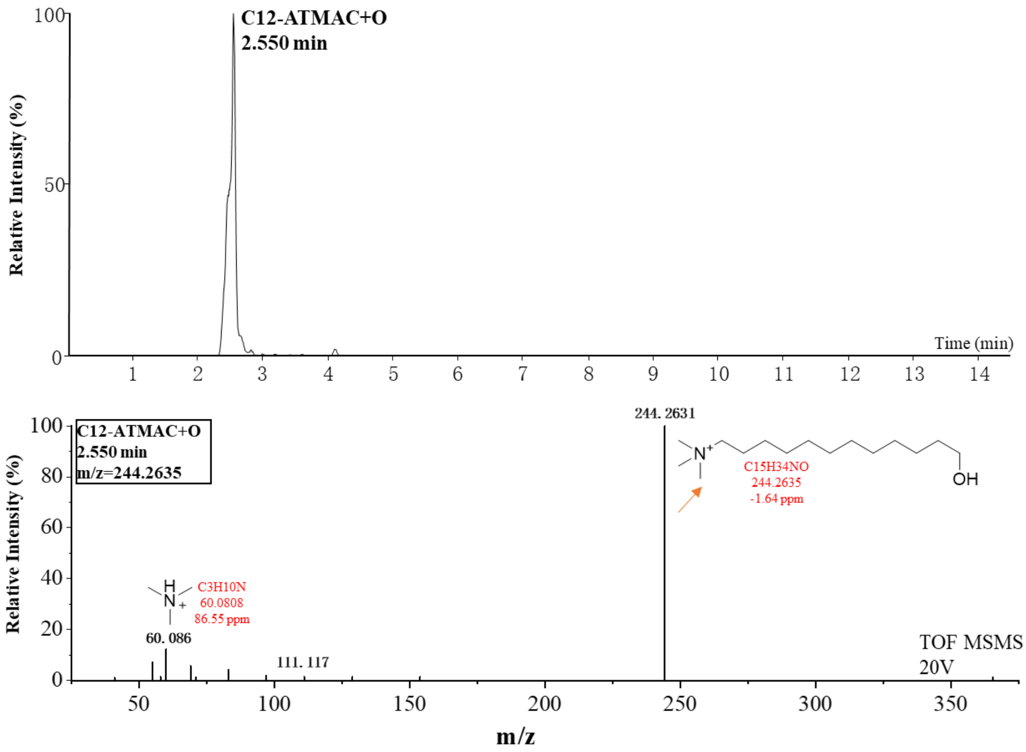

Figure S32. Chromatographic peak generated from m/z 244.2635 and MS/MS spectrum for +O metabolite of C12-ATMAC.

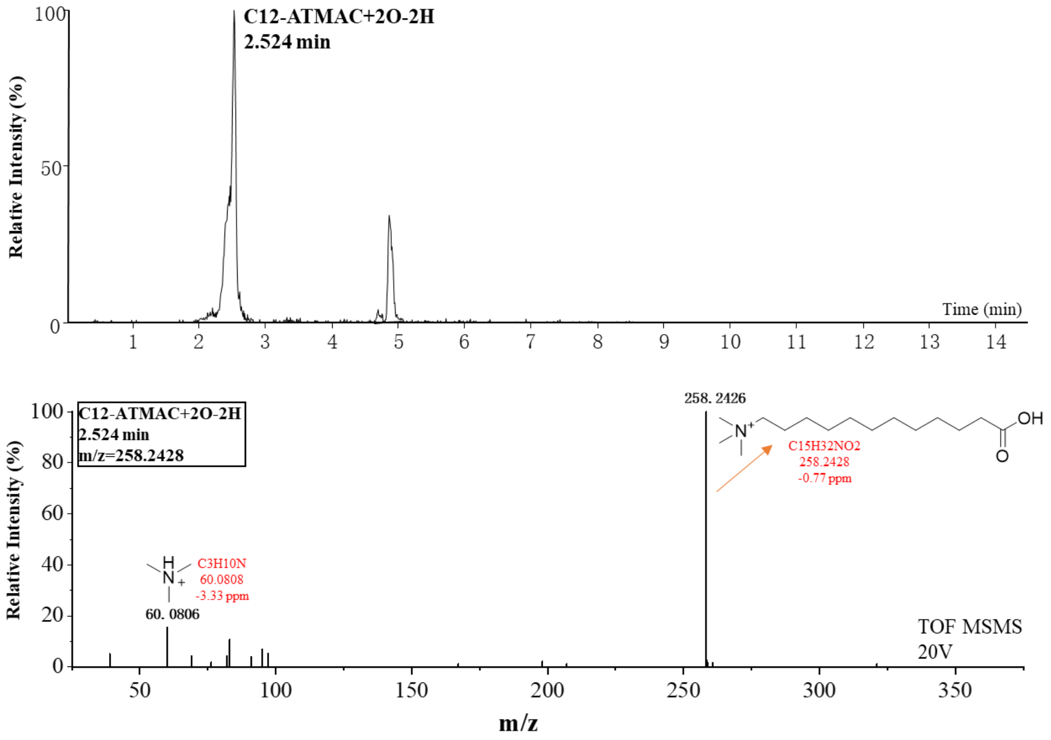

Figure S33. Chromatographic peak generated from m/z 258.2428 and MS/MS spectrum for +2O-2H metabolite of C12-ATMAC.

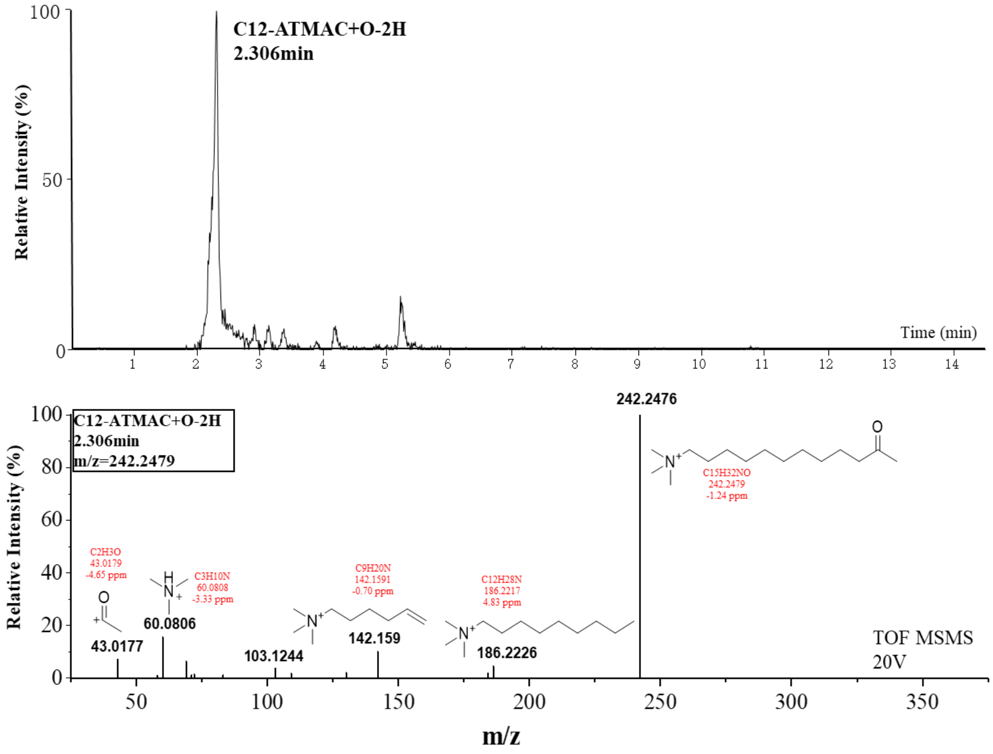

Figure S34. Chromatographic peak generated from m/z 242.2479 and MS/MS spectrum for +O-2H metabolite of C12-ATMAC.

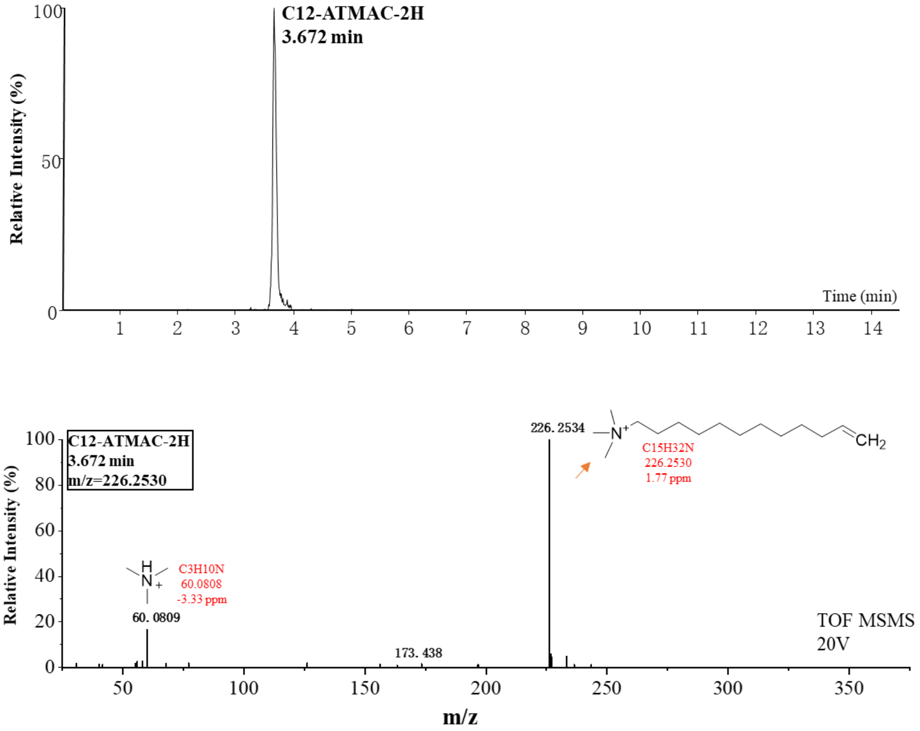

Figure S35. Chromatographic peak generated from m/z 226.2530 and MS/MS spectrum for -2H metabolite of C12-ATMAC.

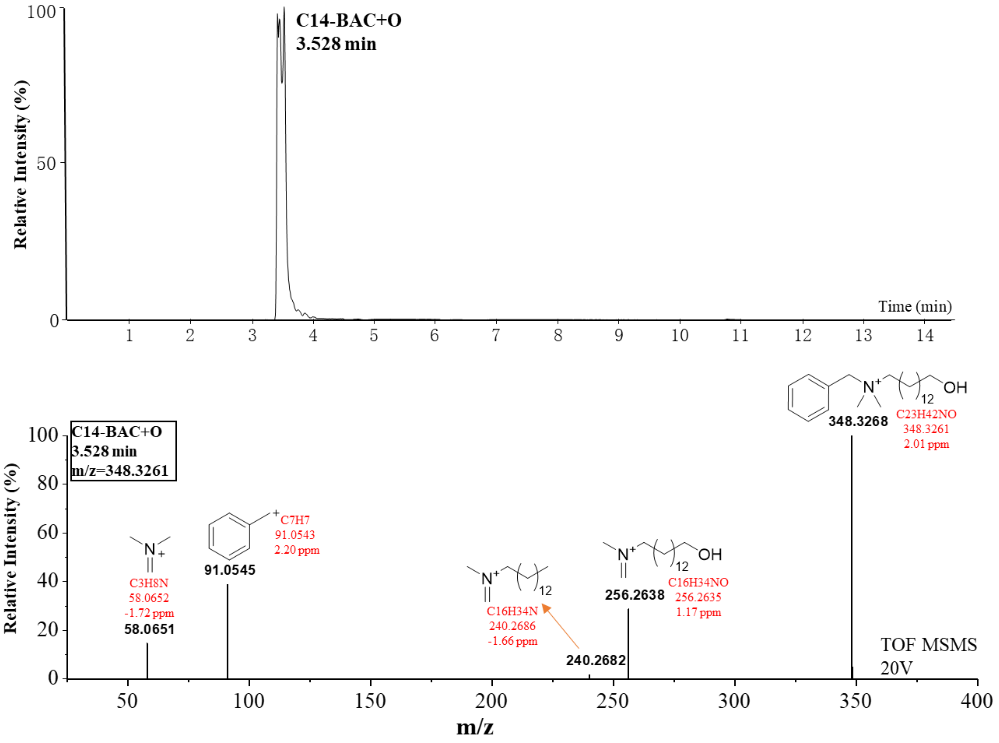

Figure S36. Chromatographic peak generated from m/z 348.3261 and MS/MS spectrum for +O metabolite of C14-BAC.

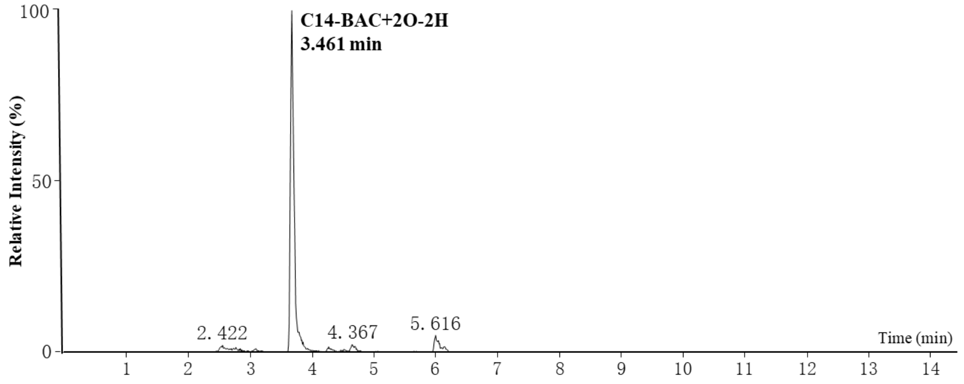

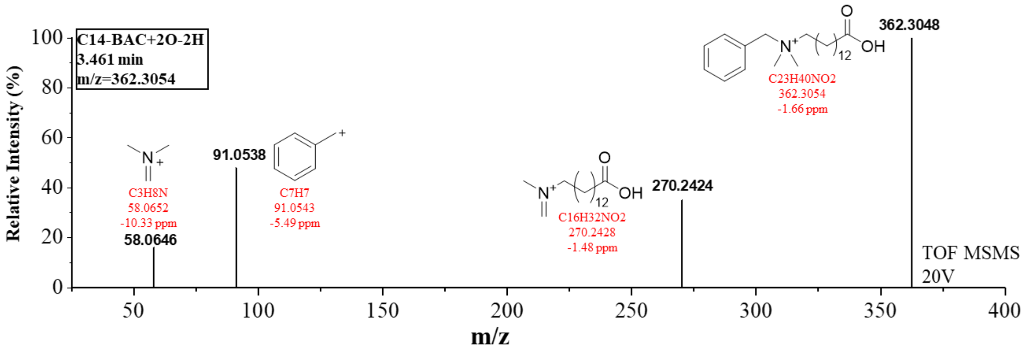

Figure S37. Chromatographic peak generated from m/z 362.3054 and MS/MS spectrum for +2O-2H metabolite of C14-BAC.

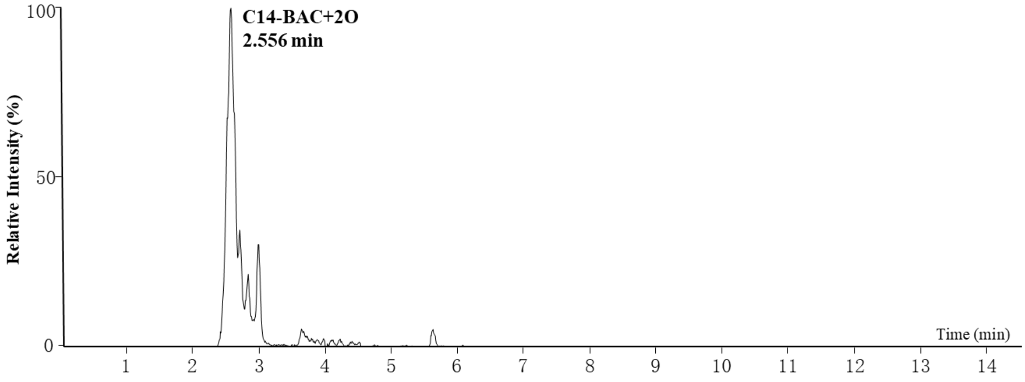

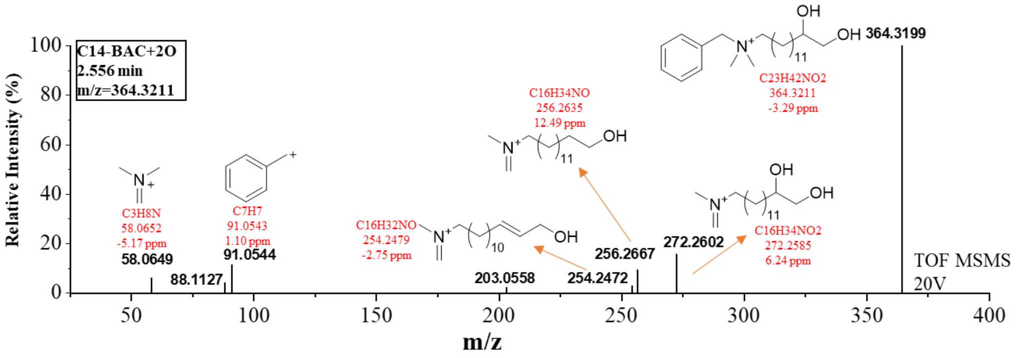

Figure S38. Chromatographic peak generated from m/z 364.3211 and MS/MS spectrum for +2O metabolite of C14-BAC.

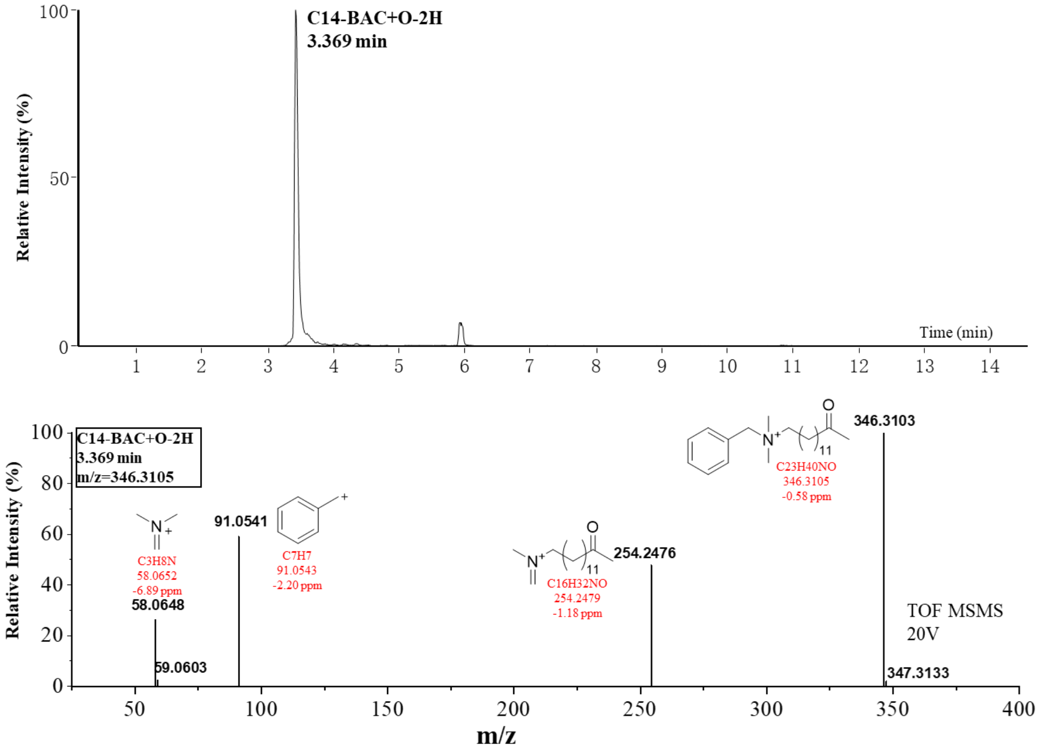

Figure S39. Chromatographic peak generated from m/z 346.3105 and MS/MS spectrum for +O-2H metabolite of C14-BAC.

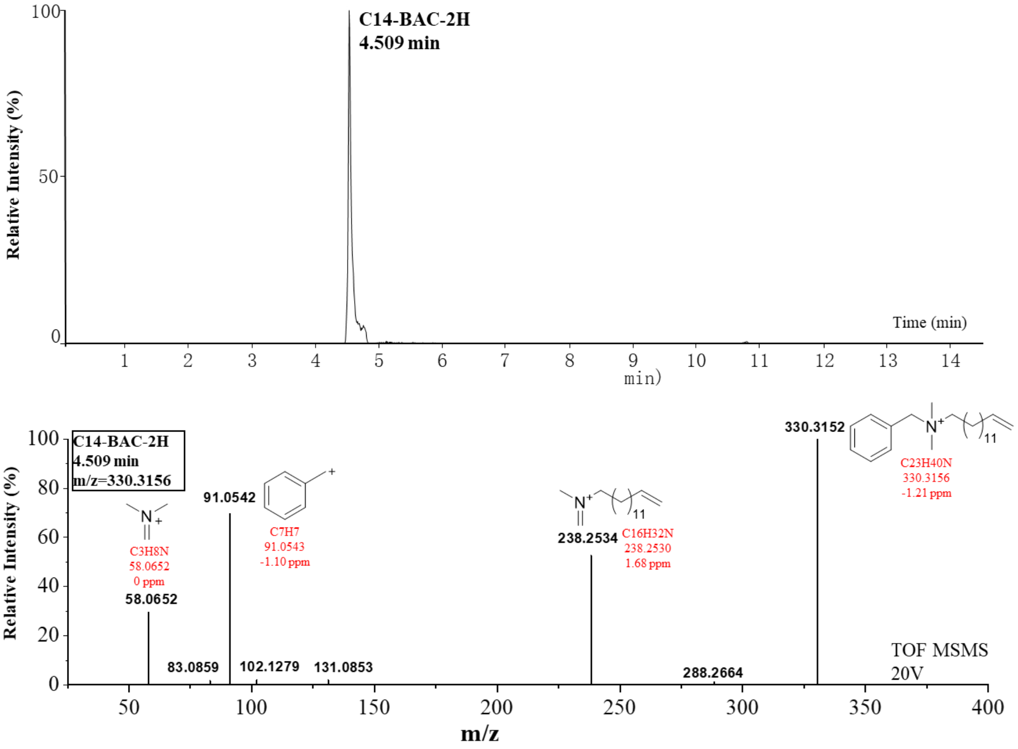

Figure S40. Chromatographic peak generated from m/z 330.3156 and MS/MS spectrum for -2H metabolite of C14-BAC.

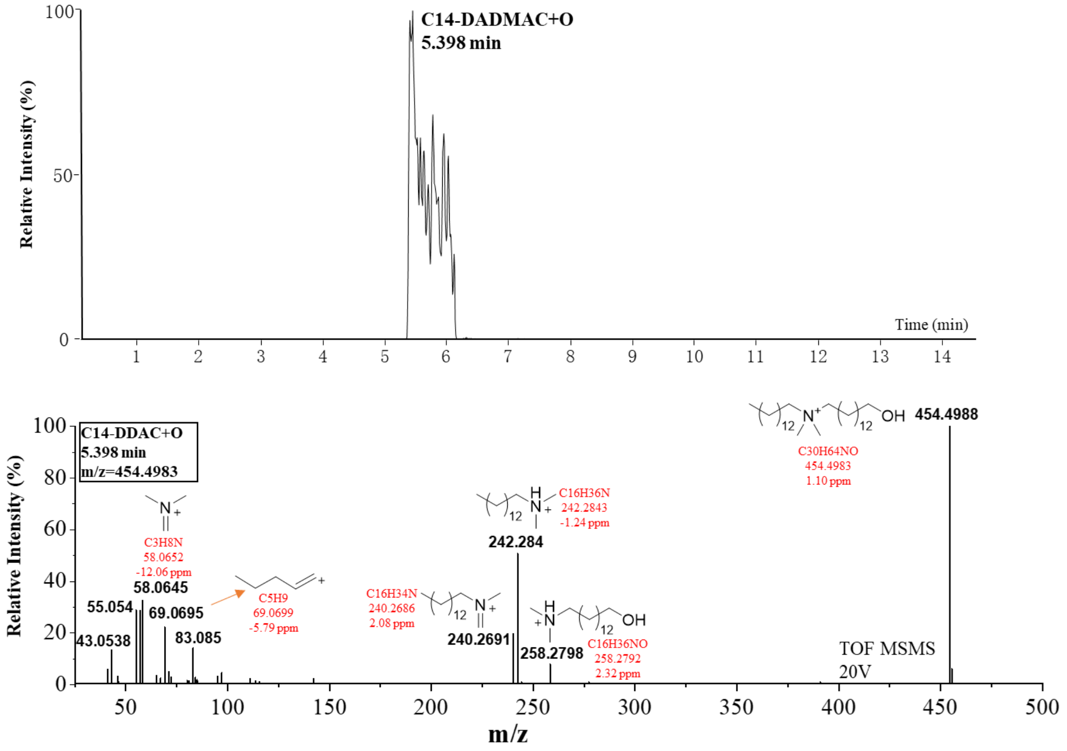

Figure S41. Chromatographic peak generated from m/z 454.4983 and MS/MS spectrum for +O metabolite of C14-DADMAC.

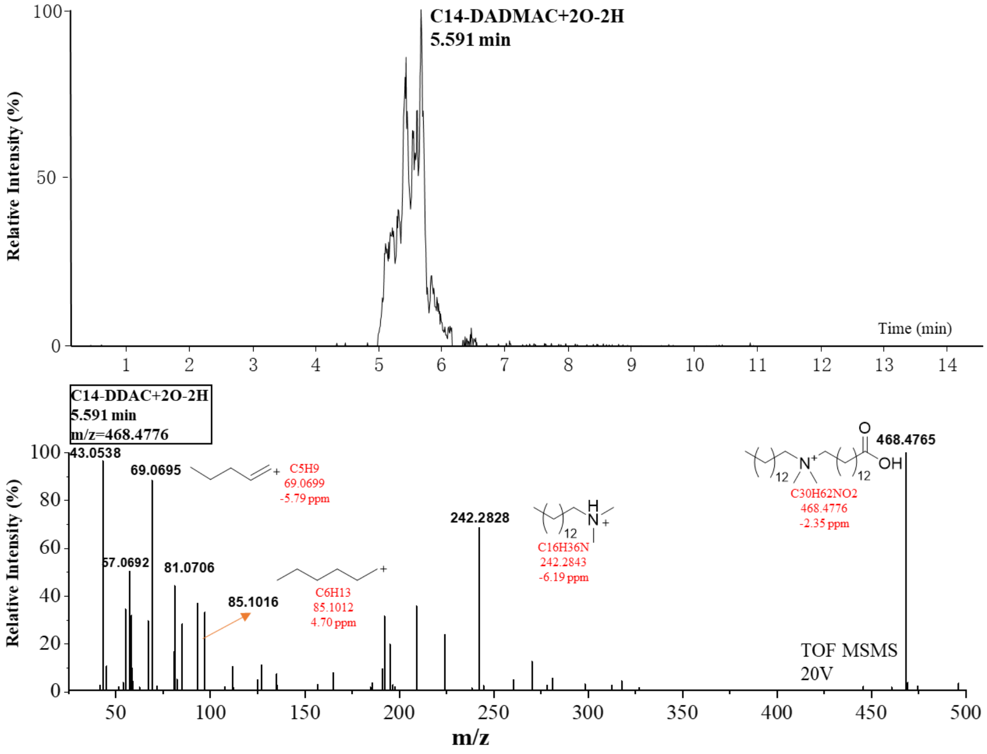

Figure S42. Chromatographic peak generated from m/z 468.4776 and MS/MS spectrum for +2O-2H metabolite of C14-DADMAC.

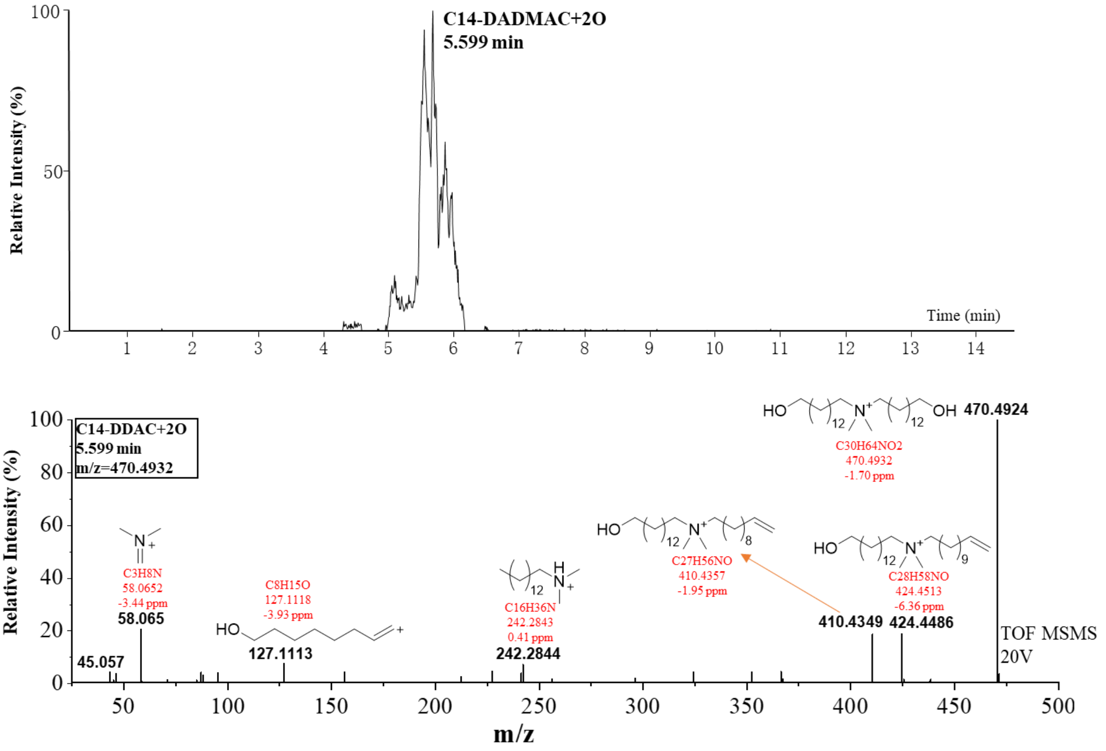

Figure S43. Chromatographic peak generated from m/z 470.4932 and MS/MS spectrum for +2O metabolite of C14-DADMAC.

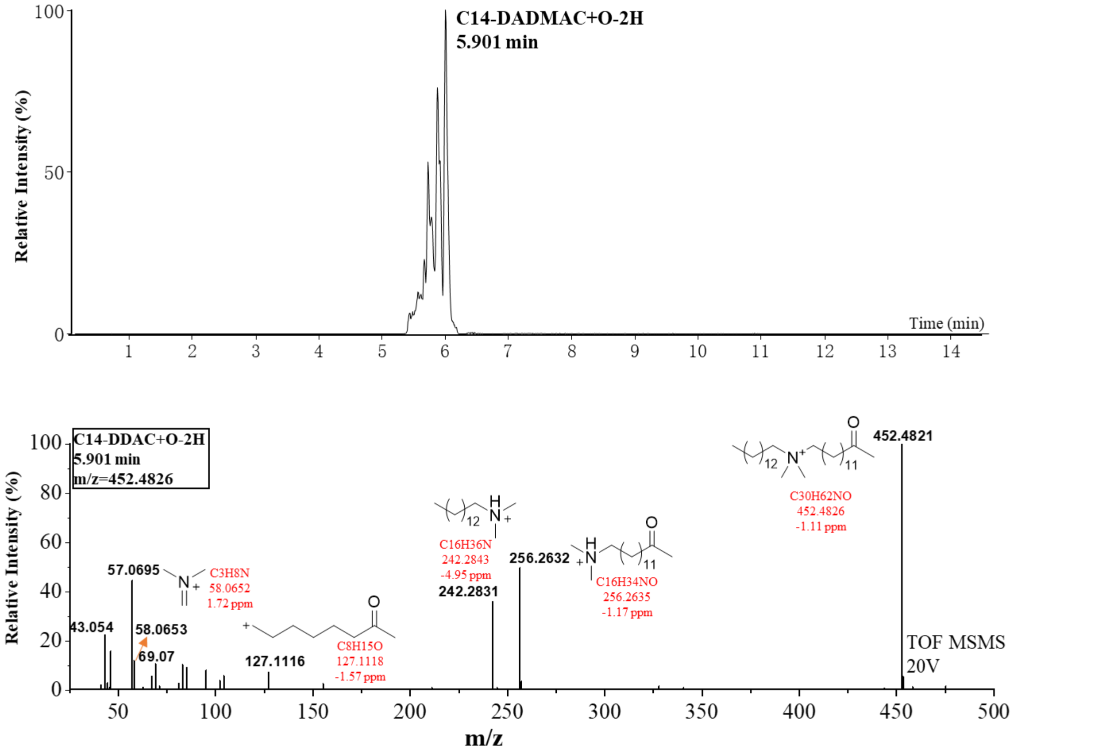

Figure S44. Chromatographic peak generated from m/z 452.4826 and MS/MS spectrum for +O-2H metabolite of C14-DADMAC.

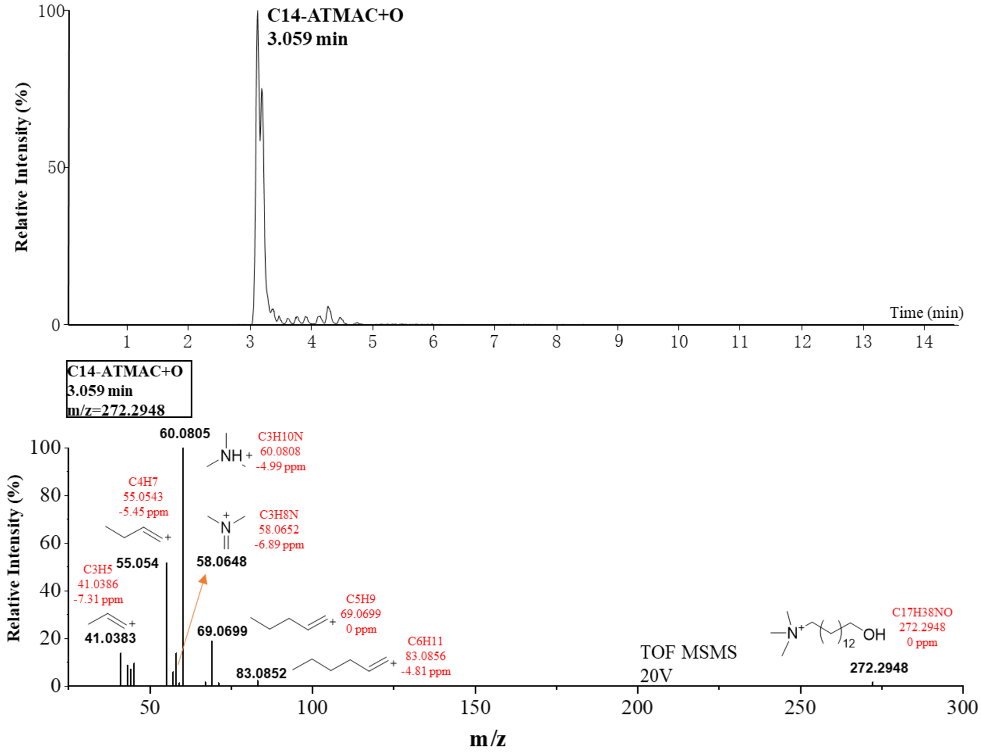

Figure S45. Chromatographic peak generated from m/z 272.2948 and MS/MS spectrum for +O metabolite of C14-ATMAC.

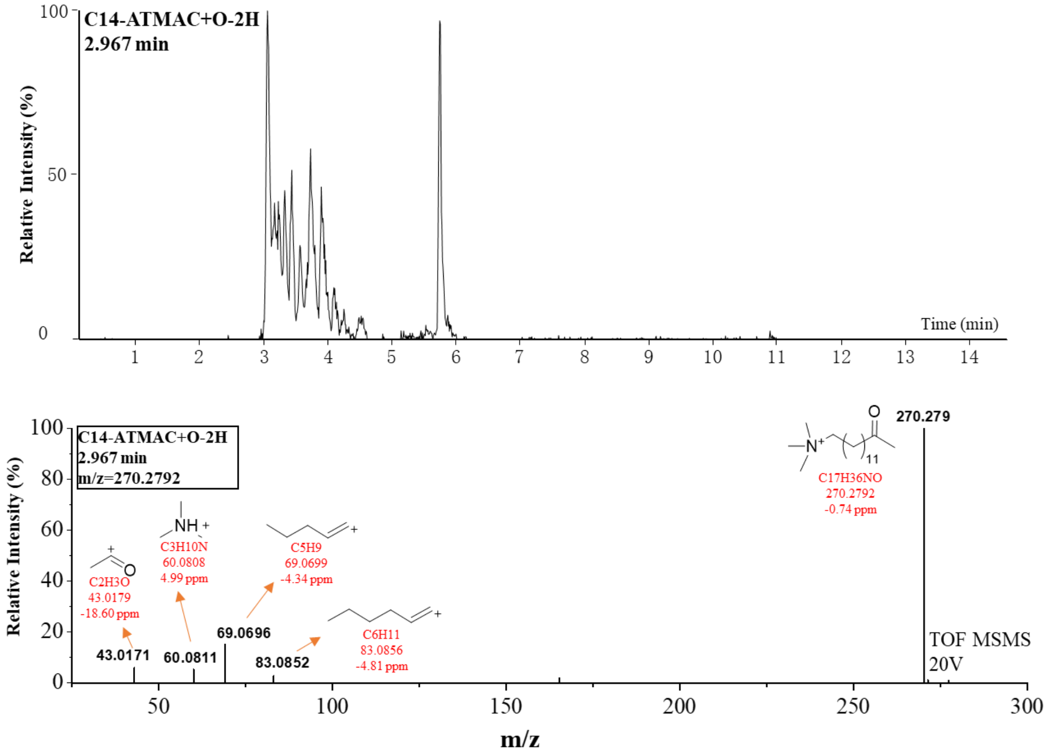

Figure S46. Chromatographic peak generated from m/z 270.2792 and MS/MS spectrum for +O-2H metabolite of C14-ATMAC.

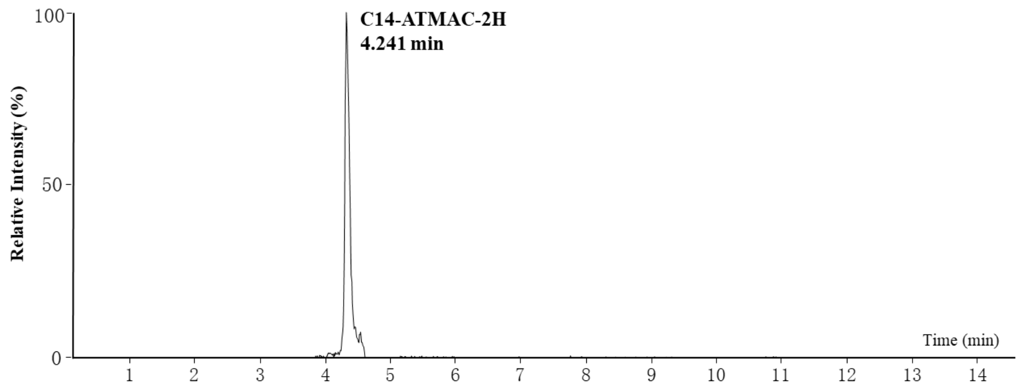

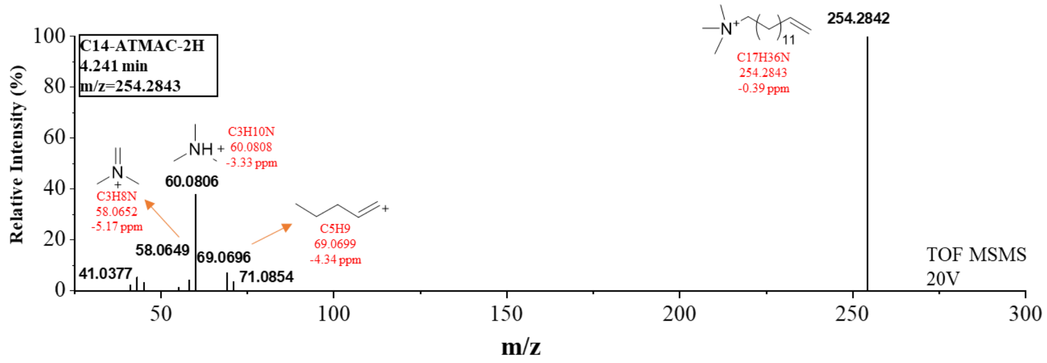

Figure S47. Chromatographic peak generated from m/z 254.2843 and MS/MS spectrum for -2H metabolite of C14-ATMAC.

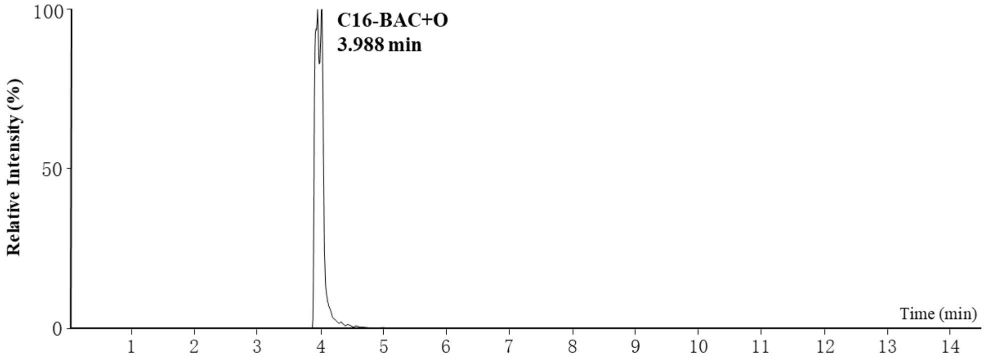

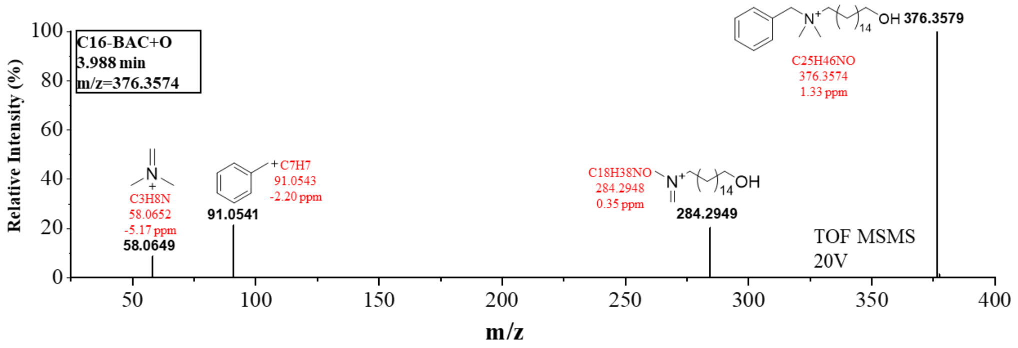

Figure S48. Chromatographic peak generated from m/z 376.3574 and MS/MS spectrum for +O metabolite of C16-BAC.

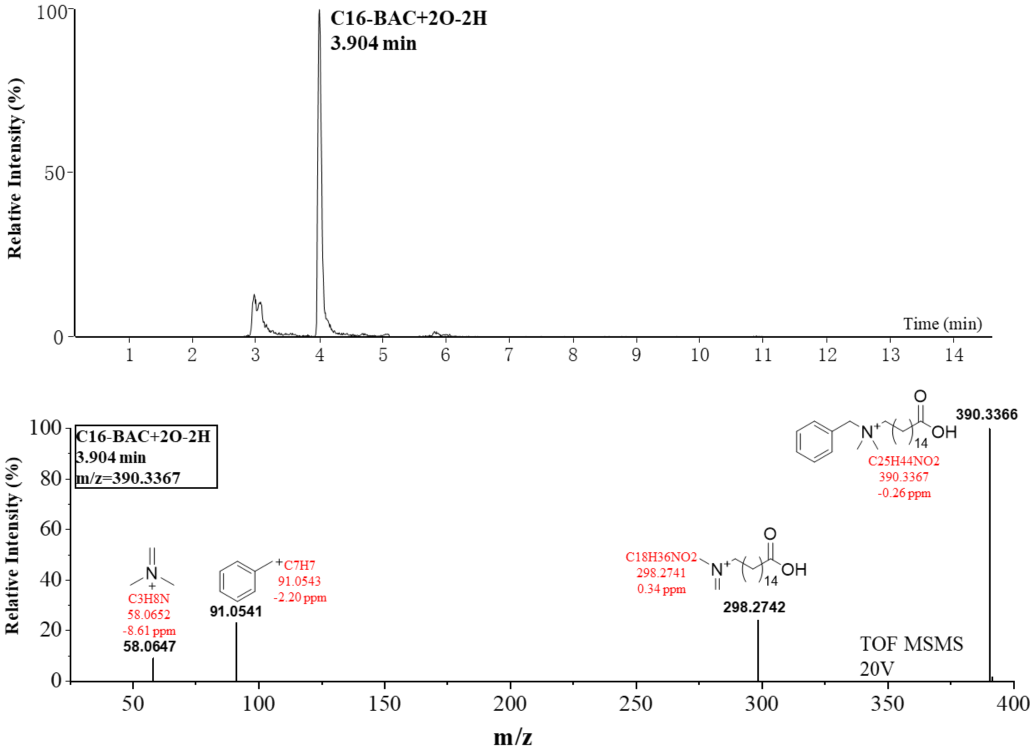

Figure S49. Chromatographic peak generated from m/z 390.3367 and MS/MS spectrum for +2O-2H metabolite of C16-BAC.

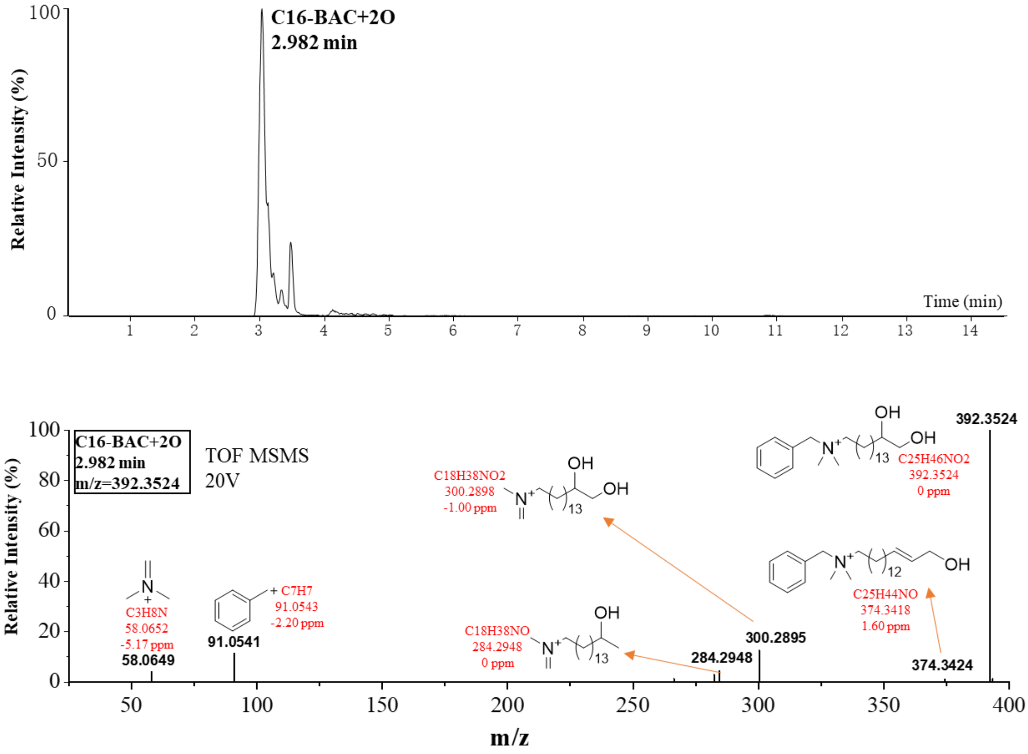

Figure S50. Chromatographic peak generated from m/z 392.3524 and MS/MS spectrum for +2O metabolite of C16-BAC.

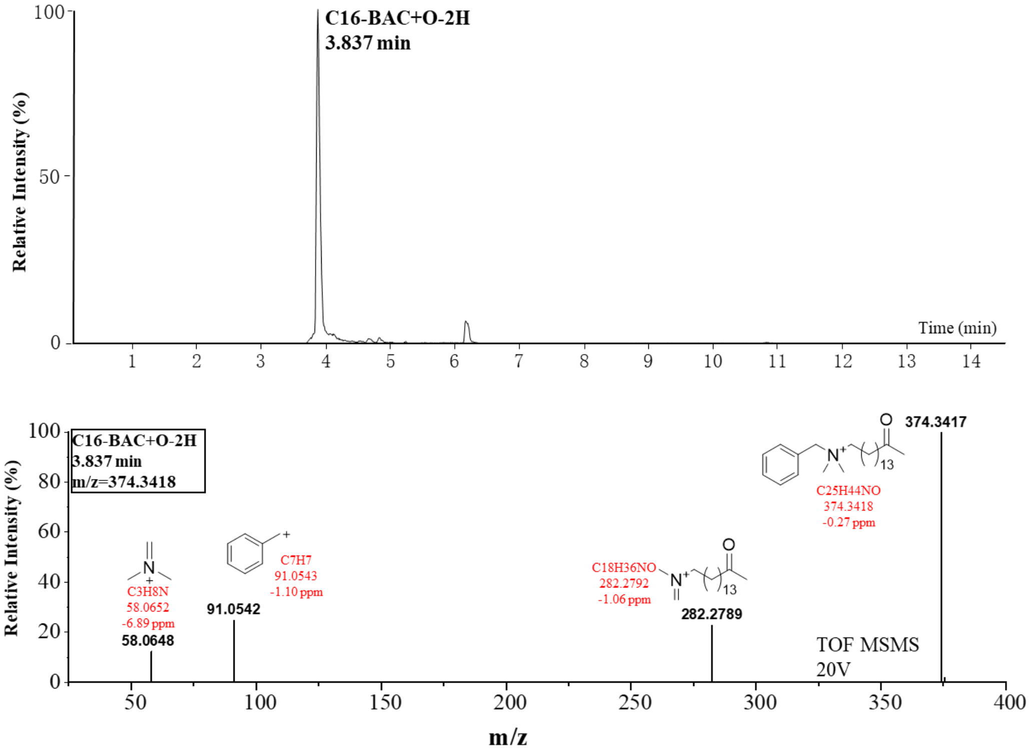

Figure S51. Chromatographic peak generated from m/z 374.3418 and MS/MS spectrum for +O-2H metabolite of C16-BAC.

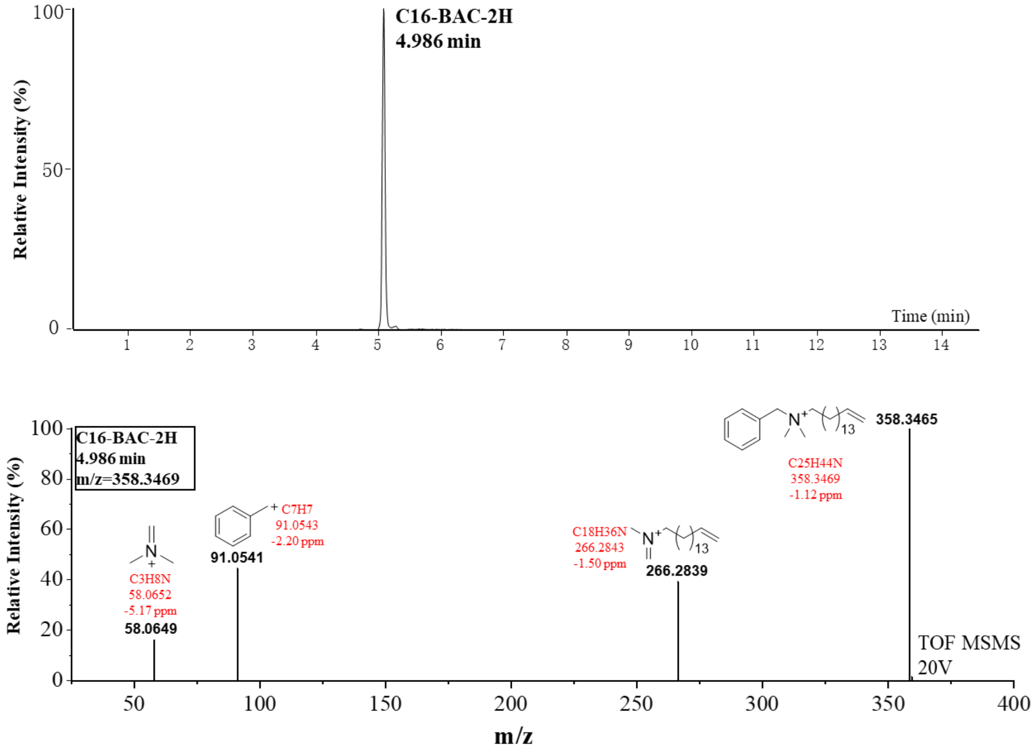

Figure S52. Chromatographic peak generated from m/z 358.3469 and MS/MS spectrum for -2H metabolite of C16-BAC.

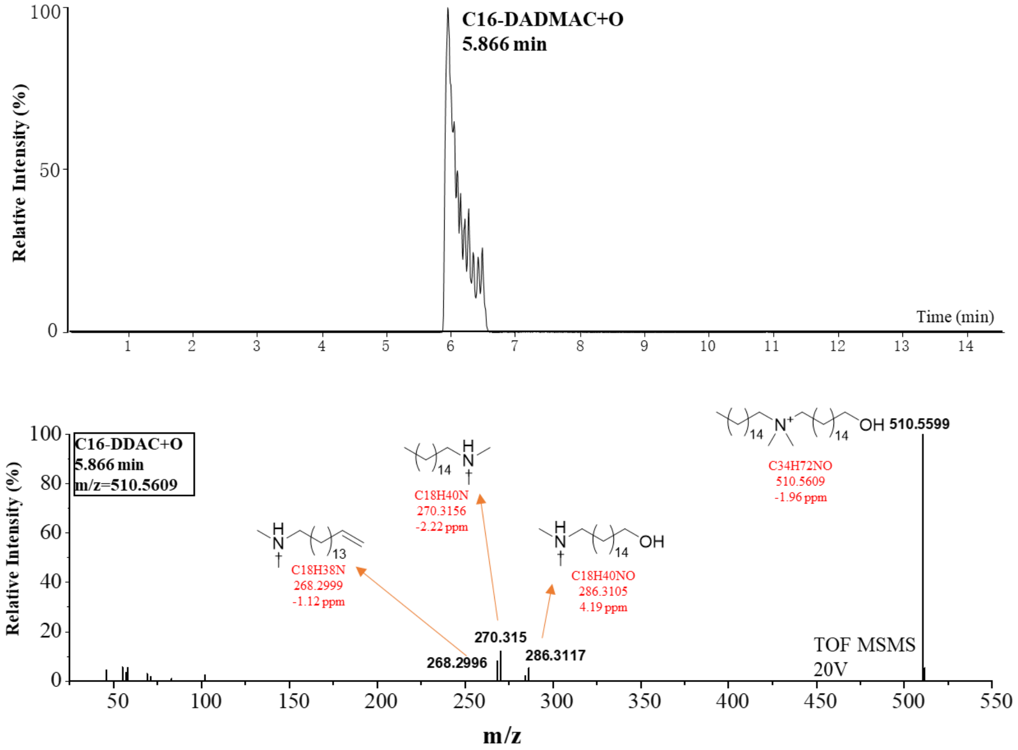

Figure S53. Chromatographic peak generated from m/z 510.5609 and MS/MS spectrum for +O metabolite of C16-DADMAC.

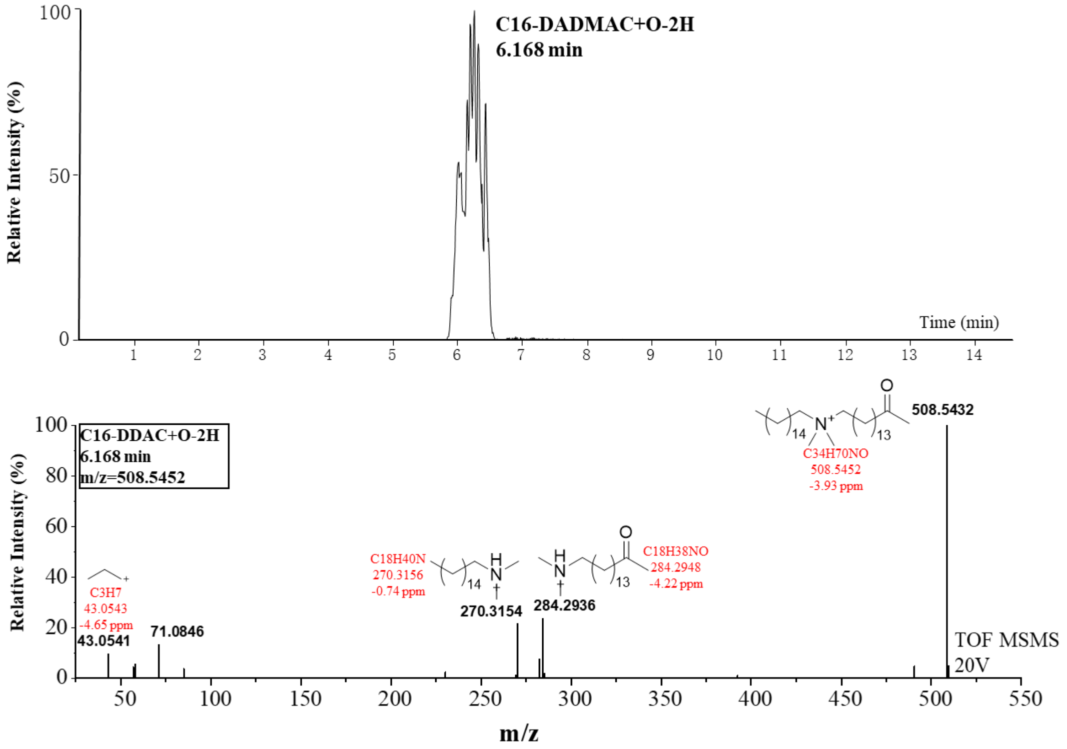

Figure S54. Chromatographic peak generated from m/z 508.5452 and MS/MS spectrum for +O-2H metabolite of C16-DADMAC.

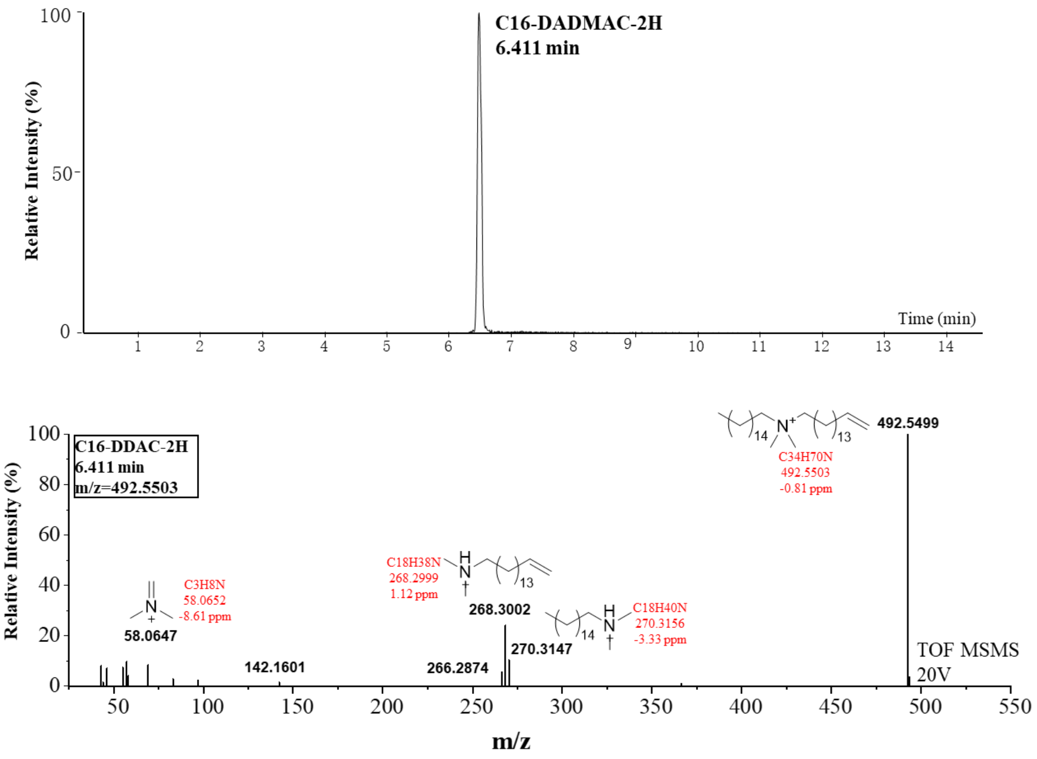

Figure S55. Chromatographic peak generated from m/z 492.5503 and MS/MS spectrum for -2H metabolite of C16-DADMAC.

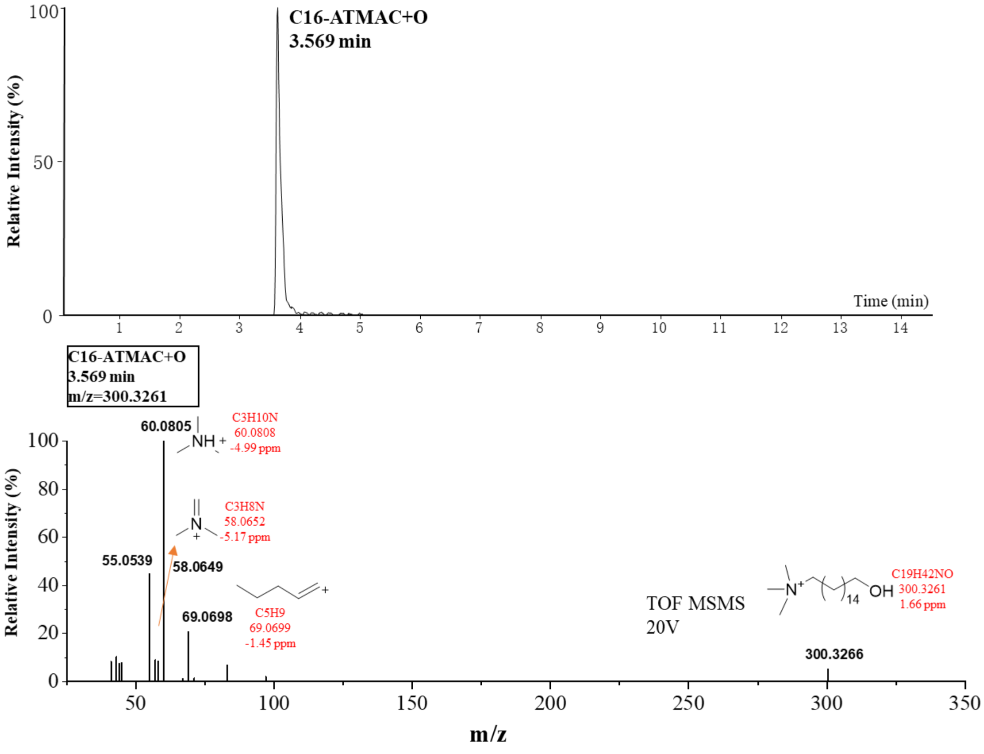

Figure S56. Chromatographic peak generated from m/z 300.3261 and MS/MS spectrum for +O metabolite of C16-ATMAC.

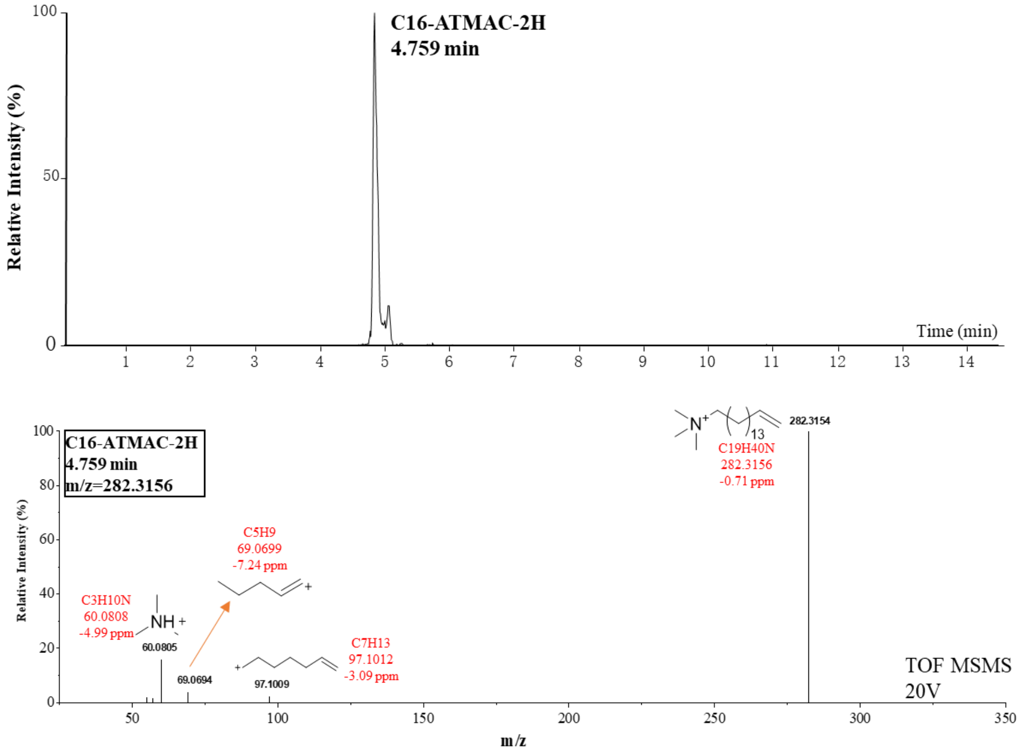

Figure S57. Chromatographic peak generated from m/z 282.3156 and MS/MS spectrum for -2H metabolite of C16-ATMAC.

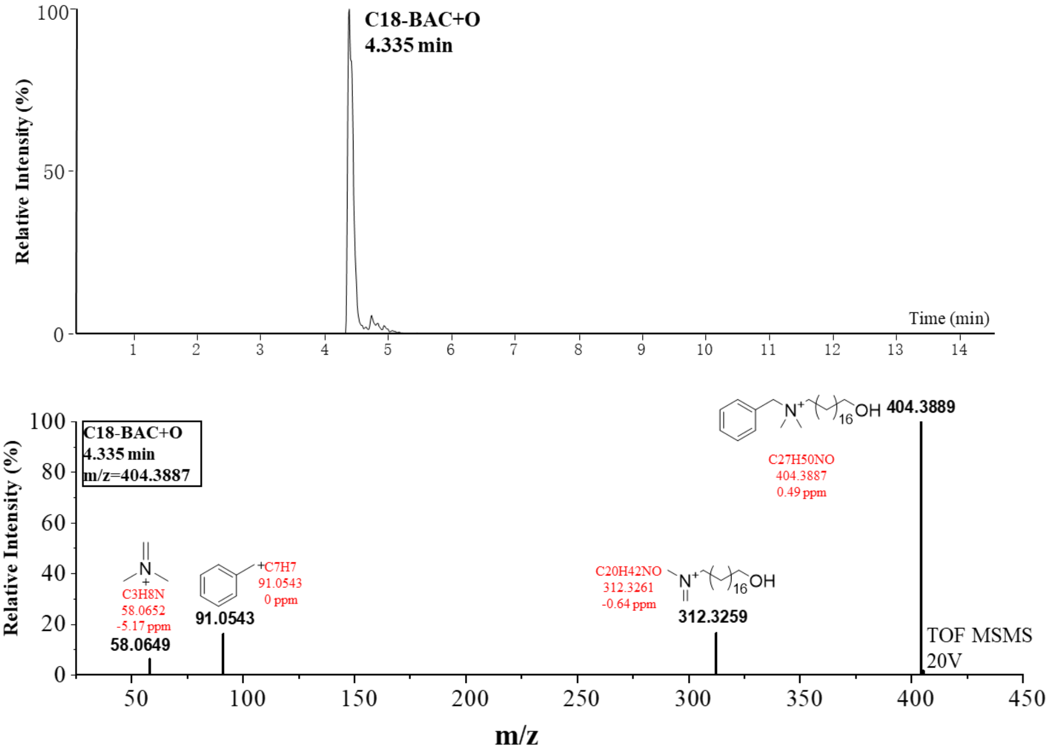

Figure S58. Chromatographic peak generated from m/z 404.3887 and MS/MS spectrum for +O metabolite of C18-BAC.

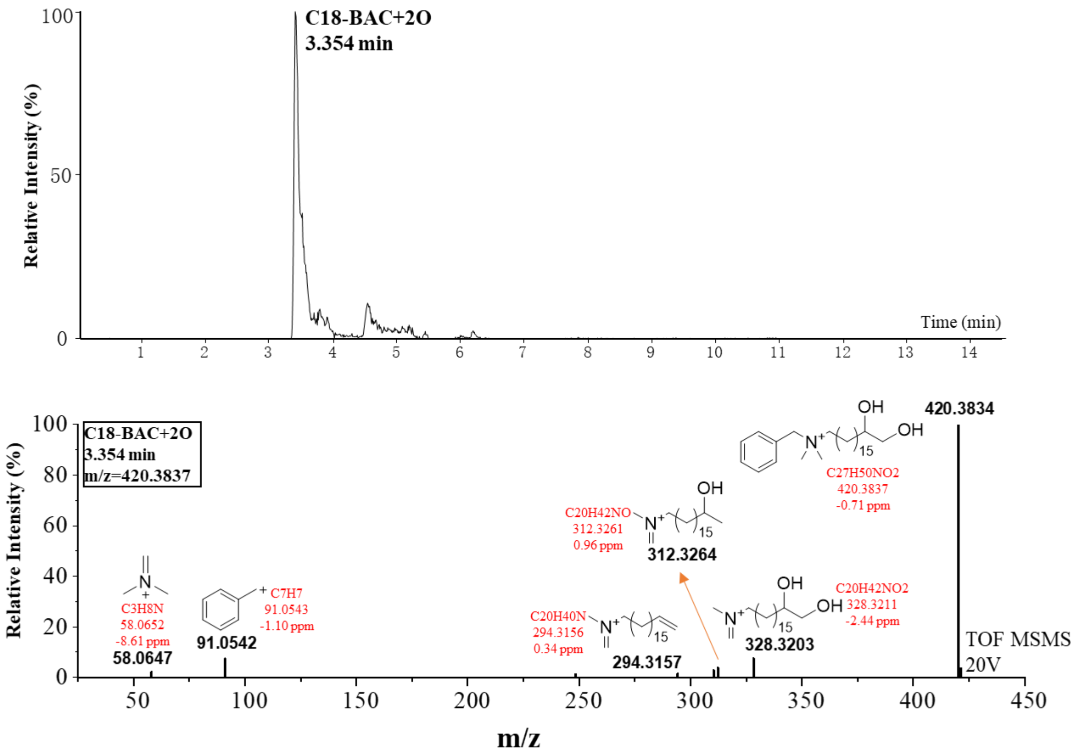

Figure S59. Chromatographic peak generated from m/z 420.3837 and MS/MS spectrum for +2O metabolite of C18-BAC.

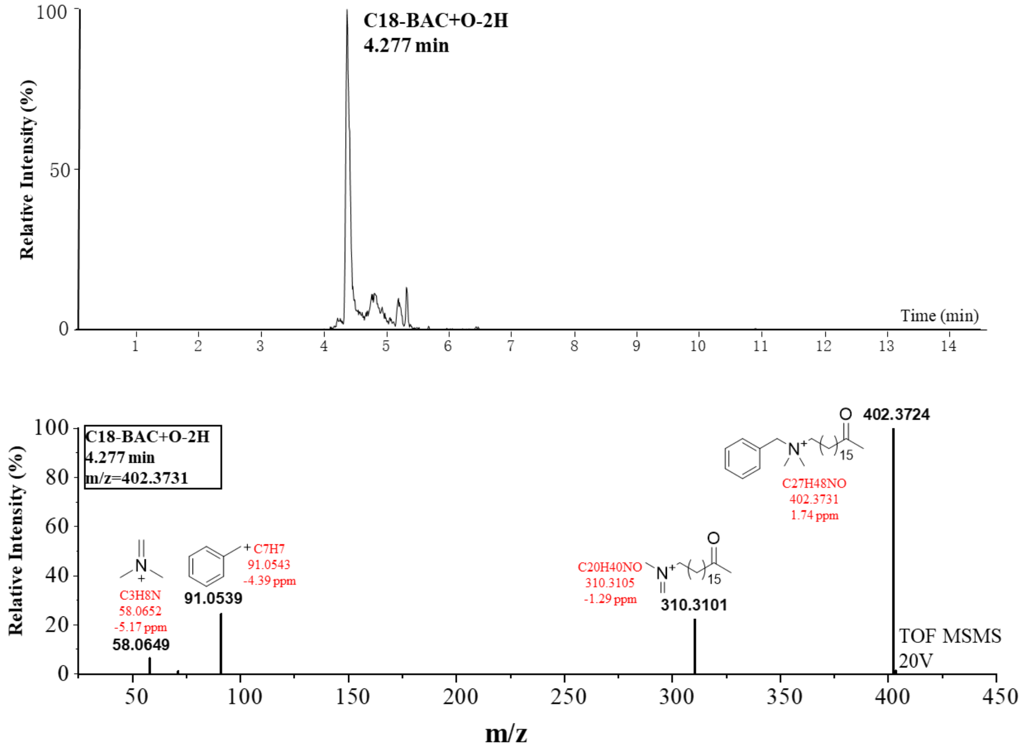

Figure S60. Chromatographic peak generated from m/z 402.3731 and MS/MS spectrum for +O-2H metabolite of C18-BAC.

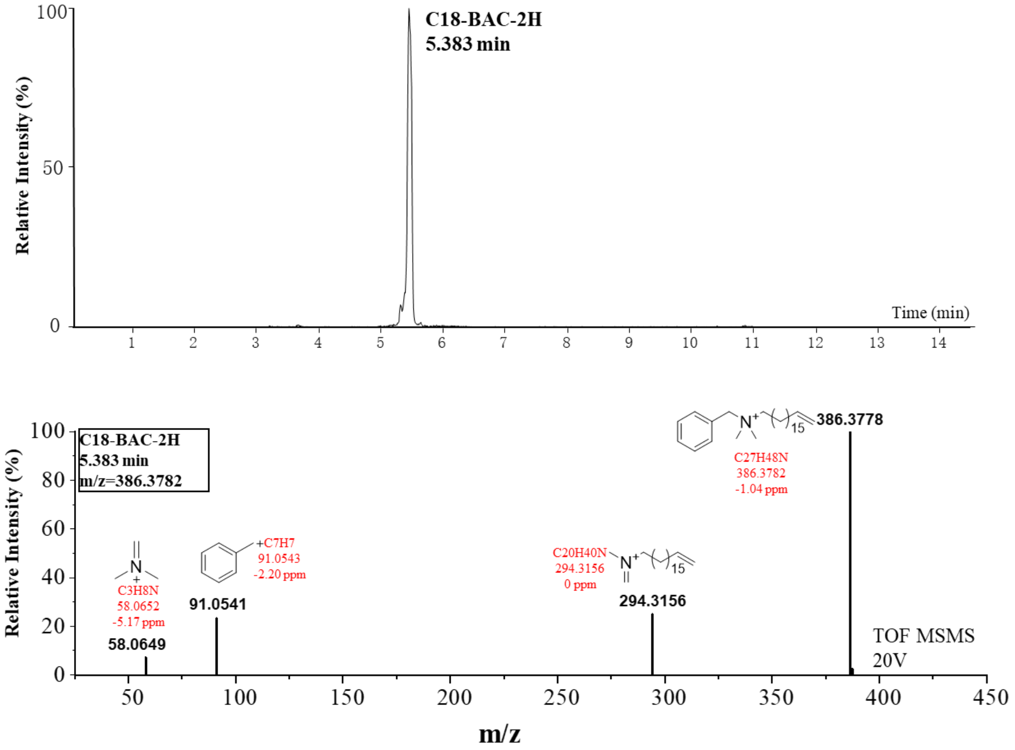

Figure S61. Chromatographic peak generated from m/z 386.3782 and MS/MS spectrum for -2H metabolite of C18-BAC.

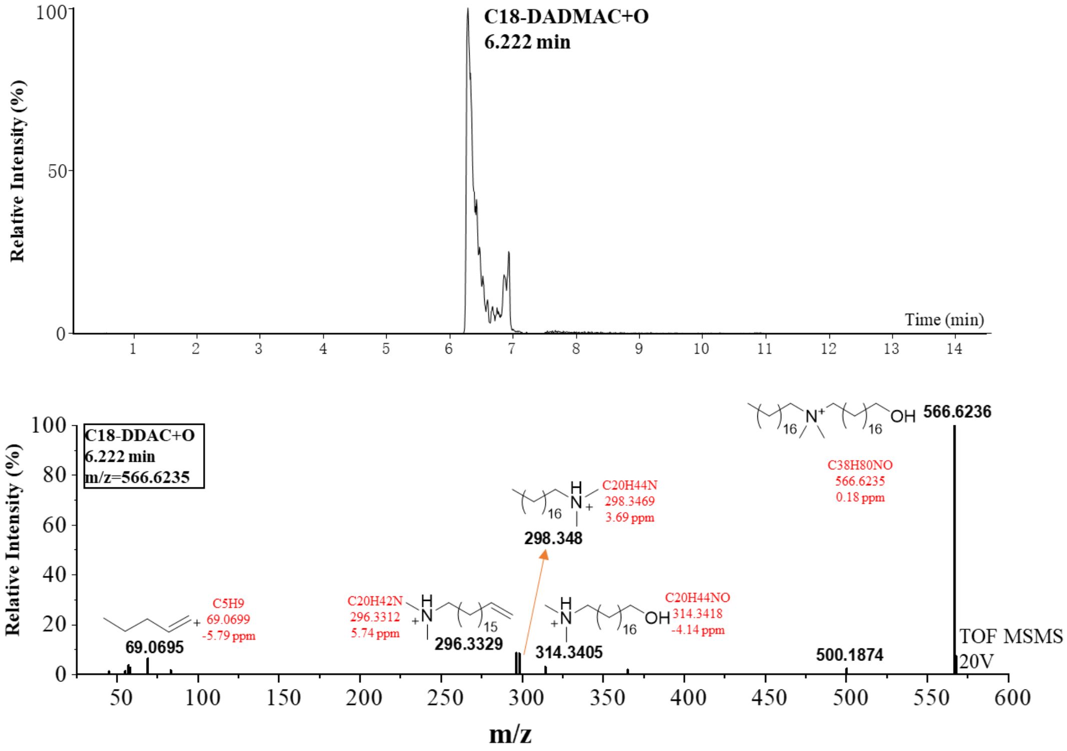

Figure S62. Chromatographic peak generated from m/z 566.6235 and MS/MS spectrum for +O metabolite of C18-DADMAC.

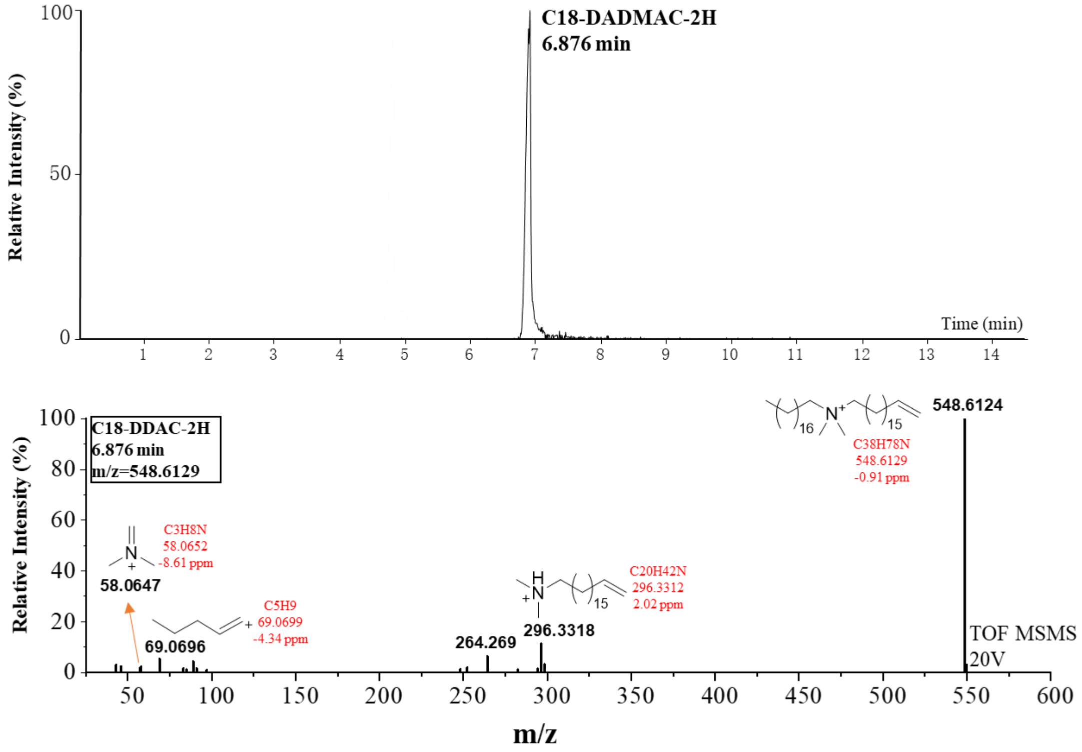

Figure S63. Chromatographic peak generated from m/z 548.6129 and MS/MS spectrum for -2H metabolite of C18-DADMAC.

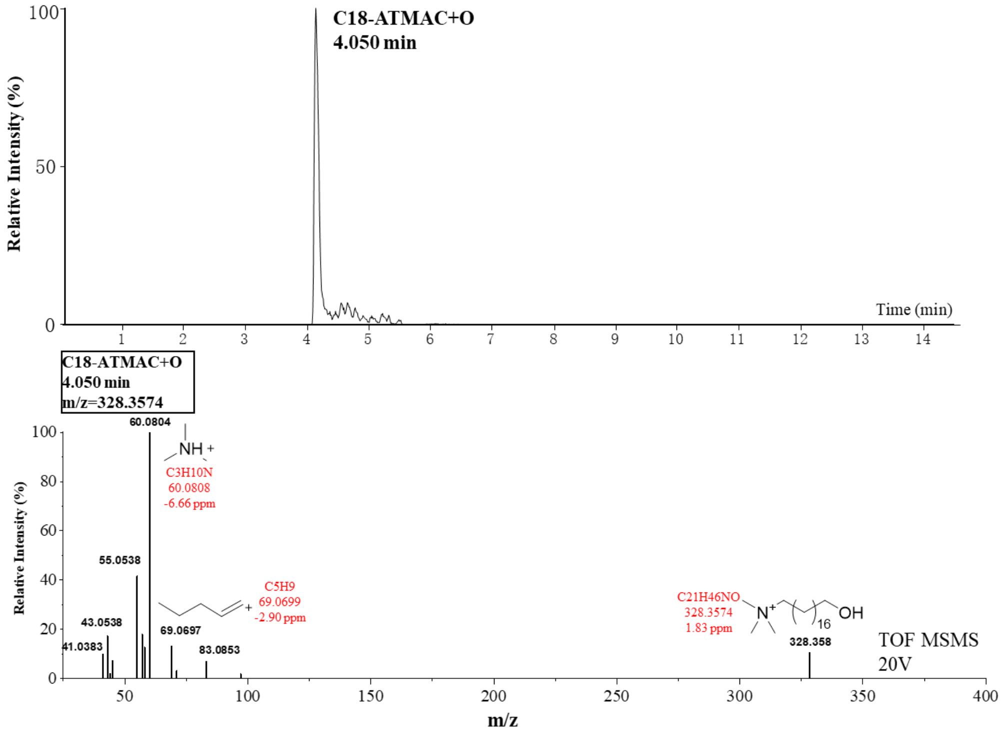

Figure S64. Chromatographic peak generated from m/z 328.3574 and MS/MS spectrum for +O metabolite of C18-ATMAC.

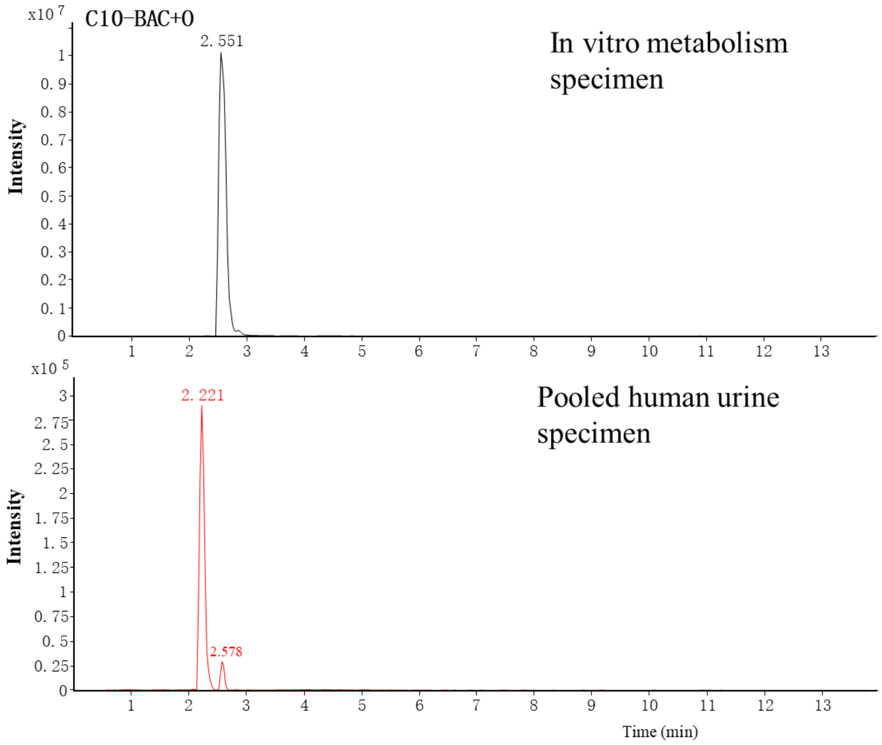

Figure S65. Metabolite signal of C10-BAC+O detected from *in vitro* specimen and pooled urine specimen.

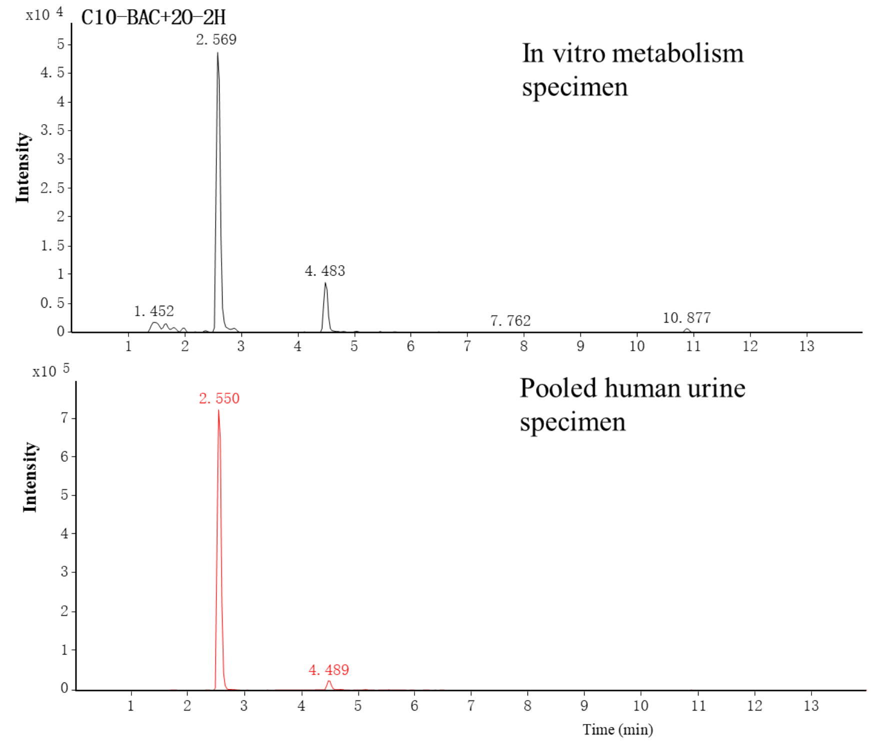

Figure S66. Metabolite signal of C10-BAC+O-2H detected from *in vitro* specimen and pooled urine specimen.

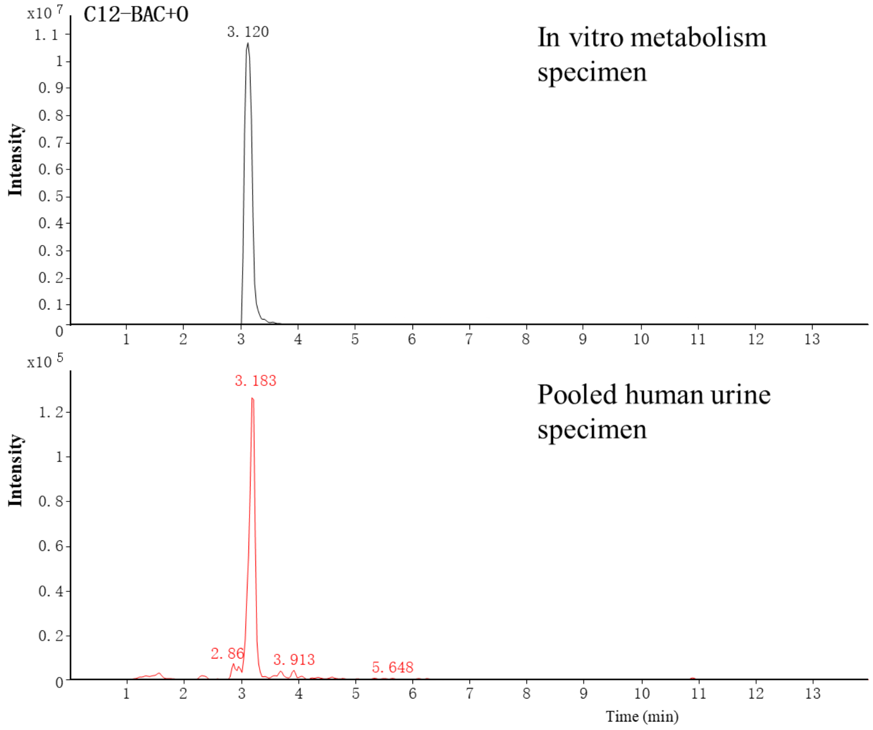

Figure S67. Metabolite signal of C12-BAC+O detected from *in vitro* specimen and pooled urine specimen.

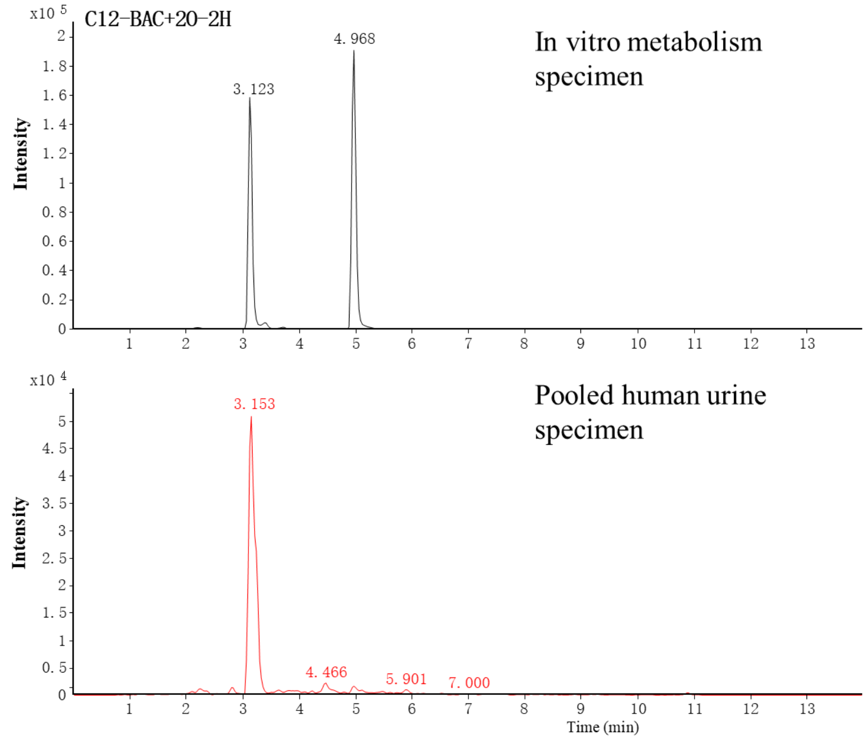

Figure S68. Metabolite signal of C12-BAC+O-2H detected from the *in vitro* specimen and the pooled urine specimen.

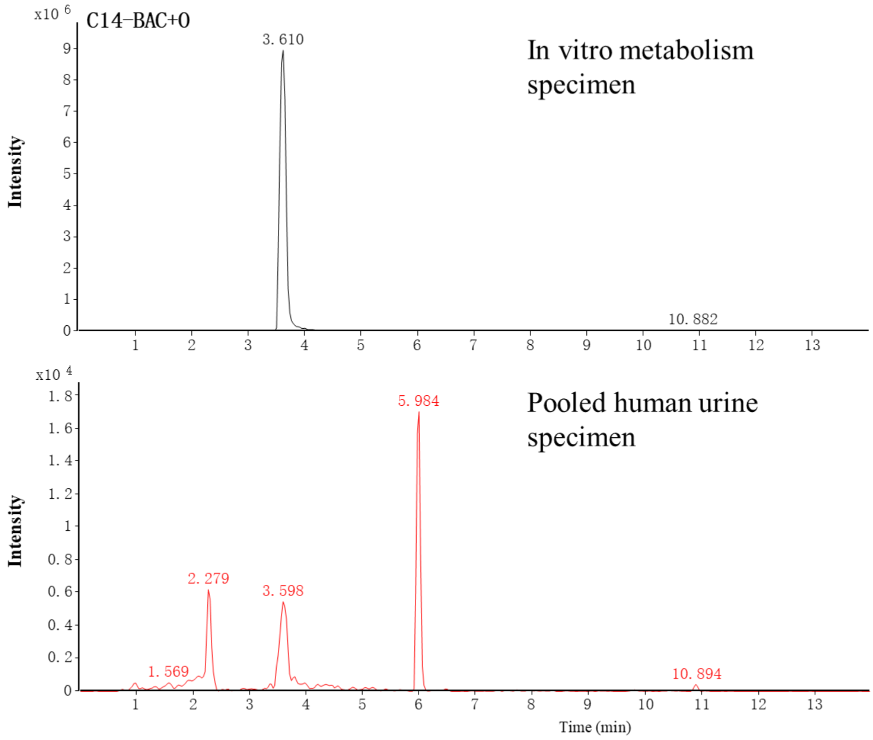

Figure S69. Metabolite signal of C12-BAC+O-2H detected from the *in vitro* specimen and the pooled urine specimen.

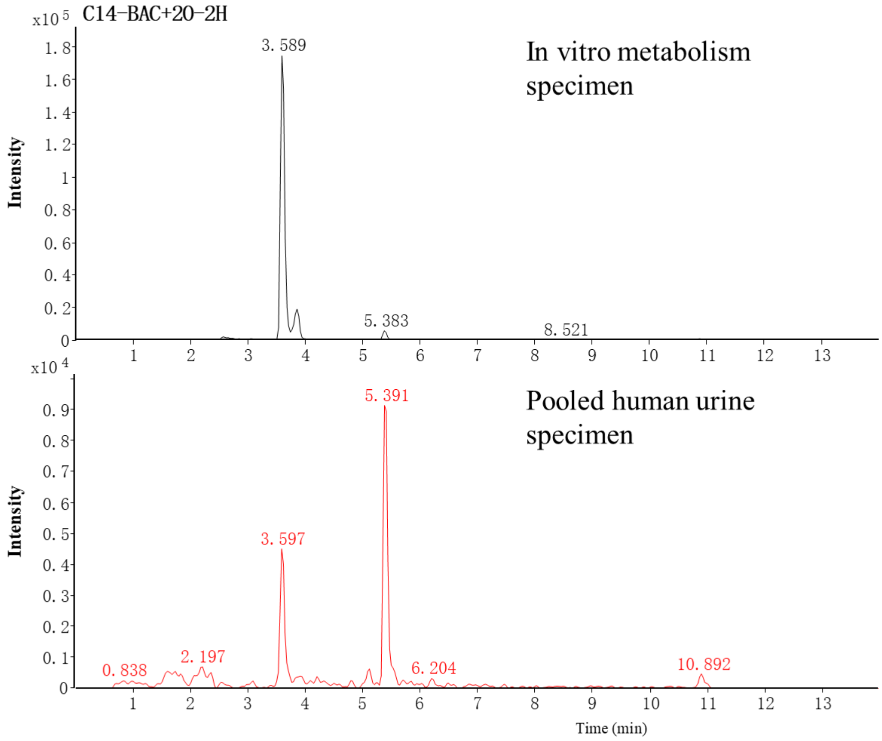

Figure S70. Metabolite signal of C14-BAC+2O-2H detected from *in vitro* specimen and the pooled urine specimen.

References

(1) Tang, J.; Lin, M.; Ma, S.; Yang, Y.; Li, G.; Yu, Y.; Fan, R.; An, T., Identifying dermal uptake as a significant pathway for human exposure to typical semivolatile organic compounds in an E-Waste dismantling site: The relationship of contaminant levels in handwipes and urine metabolites. *Environ Sci Technol* **2021,** *55*, 14026-14036.

(2) Phillips, A. L.; Hammel, S. C.; Hoffman, K.; Lorenzo, A. M.; Chen, A.; Webster, T. F.; Stapleton, H. M., Children's residential exposure to organophosphate ester flame retardants and plasticizers: Investigating exposure pathways in the TESIE study. *Environ Int* **2018,** *116*, 176-185.

(3) Hoffman, K.; Garantziotis, S.; Birnbaum Linda, S.; Stapleton Heather, M., Monitoring indoor exposure to organophosphate flame retardants: Hand wipes and house dust. *Environ Health Perspect* **2015,** *123*, 160-165.

(4) Cheng, Y.; Liu, C.; Lv, Z.; Liang, Y.; Xie, Y.; Wang, C.; Wan, S.; Leng, X.; Hu, M.; Zheng, G., High-resolution mass spectrometry screening of quaternary ammonium compounds (QACs) in dust from homes and various microenvironments in south China. *Environ Sci Technol* **2024,** *58*, 3182-3193.

(5) Hu, M.; Li, L.; Lv, Z.; Sangion, A.; Zheng, G.; Cai, Z.; Salamova, A., Quaternary ammonium compounds in paired samples of blood and indoor dust from the United States. *Environ Sci Tech Let* **2024,** *11*, 1308-1313.

(6) Hammel, S. C.; Hoffman, K.; Phillips, A. L.; Levasseur, J. L.; Lorenzo, A. M.; Webster, T. F.; Stapleton, H. M., Comparing the use of silicone wristbands, hand wipes, and dust to evaluate children's exposure to flame retardants and plasticizers. *Environ Sci Technol* **2020,** *54*, 4484-4494.

(7) Levasseur, J. L.; Hammel, S. C.; Hoffman, K.; Phillips, A. L.; Zhang, S.; Ye, X.; Calafat, A. M.; Webster, T. F.; Stapleton, H. M., Young children's exposure to phenols in the home: Associations between house dust, hand wipes, silicone wristbands, and urinary biomarkers. *Environ Int* **2021,** *147*, 106317.

(8) Okeme, J. O.; Saini, A.; Yang, C.; Zhu, J.; Smedes, F.; Klánová, J.; Diamond, M. L., Calibration of polydimethylsiloxane and XAD-Pocket passive air samplers (PAS) for measuring gas- and particle-phase SVOCs. *Atmos Environ* **2016,** *143*, 202-208.

(9) Zheng, G.; Filippelli, G. M.; Salamova, A., Increased indoor exposure to commonly used disinfectants during the COVID-19 Pandemic. *Environ Sci Tech Let* **2020,** *7*, 760-765.

(10) Dong, Z.; Li, T.; Wan, Y.; Sun, Y.; Hu, J., Physiologically based pharmacokinetic modeling for chlorinated paraffins in rats and humans: Importance of biliary excretion. *Environ Sci Technol* **2020,** *54*, 938-946.

(11) Poothong, S.; Padilla-Sánchez, J. A.; Papadopoulou, E.; Giovanoulis, G.; Thomsen, C.; Haug, L. S., Hand wipes: A useful tool for assessing human exposure to poly- and perfluoroalkyl substances (PFASs) through hand-to-mouth and dermal contacts. *Environ Sci Technol* **2019,** *53*, 1985-1993.

(12) Ma, S.; Hu, X.; Tang, J.; Cui, J.; Lin, M.; Wang, F.; Yang, Y.; Yu, Y., Urinary metabolites and handwipe phthalate levels among adults and children in southern China: Implication for dermal exposure. *J Hazard Mater* **2022,** *439*, 129639.

(13) Bohlin, P.; Audy, O.; Škrdlíková, L.; Kukučka, P.; Vojta, Š.; Přibylová, P.; Prokeš, R.; Čupr, P.; Klánová, J., Evaluation and guidelines for using polyurethane foam (PUF) passive air samplers in double-dome chambers to assess semi-volatile organic compounds (SVOCs) in non-industrial indoor environments. *Environ Sci-Proc Imp* **2014,** *16*, 2617-2626.
(14) Shoeib, M.; Harner, T., Characterization and comparison of three passive air samplers for persistent organic pollutants. *Environ Sci Technol* **2002,** *36*, 4142-4151.
(15) Tromp, P. C.; Beeltje, H.; Okeme, J. O.; Vermeulen, R.; Pronk, A.; Diamond, M. L., Calibration of polydimethylsiloxane and polyurethane foam passive air samplers for measuring semi volatile organic compounds using a novel exposure chamber design. *Chemosphere* **2019,** *227*, 435-443.
(16) Belova, L.; Musatadi, M.; Gys, C.; Roggeman, M.; den Ouden, F.; Olivares, M.; van Nuijs, A. L. N.; Poma, G.; Covaci, A., In vitro metabolism of quaternary ammonium compounds and confirmation in human urine by liquid chromatography ion-mobility high-resolution mass spectrometry. *Environ Sci Technol* **2024,** *58*, 16785-16794.
(17) Seguin, R. P.; Herron, J. M.; Lopez, V. A.; Dempsey, J. L.; Xu, L., Metabolism of benzalkonium chlorides by human hepatic cytochromes P450. *Chem Res Toxicol* **2019,** *32*, 2466-2478.
(18) Li, Z. M.; Lakuleswaran, M.; Kannan, K., LC-MS/MS methods for the determination of 30 quaternary ammonium compounds including benzalkonium and paraquat in human serum and urine. *J Chromatogr B* **2023,** *1214*, 123562.
(19) EPA, U. S. *Exposure Factors Handbook 2011 Edition, EPA/600/R-09/052F*; U.S. Environmental Protection Agency: Washington, DC, **2011**.
(20) Hammel, S. C.; Andersen, H. V.; Knudsen, L. E.; Frederiksen, M., Inhalation and dermal absorption as dominant pathways of PCB exposure for residents of contaminated apartment buildings. *Int J Hyg Envir Heal* **2023,** *247*, 114056.
(21) Leng, X. Identification of quaternary ammonium compounds in textiles and their environmental and health risk assesment (Master's thesis). Southern University of Science and Technology, **2025**.
(22) Stapleton, H. M.; Kelly, S. M.; Allen, J. G.; McClean, M. D.; Webster, T. F., Measurement of polybrominated diphenyl ethers on hand wipes: Estimating exposure from hand-to-mouth contact. *Environ Sci Technol* **2008,** *42*, 3329-3334.
(23) Zhao, L.; Lu, Y.; Zhu, H.; Cheng, Z.; Wang, Y.; Chen, H.; Yao, Y.; Zhang, J.; Li, X.; Sun, Z.; Zhang, C.; Sun, H., E-waste dismantling-related occupational and routine exposure to melamine and its derivatives: Estimating exposure via dust ingestion and hand-to-mouth contact. *Environ Int* **2022,** *165*, 107299.
